# One size does not fit all: HIV prevalence and correlates of risk for men who have sex with men, transgender women in multiple cities in Papua New Guinea

**DOI:** 10.1186/s12889-019-6942-7

**Published:** 2019-05-22

**Authors:** Avi J. Hakim, Kelsey Coy, Steven G. Badman, Barne Willie, Rebecca Narokobi, Josephine Gabuzzi, Simon Pekon, Martha Kupul, Parker Hou, Herick Aeno, Ruthy Neo Boli, Joshua Nembari, Sophie Ase, Angelyne Amos, Nick Dala, Damian Weikum, Steven Callens, John M. Kaldor, Andrew J. Vallely, Angela Kelly-Hanku, on behalf of the Kauntim mi tu Study Team

**Affiliations:** 1 US Centers for Disease Control and Prevention, Division of Global HIV and Tuberculosis, 1600 Clifton Rd, NE, Atlanta, GA 30329 USA; 2 Kirby Institute, UNSW Sydney, Wallace Wurth Building, High St, Kensington, NSW 2052 Australia; 3 Papua New Guinea Institute of Medical Research, Homat Street, Goroka, Eastern Highlands Province 441 Papua New Guinea; 4 Papua New Guinea National Department of Health, AOPI Centre, Waigani Drive, P O Box 807, Waigani, Port Moresby, National Capital District 131 Papua New Guinea; 5 University of Ghent, Sint-Pietersnieuwstraat 25, 9000 Ghent, East Flanders Belgium

**Keywords:** Papua New Guinea, HIV correlates, Men who have sex with men, Transgender women, Respondent-driven, sampling.

## Abstract

**Background:**

Biobehavioral data about men who have sex with men (MSM) and transgender women (TGW) in Papua New Guinea (PNG) are limited to those who sell sex. Information about those MSM and TGW who do not sell sex is necessary to guide HIV prevention and treatment efforts.

**Methods:**

We conducted respondent-driven sampling (RDS) surveys among MSM and TGW in Port Moresby, Lae, and Mt. Hagen, PNG from in 2016 and 2017. Eligibility criteria was: aged > 12 years, born male, could speak English or *Tok Pisin* and had oral or anal sex with another person born male in the past 6 months. Participants were interviewed face-to-face and offered rapid HIV testing. Weighted data analysis was conducted using RDS-Analyst (v. 0.62).

**Results:**

We enrolled 400 participants in Port Moresby, 352 in Lae, and 111 in Mt. Hagen. In the last six months, 73.2% of MSM/TGW in Port Moresby, 77.9% in Lae, and 75.9% in Mt. Hagen, had a concurrent sexual partnership. Upwards of 70% of MSM/TGW in all three cities had sex with a woman in the same period. Less than half of MSM/TGW had ever tested for HIV. HIV prevalence among MSM/TGW was 8.5% in Port Moresby and 6.9% in Lae. Among participants in Mt. Hagen it was 1.3%. HIV was associated with not having sex with a woman in the last six months and sexually transmitted disease symptoms in the last 12 months in Port Moresby and Lae. In Port Moresby, it was also associated with an uncut foreskin, and in Lae with earning income in the formal sector and being unable to rely on other MSM or TGW to accompany them to healthcare services.

**Conclusions:**

The large proportion of MSM and TGW with concurrent sexual partnerships, combined with the low testing coverage, indicates strong potential for the spread of HIV. The different correlates of HIV in Port Moresby and Lae highlight the importance of conducting surveys in multiple locations and using data to develop locally appropriate interventions even within a country.

**Electronic supplementary material:**

The online version of this article (10.1186/s12889-019-6942-7) contains supplementary material, which is available to authorized users.

## Background

Nearly four decades since the emergence of HIV, there are still settings and populations whose HIV epidemics we know little about [1, 2]. Papua New Guinea (PNG) is one such example. It was previously described as having a generalized epidemic. The expansion of antenatal clinic surveillance data, as well as biobehavioural surveys, have resulted in a better understanding of PNG’s HIV epidemic and a consensus has emerged that with an HIV prevalence estimated at 0.9%, the country is experiencing an epidemic concentrated the key populations of sex workers, men who have sex with men (MSM), and transgender women (TGW) [3–8]. The data that exist on MSM and TGW in PNG focuses on those who sell sex, rather than all MSM and TGW. This new understanding of the epidemic in PNG necessitates the collection of data on key populations to inform estimates and guide the country’s HIV response.

Male-to-male sex remains illegal and highly stigmatized in PNG, hampering HIV service provision and uptake for these men. One of the first respondent-driven sampling (RDS) surveys in the world was conducted among MSM in PNG in 2005 but it did not assess HIV prevalence [7]. It did, however, highlight low condom use and experiences of discrimination among MSM. A 2010 survey of MSM had similar findings but similarly did not include HIV testing [8]. In the same year, a survey of females, males, and transgender women engaged in sex work in the capital of Port Moresby found HIV prevalence in these populations of 19.0, 8.8, 23.7%, respectively [9]. These findings helped reorient PNG’s HIV response to focus on key populations, but until now there has been little information to guide the epidemic response [10, 11].

Data about MSM and TGW in PNG are needed to guide the country’s HIV strategy and service provision. In conjunction with the PNG National Department of Health and National AIDS Council Secretariat, we conducted a respondent-driven sampling (RDS) biobehavioural surveys (BBS) of MSM and TGW in Port Moresby to fill this information gap. Here we report on HIV prevalence and correlates of HIV infection.

## Methods

### Community consultation

Community consultation was undertaken with MSM and TGW in Port Moresby, Lae, and Mt. Hagen to guide survey preparation and build trust with local stakeholders. Community consultation indicated that recruiting MSM and TGW would be challenging and both populations found it acceptable to combine the two populations into one survey sample.

### Study population, setting, and design

We conducted RDS BBS of MSM and TGW in Port Moresby from June to October 2016, in Lae from January to June 2017, and Mt. Hagen from August to December 2017. These cities were selected for the survey because they are the most populous and include the national capital (Port Moresby), the main economic city and port (Lae), and a city that is at the intersection of roads and natural resource extraction activities in the country (Mt. Hagen). RDS is a variant of snowball sampling that can be used to produce sampling weights and approximate a random sample [12–14]. Eligibility criteria were: age > 12 years, spoke English or Tok Pisin, born biologically male, had oral or anal sex with a male in the past 6 months, and be in possession of a valid study coupon.

### Recruitment

Recruitment started with four seeds in Port Moresby, four in Lae, and five in Mt. Hagen. Twelve additional seeds were added in Port Moresby, 15 in Lae, and 4 in Mt. Hagen to facilitate recruitment. Seeds were purposely selected to create diversity with respect to: age, sexual/gender identity, place of residence, region of origin, marital status, receipt of a unique object for size estimation, and affiliation with a non-governmental or community-based organization.

### Data collection

Candidate participants were screened for eligibility and those eligible were asked to provide verbal informed consent. Non-blood specimens were collected for sexually transmitted infection and tuberculosis testing after which participants engaged in a computer-assisted personal interview (Open Data Kit, Washington, US).

After the interview, participants received pre-test HIV counseling before providing written informed consent for HIV testing. Fifteen milliliters of blood was taken through venipuncture for HIV testing and, if positive by confirmatory testing, CD4 T-cell count and molecular HIV viral load testing were also conducted at the point-of-care. The PNG national algorithm for HIV testing was used: Determine HIV-1/2 (Alere, MA, USA) followed by confirmatory testing with HIV 1/2 Stat-Pak (Chembio, Medford, NY). Discordant results between the two tests were deemed inconclusive, and participants were advised to retest in 3 weeks. HIV external quality assurance panels were provided by The Royal College of Pathologists of Australasia. We used the Chembio DPP Syphilis Assay to test for syphilis (Chembio, Medford, NY). All test results were returned to participants at the end of the first study visit. HIV-positive participants were offered an escort by a peer navigator to HIV treatment services of their choosing that had been sensitized to work with key populations. Those with an active syphilis infection initiated treatment at the survey site and were given a referral to complete treatment. Study staff were trained to identify and refer all sexually exploited persons under the age of 18 years to partner organizations experienced in providing counseling, health, social, and other protective services to these populations.

While waiting for test results, participants in Port Moresby received 3 coupons with which to recruit peers. Midway through data collection the number of coupons was increased to 4 to increase recruitment. Participants in Lae received three coupons to recruit peers and in Mt. Hagen they received four. In all locations, participants received 45 PNG kina for their first visit (about $14 US) and 10 PNG kina (about $3 US) per successful recruit plus 5 PNG kina (about $1.50 US) for transportation at their second visit. All participants were also provided with information on HIV and other sexually transmitted infections, condoms, and lubricants.

### Data measures

The questionnaire used as its foundation the WHO, CDC, UNAIDS, and FHI360 Biobehavioral Survey Guidelines for Populations at Risk for HIV [15]. Interview domains included demographics, sexual history and identity, condom use, stigma, social cohesion, violence, HIV knowledge, history of sexually transmitted diseases, penile modification, internalized homophobia, and uptake of health services. The two-item Patient Health Questionnaire (PHQ-2) was used to screen for depression [16]. Internalized homophobia was defined based on responses to five questions regarding their feelings about sexual attraction to men. Comprehensive awareness of HIV was based on the United Nations Joint Programme for HIV/AIDS definition of correctly answering three questions and rejecting two myths regarding HIV [17]. Partner concurrency was defined as overlapping sexual partnerships with partners of any sex where sexual intercourse with one partner occurred between two acts of intercourse with another partner, limited to the last three sexual partners in the last six months. The full questionnaires can be found in the Additional files 1 and 2 (FSW Questionnaire and MSM TGW Questionnaire).

### Data analysis

Our analysis characterizes MSM and TGW and correlates of HIV infection among these populations in each of the three survey cities. Odds ratios (OR) and 95% confidence intervals (CI) were calculated for bivariate comparisons and a *p*-value < 0.1 was the threshold for inclusion in multivariate analysis to identify a final model. To remain in the final model, the least significant variable was eliminated until all remaining variables were significant at the 0.05 level. Additional variables were removed to lower the relative standard error.

In Port Moresby, the full model included education, gender identity, disclosed sexual identity to non-MSM, ever cut foreskin, sex with a woman in the last 6 months, self-reported sexually transmitted disease (STD) symptoms, used the internet or mobile application to meet people, and last contact with peer outreach.

In Lae, the full model included main source of income, disclosure of sexual behaviors to non-MSM, can rely on other MSM or TGW accompany them to the doctor, experienced physical violence in the last 12 months, sex with a woman in the last 6 months, had self-reported STD symptoms, and last contact with peer outreach. No model was produced for Mt. Hagen because the actual sample size was too small and convergence was not reached for HIV [18].

Data were weighted and analyzed using Respondent-Driven Sampling Analyst (RDS-A) version 0.62 (Los Angeles, CA) and SAS version 9.3 (Carey, NC). We utilized Giles’ Successive Sampling Estimator in RDS-A. All data presented are RDS-adjusted population estimates unless otherwise indicated. Weights were imported from RDS-A, and survey logistic procedures were used in SAS to identify correlates of HIV infection.

### Ethical approval

This survey was approved by the PNG National Department of Health’s Medical Research Advisory Committee (MRAC), the Research Advisory Committee of the National AIDS Council Secretariat (RAC), the PNG Institute of Medical Research’s Institutional Review Board (IRB), and the Human Research Ethics Committee at UNSW Sydney, Australia. The protocol was reviewed according to the Centers for Disease Control and Prevention’s (CDC) human research protection procedures and was determined to be research but CDC was not engaged. A letter of support was provided by Kapul Champion, the peer led civil society for sexually diverse men and transgender people.

## Results

We enrolled 400 participants (354 MSM and 46 TGW) in Port Moresby, 352 in Lae (325 MSM and 27 TGW), and 111 in Mt. Hagen (104 MSM and 7 TGW). We distributed 1348 coupons in Port Moresby, 1044 in Lae, and 444 in Mt. Hagen. The longest recruitment chain in each city had 26, 19, and 10 recruitment waves, respectively.

In all three cities male identity was more common than transgender identity and the majority of MSM and TGW were between the ages of 20–29 years (Table 1). The median age was highest in Port Moresby and lowest in Mt. Hagen. MSM and TGW in Lae were less educated than in the other two cities. Between 60 and 80% had never been married in all three cities. A higher proportion of MSM and TGW in Lae and Mt. Hagen (32.5 and 39.8%, respectively) had spent more than a month away from home in the last six months than in Port Moresby (16.6%). Over half of MSM and TGW in all cities cut their foreskin.

**Table 1:** Characteristics of men who have sex with men (MSM) and transgender women (TGW) in Port Moresby, Lae and Mt. Hagen

	PORT MORESBY			LAE			MOUNT HAGEN		
	Valid	Sample proportion (unweighted)	Population proportion (weighted)	Valid	Sample proportion (unweighted)	Population proportion (weighted)	Valid	Sample proportion (unweighted)	Population proportion (weighted)
	*N* = 400	%	% (95% CI)	*N* = 352	%	% (95% CI)	*N* = 111	%	% (95% CI)
Gender identity	400			352			111		
Male	354	88.5	89.4 (85.8–92.9)	325	92.3	93.2 (90.3–96.2)	104	93.7	94.3 (89.6–99.0)
TG	46	11.5	10.6 (7.1–14.2)	27	7.7	6.8 (3.8–9.7)	7	6.3	5.7 (1.0–10.4)
Age (years)	400			345			111		
12–19	36	9.0	11.4 (7.2–15.6)	45	13.0	14.1 (9.7–18.5)	32	28.8	24.3 (15.6–33.1)
20–24	107	26.8	26.3 (21.0–31.6)	107	31.0	32.0 (26.1–37.8)	51	45.9	48.1 (37.4–58.7)
25–29	113	28.3	26.3 (21.0–31.7)	90	26.1	26.0 (20.5–31.5)	11	9.9	11.5 (4.5–18.6)
30–34	63	15.8	17.1 (12.3–21.8)	54	15.7	13.8 (9.6–17.9)	8	7.2	8.3 (2.3–14.3)
35 or older	81	20.3	18.9 (14.2–23.6)	49	14.2	14.2 (9.8–18.7)	9	8.1	7.7 (2.2–13.2)
Sample Median (IQR)	27 (23–33)			25 (22–30)			21 (19–25)		
Education	400			352			111		
No formal education	36	9.0	8.7 (5.3–12.1)	62	17.6	18.2 (13.3–23.1)	8	7.2	6.5 (1.6–11.4)
Primary	203	50.8	48.8 (42.7–54.9)	140	39.8	40.8 (34.7–46.9)	37	33.3	38.7 (28.2–49.3)
High school or higher	161	40.3	42.5 (36.4–48.6)	150	42.6	41.0 (35.0–47.1)	66	59.5	54.8 (44.1–65.4)
Marital status	400			352			111		
Never married	246	61.5	62.5 (56.5–68.4)	240	68.2	70.7 (65.2–76.2)	88	79.3	77.9 (68.9–86.9)
Married	77	19.2	18.2 (13.4–23.0)	53	15.1	13.9 (9.6–18.1)	14	12.6	13.6 (6.1–21.1)
Divorced, separated, or widowed	77	19.3	19.3 (14.5–24.2)	59	16.8	15.4 (11.1–19.7)	9	8.1	8.4 (2.4–14.4)
Away from home for more than a month at a time, last 6 months	377			343			104		
Yes	70	18.6	16.6 (12.1–21.2)	118	34.4	32.5 (26.7–38.3)	36	34.6	39.8 (28.8–50.7)
No	307	81.4	83.4 (78.8–87.9)	225	65.6	67.5 (61.7–73.3)	68	65.4	60.2 (49.3–71.2)
Main source of income	380			340			102		
Formal sector	94	24.7	23.6 (18.3–28.8)	94	27.6	26.5 (21.0–32.0)	14	13.7	15.6 (7.2–24.0)
Informal sector	153	40.3	39.3 (33.1–45.5)	149	43.8	45.6 (39.4–51.9)	40	39.2	42.9 (31.8–54.1)
Unemployed	133	35.0	37.1 (31.0–43.3)	97	28.5	27.8 (22.2–33.5)	48	47.1	41.4 (30.7–52.3)
Average monthly income	266			255					
< 200 kina (~USD 63)	34	12.8	11.7 (7.1–16.4)	17	6.7	7.6 (3.6–11.5)	10	15.9	14.1 (4.5–23.7)
200–499 kina	94	35.3	34.1 (27.0–41.2)	121	47.5	48.0 (40.8–55.3)	23	36.5	38.8 (25.0–52.6)
500–999 kina	94	35.3	39.2 (31.7–46.7)	80	31.4	29.9 (23.3–36.4)	15	23.8	21.3 (10.0–32.6)
≥ 1000 kina	44	16.5	15.0 (9.6–20.3)	37	14.5	14.5 (9.4–19.6)	15	23.8	25.8 (13.3–38.3)
Number of children responsible for at home	400			352			111		
None	275	68.8	71.6 (66.0–77.1)	304	86.4	87.5 (83.5–91.5)	100	90.1	87.2 (79.7–94.7)
One or more	125	31.3	28.4 (22.9–34.0)	48	13.6	12.5 (8.5–16.5)	11	9.9	12.8 (5.3–20.3)
Have cut foreskin	400			352			111		
Yes	240	60.0	59.5 (53.4–65.5)	291	82.7	83.4 (78.9–87.9)	85	76.6	73.4 (63.8–83.0)
No	160	40.0	40.5 (34.5–46.6)	61	17.3	16.6 (12.1–21.1)	26	23.4	26.6 (17.0–36.2)
Screened positive for depression	400			352			111		
Yes	151	37.8	38.9 (32.9–44.9)	191	54.3	54.5 (48.3–60.6)	69	62.2	67.5 (57.8–77.2)
No	249	62.2	61.1 (55.1–67.1)	161	45.7	45.5 (39.4–51.7)	42	37.8	32.5 (22.8–42.2)
Disclosed sexual behaviours to family or friends (non-MSM)	400						111		
Yes	160	40.0	38.1 (32.2–44.0)	140	39.8	36.0 (30.2–41.8)	44	39.6	35.3 (25.3–45.3)
No	240	60.0	61.9 (56.0–67.8)	212	60.2	64.0 (58.2–69.8)	67	60.4	64.7 (54.7–74.7)
Ashamed to be MSM or TGW	395			335			106		
Yes	120	30.4	32.6 (26.8–38.4)	92	27.5	29.8 (24.0–35.7)	32	30.2	28.2 (18.6–37.9)
No	275	69.6	67.4 (61.6–73.2)	243	72.5	70.2 (64.3–76.0)	74	69.8	71.8 (62.1–81.4)
Hide sexual behavior or gender identity from healthcare worker	359			209			60		
Yes	173	48.2	48.0 (41.5–54.5)	89	42.6	44.9 (36.8–53.0)	20	33.3	39.5 (25.2–53.7)
No	186	51.8	52.0 (45.5–58.5)	120	57.4	55.1 (47.0–63.2)	40	66.7	60.5 (46.3–74.8)
Can rely on other MSM and TGW to accompany them to doctor or hospital	373			334			103		
Yes	144	38.6	35.9 (29.8–41.9)	204	61.1	58.6 (52.3–64.9)	62	60.2	61.2 (50.5–71.9)
No	229	61.4	64.1 (58.1–70.2)	130	38.9	41.4 (35.1–47.7)	41	39.8	38.8 (28.1–49.5)
Physical violence, last 12 months	360			344			111		
Yes	109	30.3	28.5 (22.8–34.3)	77	22.4	21.5 (16.5–26.4)	19	17.1	13.6 (6.7–20.6)
No	251	69.7	71.5 (65.7–77.2)	267	77.6	78.5 (73.6–83.5)	92	82.9	86.4 (79.4–93.3)
Sexual violence, last 12 months	399			351			110		
Yes	40	10.0	9.9 (6.2–13.6)	23	6.6	5.4 (2.7–8.2)	8	7.3	8.0 (2.0–14.0)
No	359	90.0	90.1 (86.4–93.8)	328	93.4	94.6 (91.8–97.3)	102	92.7	92.0 (86.0–98.0)

While 38.9% screened positive for depression in Port Moresby, 54.5% did so in Lae and 67.5% in Mt. Hagen. Less than 40% of MSM and TGW in all three cities had disclosed to family or friends that they have sex with men and slightly less than one-third were ashamed to be MSM or TGW. Hiding sexual behavior or gender identity from healthcare workers was practiced by 48.0% in Port Moresby, 44.9% in Lae, and 39.5% in Mt. Hagen. A smaller proportion of MSM and TGW in Port Moresby (35.9%) could rely on peers to accompany them to the doctor or hospital than in Lae (58.6%) and Mt. Hagen (61.2%). Exposure to physical violence in the last 12 months was substantially higher in Port Moresby (28.5% compared to 13.6% in Mt. Hagen); whereas exposure to sexual violence in the last 12 months was comparable across all three cities (9.9% in Port Moresby, 5.4% in Lae, and 8.0% in Mt. Hagen).

Upwards of 45% of MSM and TGW first had sex with another MSM or TGW before age 20 (Table 2). MSM and TGW had at least three male sex partners in the last six months, with over half having 10 or more. The internet and mobile applications were used to find sex partners to a greater extent (42.6%) in Mt. Hagen, where MSM and TGW are most hidden, than in the other study cities. Concurrent partnerships were engaged in by 73.2% of MSM and TGW in Port Moresby, 77.9% in Lae, and 75.9% in Mt. Hagen, and approximately three-quarters had sex with a woman in the last six months in all three cities. More MSM and TGW sold sex in the last six months in Port Moresby than Lae and Mt. Hagen (51.6% versus 38.5 and 33.0%, respectively), whereas payment to a man or TGW for sex in the last six months was similar across all cities (9.3, 12.9, and 11.7%, respectively). Condom use at last anal sex with a male or transgender woman was limited at 26.9% in Port Moresby, 26.3% in Lae, and 32.5% in Mt. Hagen.

**Table 2:** Sexual behaviors of men who have sex with men and transgender women in Port Moresby, Lae and Mt. Hagen

	PORT MORESBY			LAE			MOUNT HAGEN		
	Valid	Sample proportion (unweighted)	Population proportion (weighted)	Valid	Sample proportion (unweighted)	Population proportion (weighted)	Valid	Sample proportion (unweighted)	Population proportion (weighted)
	*N* = 400	%	% (95% CI)	*N* = 352	%	% (95% CI)	*N* = 111	%	% (95% CI)
Age first had anal sex with a man or TG (years)	376			332			108		
10–14	33	8.8	7.1 (4.2–10.0)	28	8.4	7.5 (4.3–10.7)	2	1.9	3.0 (0.0–7.1)
15–19	143	38.0	37.7 (31.6–43.9)	123	37.0	40.7 (34.4–47.1)	54	50.0	47.9 (37.1–58.7)
20–24	108	28.7	30.3 (24.4–36.2)	103	31.0	29.4 (23.7–35.1)	36	33.3	32.9 (22.7–43.2)
25–29	65	17.3	17.6 (12.7–22.5)	45	13.6	14.5 (9.8–19.0)	9	8.3	10.3 (3.5–17.1)
30 or older	27	7.2	7.3 (4.0–10.7)	33	9.9	7.9 (4.8–11.0)	7	6.5	5.9 (1.1–10.8)
Total number of male or TGW partners in the last 6 months	400			352			111		
1–2 partners	0	0.0	0.0	0	0.0	0.0	0	0.0	0.0
3–4 partners	35	8.8	10.7 (6.6–14.8)	3	0.9	0.7 (0.0–1.5)	1	0.9	1.4 (0.0–4.3)
5–9 partners	132	33.0	32.1 (26.5–37.8)	119	33.8	32.1 (26.4–37.8)	34	30.6	26.1 (17.0–35.1)
10 or more partners	233	58.3	57.2 (51.1–63.3)	230	65.3	67.2 (61.4–72.9)	76	68.5	72.5 (63.2–81.8)
Used internet or mobile apps to meet partners, last 6 months	399			351			111		
Yes	85	21.3	23.2 (17.9–28.4)	98	27.9	24.3 (19.1–29.4)	45	40.5	42.6 (32.0–53.2)
No	314	78.7	76.8 (71.6–82.1)	253	72.1	75.7 (70.6–80.9)	66	59.5	57.4 (46.8–68.0)
Partner concurrency in the last 6 months	197			187			67		
Yes	157	79.7	73.2 (64.8–81.6)	150	80.2	77.9 (70.5–85.2)	53	79.1	75.9 (63.7–88.2)
No	40	20.3	26.8 (18.4–35.2)	37	19.8	22.1 (14.8–29.5)	14	20.9	24.1 (11.8–36.3)
Had vaginal/anal sex with a woman in the last 6 months	400			352			111		
Yes	294	73.5	72.8 (67.4–78.3)	302	85.8	86.8 (82.8–90.9)	95	85.6	84.1 (76.2–91.9)
No	106	26.5	27.2 (21.7–32.6)	50	14.2	13.2 (9.1–17.2)	16	14.4	15.9 (8.1–23.8)
Had a main male partner, last 6 months	378			339			103		
Yes	152	40.2	39.4 (33.2–45.5)	72	21.2	20.0 (15.0–24.9)	20	19.4	22.7 (13.1–32.3)
No	226	59.8	60.6 (54.5–66.8)	267	78.8	80.0 (75.1–85.0)	83	80.6	77.3 (67.7–86.9)
Condom use with main male partner(s), last 6 months	152			71			20		
Always	31	20.4	20.4 (12.4–28.4)	17	23.9	16.8 (7.1–26.5)	6	30.0	32.6 (7.8–57.4)
Sometimes	76	50.0	50.3 (40.2–60.3)	32	45.1	49.8 (35.5–64.1)	5	25.0	16.8 (0.0–34.3)
Never	45	29.6	29.3 (20.2–38.5)	22	31.0	33.4 (19.6–47.2)	9	45.0	50.6 (24.5–76.8)
Usual sexual position with main male partner	151			71			20		
Receptive	36	23.8	23.8 (15.4–32.2)	21	29.6	25.5 (13.6–37.4)	3	15.0	10.7 (0.0–25.9)
Insertive	101	66.9	65.9 (56.3–75.4)	46	64.8	68.1 (55.2–81.1)	14	70.0	68.6 (43.9–93.3)
Both	14	9.3	10.3 (3.9–16.7)	4	5.6	6.3 (0.0–13.3)	3	15.0	20.7 (0.0–43.1)
Had a casual male partner, last 6 months	378			339			103		
Yes	216	57.1	54.8 (48.4–61.1)	236	69.6	66.9 (60.9–72.9)	68	66.0	66.4 (55.9–76.8)
No	162	42.9	45.2 (38.9–51.6)	103	30.4	33.1 (27.1–39.1)	35	34.0	33.6 (23.2–44.1)
Condom use with casual male partner(s), last 6 months	216			236			68		
Always	41	19.0	18.3 (11.7–24.9)	55	23.3	24.5 (17.9–31.2)	21	30.9	31.7 (18.8–44.5)
Sometimes	80	37.0	37.8 (29.7–46.0)	99	41.9	40.8 (33.3–48.3)	11	16.2	11.8 (3.8–19.8)
Never	95	44.0	43.9 (35.4–52.3)	82	34.7	34.7 (27.4–41.9)	36	52.9	56.5 (43.0–70.1)
Sold sex in the last 6 months	378			339			103		
Yes	195	51.6	51.6 (45.3–58.0)	143	42.2	38.5 (32.4–44.5)	37	35.9	33.0 (22.7–43.4)
No	183	48.4	48.4 (42.0–54.7)	196	57.8	61.5 (55.5–67.6)	66	64.1	67.0 (56.6–77.3)
Paid a man or TGW for sex in the last 6 months	378			339			103		
Yes	43	11.4	9.3 (5.9–12.7)	42	12.4	12.9 (8.6–17.1)	13	12.6	11.7 (4.6–18.8)
No	335	88.6	90.7 (87.3–94.1)	297	87.6	87.1 (82.9–91.4)	90	87.4	88.3 (81.2–95.4)
Condom use at last anal sex with a man or TGW	398			352			101		
Yes	109	27.4	26.9 (21.5–32.4)	92	26.1	26.3 (20.9–31.8)	33	32.7	32.5 (22.1–43.0)
No	289	72.6	73.1 (67.6–78.5)	260	73.9	73.7 (68.2–79.1)	68	67.3	67.5 (57.0–77.9)

Comprehensive knowledge of HIV was low among MSM and TGW at 37.6% in Port Moresby, 43.8% in Lae, and 48.3% in Mt. Hagen (Table 3). Between one-quarter and half of MSM and TGW had never interacted with a peer outreach worker. While 64.4% of MSM and TGW in Port Moresby received free condoms in the last 12 months, 42.7% had in Mt. Hagen. Experiences of STD symptoms in the last 12 months were common at 27.8% in Port Moresby, 31.5% in Lae, and 19.3% in Mt. Hagen.

**Table 3:** HIV knowledge, uptake of HIV services among, and HIV and syphilis prevalence men who have sex with men and transgender women in Port Moresby, Lae and Mt. Hagen

	PORT MORESBY			LAE			MOUNT HAGEN		
	Valid	Sample proportion (unweighted)	Population proportion (weighted)	Valid	Sample proportion (unweighted)	Population proportion (weighted)	Valid	Sample proportion (unweighted)	Population proportion (weighted)
	*N* = 400	%	% (95% CI)	*N* = 352	%	% (95% CI)	*N* = 111	%	% (95% CI)
Comprehensive knowledge of HIV	400			352			111		
Yes	153	38.3	37.6 (31.6–43.6)	163	46.3	43.8 (37.7–49.9)	51	45.9	48.3 (37.6–58.9)
No	247	61.8	62.4 (56.4–68.4)	189	53.7	56.2 (50.1–62.3)	60	54.0	51.7 (41.1–62.4)
Last contact with peer outreach	394			341			107		
Never	124	31.5	34.4 (28.5–40.4)	84	24.6	25.9 (20.4–31.5)	53	49.5	51.1 (40.3–62.0)
≤3 month	69	17.5	15.2 (10.9–19.5)	49	14.4	10.7 (7.2–14.3)	12	11.2	10.9 (4.3–17.4)
4–12 months	152	38.6	38.7 (32.7–44.8)	159	46.6	44.3 (38.1–50.5)	33	30.8	26.8 (17.4–36.1)
More than 12 months ago	49	12.4	11.6 (7.6–15.6)	49	14.4	19.1 (13.8–24.3)	9	8.4	11.3 (3.9–18.6)
Given free condoms, last 12 months	397			350			110		
Yes	271	68.3	64.4 (58.4–70.4)	188	53.7	48.1 (41.9–54.3)	52	47.3	42.7 (32.2–53.2)
No	126	31.7	35.6 (29.6–41.6)	162	46.3	51.9 (45.7–58.1)	58	52.7	57.3 (46.8–67.8)
Experienced at least one self-reported STD symptom in the last 12 months	400			352			111		
Yes	111	27.8	27.8 (22.3–33.3)	108	30.7	31.5 (25.8–37.2)	28	25.2	19.3 (11.4–27.1)
No	289	72.3	72.2 (66.7–77.7)	244	69.3	68.5 (62.8–74.2)	83	74.8	80.7 (72.9–88.6)
Ever tested for HIV	400			352			111		
Yes	162	40.5	41.8 (35.7–47.8)	127	36.1	32.1 (26.4–37.8)	29	26.1	28.7 (19.0–38.5)
No	238	59.5	58.2 (52.2–64.3)	225	63.9	67.9 (62.2–73.6)	82	73.9	71.3 (61.5–81.0)
Time since last HIV test	162			127			29		
In the last 6 months	72	44.4	42.4 (32.9–52.0)	43	33.9	37.4 (27.0–47.8)	15	51.7	47.8 (26.6–69.0)
6–12 months ago	37	22.8	21.6 (13.8–29.5)	30	23.6	21.2 (12.9–29.5)	8	27.6	31.3 (11.1–51.4)
More than 12 months ago	53	32.7	35.9 (26.6–45.2)	54	42.5	41.4 (30.9–51.9)	6	20.7	20.9 (3.6–38.3)
HIV Status	390			349			109		
Positive	30	7.7	8.5 (5.0–11.9)	22	6.3	6.9 (3.6–10.3)	2	1.8	1.3 (0.0–3.2)
Negative	360	92.3	91.5 (88.1–95.0)	327	93.7	93.1 (89.7–96.4)	107	98.2	98.7 (96.8–100.0)
Syphilis status	397			352			111		
Active infection	17	4.3	4.0 (1.7–6.4)	29	8.2	8.3 (4.8–11.7)	3	2.7	2.5 (0.0–5.7)
No active infection	380	95.7	96.0 (93.6–98.3)	323	91.8	91.7 (88.3–95.2)	108	97.3	97.5 (94.3–100.0)

Less than half of MSM and TGW had ever tested for HIV (41.8% in Port Moresby, 32.1% in Lae, and 28.7% in Mt. Hagen) and of those who had, over half in each city tested more than six months ago. Survey-related testing found HIV prevalence among MSM and TGW was 8.5% (95% CI: 5.0–11.9) in Port Moresby and 6.9% (95% CI: 3.6–10.3) in Lae, and 1.8% among survey participants in Mt. Hagen.^1^ Prevalence of active syphilis was 4.0, 8.5, and 2.5%, respectively, in the three cities. Prevalence of lifetime syphilis infection was 10.1, 21.1, and 8.3%, respectively (data not shown).

Among MSM and TGW in Port Moresby, correlates of HIV in multivariate analysis included not having sex with a woman in the last 6 months (adjusted odds ratio (aOR): 4.5, 95% CI: 1.6–12.6), having self-reported uncut foreskin (aOR: 6.5, 95% CI: 1.9–22.3), and reporting at least one symptom of an STD in the last 12 months (aOR: 6.0, 95% CI: 2.2–16.5) (Table 4). In Lae, correlates of HIV included earning income in the formal sector (versus informal sector) (aOR: 4.7, 95% CI: 1.2–18.3), being unable to rely on other MSM or TGW to accompany them to healthcare services (aOR: 8.5, 95% CI: 2.4–29.9), not having sex with a woman in the last six months (aOR: 7.7, 95% CI: 2.4–25.5), and experiencing at least one symptom of an STD in the last 12 months (aOR: 4.2, 95% CI: 1.4–13.1) (Table 5).

**Table 4:** Predictors of being HIV-positive among men who have sex with men and transgender women in Port Moresby

	N	Bivariate OR (95% CI)	*p*-value	Multivariable aOR (95% CI)	*p*-value
Age (years)	390		0.9606		
12–24		Ref			
25–29		1.02 (0.32–3.26)			
30+		1.15 (0.41–3.23)			
Gender identity	390		< 0.0001		
Male		Ref			
TG		9.61 (3.64–25.34)			
Education	390				
No formal education		Ref			
Primary		4.34 (1.06–17.84)			
High school or higher		2.18 (0.80–5.92)			
Main source of income	370				
Formal		2.03 (0.65–6.31)			
Informal		Ref			
Unemployed		1.10 (0.34–3.55)			
Marital status	390				
Never married		Ref			
Married		0.53 (0.13–2.14)			
Divorced, separated, or widowed		0.70 (0.18–2.71)			
Disclosed sexual behaviours to family or friends (non-MSM)	390				
Yes		Ref			
No		0.37 (0.15–0.92)			
Used internet or mobile apps to meet partners, last 6 months	389		0.0007		
Yes		4.85 (1.95–12.04)			
No		Ref			
Had vaginal/anal sex with a woman in the last 6 months	390		0.0057		0.0544
Yes		Ref		Ref	
No		7.88 (2.96–21.03)		4.49 (1.60–12.60)	
Have cut foreskin	390		0.0002		0.0143
Yes		Ref		Ref	
No		10.54 (3.06–36.36)		6.49 (1.89–22.25)	
Experienced at least one self-reported STD symptom in the last 12 months	390		0.0002		0.0179
Yes		6.02 (2.33–15.55)		5.95 (2.15–16.46)	
No		Ref		Ref	
Last contact with peer outreach	384		0.0399		
Never		0.41 (0.10–1.63)			
< 3 months		2.64 (0.92–7.55)			
4–12 months		Ref			
More than 12 months ago		0.90 (0.16–4.93)

**Table 5:** Predictors of being HIV-positive among men who have sex with men and transgender women in Lae

	N	Bivariate OR (95% CI)	*p*-value	Multivariable aOR (95% CI)	*p*-value
Age (years)	342		0.1707		
12–24		Ref			
25–29		2.34 (0.61–8.94)			
30+		3.14 (0.93–10.57)			
Gender identity	349		0.8043		
Male		Ref			
TG		1.19 (0.30–4.78)			
Education	349		0.6174		
No formal education		Ref			
Primary		1.95 (0.28–13.64)			
High school or higher		2.46 (0.39–15.41)			
Main source of income	337		0.0405		0.0312
Informal sector		Ref		Ref	
Formal sector		5.08 (1.33–19.39)		4.66 (1.19–18.28)	
Unemployed		1.85 (0.42–8.09)		0.99 (0.20–4.89)	
Marital status	349		0.1336		
Never married		Ref			
Married		1.90 (0.52–6.94)			
Divorced, separated, or widowed		0.18 (0.02–1.45)			
Have cut foreskin	352		0.6639		
Yes		Ref			
No		1.30 (0.39–4.30)			
Can rely on other MSM/TGW to accompany them to doctor or hospital	332		0.0050		0.0010
Yes		Ref		Ref	
No		4.57 (1.59–13.16)		8.47 (2.40–29.90)	
Had vaginal/anal sex with a woman in the last 6 months	349		0. 0042		0.0008
Yes		Ref		Ref	
No		7.88 (2.96–21.03)		7.74 (2.35–25.53)	
Experienced at least one self-reported STD symptom in the last 12 months	349		0.0310		0.0131
Yes		3.18 (1.11–9.11)		4.21 (1.36–13.09)	
No		Ref		Ref	
Last contact with peer outreach	338		0.0432		
Never		0.52 (0.10–2.84)			
< 3 months		0.33 (0.08–1.38)			
4–12 months		Ref			
More than 12 months ago		2.52 (0.76–8.41)			

## Discussion

Our survey is the first biobehavioral HIV survey of MSM and TGW in PNG that includes both sex workers and non-sex workers. The large proportions of MSM and TGW with concurrent sexual partnerships or who sell sex, combined with the low testing coverage and condom use at last anal sex with a male or transgender woman, suggests substantial potential for the spread of HIV among these populations and their female sexual partners.

The different correlates of HIV in Port Moresby and Lae highlight the importance of conducting surveys in multiple locations and most importantly using such data to develop locally appropriate interventions even for the same population within a country. Having an uncut foreskin is associated with greater risk of HIV in Port Moresby but there is no association in Lae. In contrast, not being able to rely on peers for accompaniment to a clinic or hospital is not associated with HIV in Port Moresby but is associated with increased risk of the disease in Lae. This may be because there are fewer HIV and sexual health services for MSM and TGW in Lae than in Port Moresby, and the prospect of accessing services alone at facilities with staff not trained to work with MSM and TGW may prove daunting. Employment in the formal sector was also associated with HIV in Lae but not in Port Moresby, suggesting the need for greater targeting of HIV prevention and testing service in Lae compared to Port Moresby. In both cities, not having sex with a woman in the last six months was associated with HIV infection. While it is important to access all MSM and TGW for HIV services, given the low engagement of MSM and TGW in PNG with outreach workers and HIV testing, prioritization of limited HIV resources to MSM and TGW who do not have sex with women could reach more people as these individuals are often most accessible to KP services.

A high prevalence of sexually transmitted infections has been well documented in PNG, and we similarly documented a high prevalence of self-reported STD symptoms among MSM and TGW [19, 20]. Though active syphilis infection was not associated with HIV, in both Port Moresby and Lae, HIV was associated with having at least one STD symptom in the last 12 months. As STI can contribute to both the transmission and acquisition of HIV, those seeking treatment for STD symptoms should routinely be offered HIV testing [21, 22].

The lower proportion of MSM and TGW traveling outside of Port Moresby compared to other cities is likely due to the fact that Port Moresby is not connected to the rest of the country by road, whereas Lae and Mt. Hagen are on the main highway. This may also lend itself to preventing the spread of HIV from the capital to Lae and Mt. Hagen.

As the use of the internet and mobile applications to meet sexual partners has rapidly expanded in PNG and will likely continue to do so, these media platforms may be an effective way of reaching MSM and TGW with information about HIV and services. The relative recency (last 12 months) of interaction of MSM and TGW in Port Moresby and Lae with peer outreach workers suggests the potential for community-based HIV testing or provider-assisted self-testing.

Our findings are limited by the cross-sectional nature of our study and the low sample size in Mt. Hagen which we believe is due to the particularly heteronormative culture of this highlands city. Given that the lack of legal protections for MSM and TGW (which do not exist in PNG) are associated with higher HIV prevalence in a global meta-analysis and that HIV prevalence in the general population is highest in Mt. Hagen, we suspect that our finding of 1.8% prevalence among survey participants is an underestimate [23]. As interview data were self-reported in face-to-face interviews, there may be some response bias. The use of audio-computer assisted self-interviews rather than face-to-face interviews may have helped decrease this bias [24].

## Conclusions

Our survey was able to reach MSM and TGW that were not engaged in HIV services, revealing that MSM and TGW will access HIV services provided in a safe, affirming environment, providing further evidence to guide service providers and policy makers. As the first HIV biobehavioral survey in PNG among MSM and TGW, this study reveals the substantial vulnerabilities and HIV risks of these populations in PNG and their limited access to and uptake of routine HIV services. Without action, HIV prevalence stands to increase among MSM and TGW in PNG. Addressing the HIV epidemic in PNG will require continued and expanded engagement with MSM and TGW across the country, respecting and responding to their different needs and vulnerabilities, and moving beyond a once size fits all approach to these population in PNG.

## Additional files

Additional file 1: FSW Questionnaire, FSW Questionnaire, Quantitative interview questionnaire used with FSW participants. (DOCX 100 kb)

Additional file 2: MSM TGW Questionnaire, MSM/TGW questionnaire, Quantitative interview questionnaire used with MSM/TGW participants. (DOCX 112 kb)

## Abbreviations

aOR: Adjusted Odds Ratio
BBS: Biobehavioral survey
CI: Confidence Interval
MSM: Men who have sex with men
OR: Odds Ratio
PHQ-2: Patient Health Questionnaire-2
PNG: Papua New Guinea
RDS: Respondent-driven sampling
RDS-A: Respondent-Driven Sampling-Analyst
STD: Sexually transmitted disease
TGW: Transgender women

## Footnotes

1 Prevalence in Port Moresby and Lae is weighted and represents all MSM and TGW. Given the small sample size in Mt. Hagen, we report only on prevalence among survey participants.
