# Supplementary material for: One size does not fit all: HIV prevalence and correlates of risk for men who have sex with men, transgender women in multiple cities in Papua New Guinea
Source: BMC Public Health. 2019 May 22;19:623. doi: 10.1186/s12889-019-6942-7 (PMC6532262; doi:10.1186/s12889-019-6942-7)
Supplement: Supplementary file 1 — FSW Questionnaire, FSW Questionnaire, Quantitative interview questionnaire used with FSW participants. (DOCX 100 kb) [file 12889_2019_6942_MOESM1_ESM.docx]

**FSW Questionnaire**

READ TO THE PARTICIPANT:

Thank you for participating in the survey. Please remember that your answers to this survey are completely confidential, your name will not be attached to any of your answers, and no one other than me will know your answers. Your answers will be coded so we will not know who they belong to. So please answer as honestly as possible.

I would like to begin the interview by asking some questions about you.

| **N/A** | **VARIABLE:** |  | **Response options** | **SKIPS** | **COMMENTS** |
| --- | --- | --- | --- | --- | --- |
|  |  | **DEMOGRAPHICS** |  |  |  |
|  | DEAGENUM | How old were you at your last birthday? | AGE IN COMPLETED YEARS: [__\|__]  MIN: 12  MAX: CURRENT YEAR-DEAGEY  DON’T KNOW 97  REFUSE TO ANSWER 98 |  |  |
|  | DEREADWR | Can you read and write? | CANNOT READ AND WRITE 1  CAN READ ONLY 2  CAN READ AND WRITE 3  DON’T KNOW 7  REFUSE TO ANSWER 8 |  |  |
|  | DEEDEVER | Have you ever attended school? | YES 1  NO 2  DON’T KNOW 7  REFUSE TO ANSWER 8 | ‘2’,’7’ OR ‘8’🡪 SKIP TO DEMARSTA |  |
|  | DEEDHIGH | What is the highest level/grade/year of school you completed: | PRIMARY 1  SECONDARY 2  HIGHER 3  DON’T KNOW 7  REFUSE TO ANSWER 8 |  |  |
|  | DEMARSTA | What is your current marital status? | SINGLE, NEVER MARRIED 1  MARRIED AND HUSBAND HAS MULTIPLE WIVES (POLYGAMIST) 2  MARRIED AND I AM THE ONLY WIFE (MONOGAMOUS) 3  SEPARATED/DIVORCED 3  WIDOWED 4  DON’T KNOW 7  REFUSE TO ANSWER 8 |  |  |
|  | DELIVESX | Are you currently living with a sexual partner? | YES 1  NO 2  DON’T KNOW 7  REFUSE TO ANSWER 8 |  |  |
|  | DEREG | Do you sleep in the same place most nights? | YES 1  NO 2  DON’T KNOW 7  REFUSE TO ANSWER 8 |  |  |
|  | DECURLIV | In which neighborhood do you currently live? | Boroko 1  Gerehu 2  Hanubada 3  Gordons 4  Waigani 5  Murray Barracks 6  Morata 7  Garden Hill 8  Taurama 9  Erima 10  Koki 11  Downtown Moresby 12  Pari/ Kaugere / Kilakila 13  Porebada 14  Bomana 15  Konedobu 16  Pagha Hills 17  6 Mile 18  7 Mile 19  8 Mile 20  9 Mile 21  Along the Hiritana Higway 22  Along the Magi Highway 23  Other 24  (Specify)  DON’T KNOW 7  REFUSE TO ANSWER 8 |  |  |
|  | DERESIDE | How long have you been in Port Moresby/Lae/Hagen?  ENTER ‘0’ IF LESS THAN 1 YEAR. | # OF YEARS: [__\|__]  MIN: 0  MAX: 96  DON’T KNOW 97  REFUSE TO ANSWER 98 |  |  |
|  | DEAWAYT | In the last 6 months, how many times have you been away from home for one or more nights? By “home”, we mean the place you usually live. By “away from home”, we mean spending one or more nights away from the place you usually live. This could be even in the same province.  ENTER ‘0’ IF NONE. | # TIMES [__\|__\|__]  MIN: 0  MAX: 180  DON’T KNOW 997  REFUSE TO ANSWER 998 | ‘0’ OR ‘997’ OR ‘998’🡪 SKIP TO DEEMPSRC |  |
|  | DEAWAYM | In the last 6 months, have you been away from home for more than one month at a time? | YES 1  NO 2  DON’T KNOW 7  REFUSE TO ANSWER 8 |  |  |
|  | DEEMPSRC | What is your main source of income? | NO INCOME 1  RELY ON FAMILY AND FRIENDS 2  SELL SEX 3  STREET SALES 4  OTHER 6  DON’T KNOW 7  REFUSES TO ANSWER 8 |  |  |
|  | DEINCOME | How much money do you earn in a normal month? | AMOUNT_______  DON’T KNOW 9997  REFUSE TO ANSWER 9998 |  | Min and Max values of numeric field will be set as per local currency value. |
|  | DEchiliv | How many children do you have currently living with you that you are responsible for (including those you may have adopted or care for)?  ENTER ‘0’ IF NONE. | NUMBER [__\|__]  MIN: 0  MAX: 96  DON’T KNOW 97  REFUSE TO ANSWER 98 |  |  |
|  | DESUPP | How many children are you responsible for who do not live with you?  ENTER ‘0’ IF NONE. | NUMBER [__\|__]  MIN: 0  MAX: 96  DON’T KNOW 97  REFUSE TO ANSWER 98 |  |  |
|  | DELANG | What language do you speak most often? | ENGLISH 1  PIGIN 2  MOTU 3  OTHER 6  DON’T KNOW 7  REFUSES TO ANSWER 8 |  |  |
|  | DEETHNIC | To which [ethnic group/race/tribe] do you belong? | ETHNICITY/RACE A 1  ETHNICITY/RACE B 2  ETHNICITY/RACE C 3  MIXED ETHNICITY/RACE 4  OTHER 6  DON’T KNOW 7  REFUSES TO ANSWER 8 |  | Fill in ethnicities/ tribes during pre-test |
|  | DERELIG | What religion are you? | CATHOLIC 1  ANGLICAN 2  UNITED CHURCH 3  SEVENTH DAY ADVENTIST 4  REVIVAL CHURCH PNG 5  FOUR SQUARE CHURCH 6  ASSEMBLY OF GOD 7  LUTHERAN 8  BAHAI 9  MUSLIM 10  OTHER 66  DON’T KNOW 97  REFUSES TO ANSWER 98 |  |  |
|  | DERGO | How many times did you go to church or a place of worship in the last month? | VALUE ____  DON’T KNOW 9997  REFUSE TO ANSWER 9998 |  |  |
|  |  | *Next we will ask you some questions on depression. Over the last 2 weeks, how often have you been bothered by any of the following problems:* | | | |
|  | DPINTRST | Little interest or pleasure in doing things | NOT AT ALL 0  SEVERAL DAYS 1  MORE THAN HALF THE DAYS 2  NEARLY EVERY DAY 3  REFUSE TO ANSWER 8 |  |  |
|  | DPDOWN | Feeling down, depressed, or hopeless | NOT AT ALL 0  SEVERAL DAYS 1  MORE THAN HALF THE DAYS 2  NEARLY EVERY DAY 3  REFUSE TO ANSWER 8 |  |  |
|  |  | **ALCOHOL** |  |  |  |
|  | ALFRQ | How often do you have a drink containing alcohol?  By a drink I mean, 1 SP or other beer, 1 shot of whiskey,… | NEVER 0  MONTHLY OR LESS 1  2-4 TIMES A MONTH 2  2-3 TIMES A WEEK 3  4 OR MORE TIMES A WEEK 4  REFUSE TO ANSWER 8 | ‘0’🡪SKIP TO END OF SECTION | audit-c/AUDIT  RISK |
|  | ALDAY | How many drinks containing alcohol do you have on a typical day when you are drinking? | 1 OR 2 0  3 OR 4 1  5 OR 6 2  7-9 3  10 OR MORE 4  REFUSE TO ANSWER 8 |  | audit-c/AUDIT  RISK |
|  | ALBNGE | On any one occasion, how often do you have six or more alcoholic drinks? | NEVER 0  LESS THAN MONTHLY 1  MONTHLY 2  WEEKLY 3  DAILY OR ALMOST DAILY 4  REFUSE TO ANSWER 8 | IF ALDAY=’0’ and ALBINGE = ‘0’🡪Skip to END OF SECTION | Audit-c/audit  RISK |
|  | ALCTRL | During the last year, how often have you found that you were not able to stop drinking once you had started? | NEVER 0  LESS THAN MONTHLY 1  MONTHLY 2  WEEKLY 3  DAILY OR ALMOST DAILY 4  REFUSE TO ANSWER 8 |  | audit  RISK |
|  | ALFAIL | During the last year, how often have you failed to do what was normally expected of you because of drinking? | NEVER 0  LESS THAN MONTHLY 1  MONTHLY 2  WEEKLY 3  DAILY OR ALMOST DAILY 4  REFUSE TO ANSWER 8 |  | audit  RISK |
|  | ALMORN | During the last year, how often have you needed a first drink in the morning to get yourself going after a heavy drinking session? | NEVER 0  LESS THAN MONTHLY 1  MONTHLY 2  WEEKLY 3  DAILY OR ALMOST DAILY 4  REFUSE TO ANSWER 8 |  | audit  RISK |
|  | ALGUILT | During the last year, how often have you had a feeling of guilt or remorse after drinking? | NEVER 0  LESS THAN MONTHLY 1  MONTHLY 2  WEEKLY 3  DAILY OR ALMOST DAILY 4  REFUSE TO ANSWER 8 |  | audit  RISK |
|  | ALMEM | During the last year, how often have you been unable to remember what happened the night before because of your drinking? | NEVER 0  LESS THAN MONTHLY 1  MONTHLY 2  WEEKLY 3  DAILY OR ALMOST DAILY 4  REFUSE TO ANSWER 8 |  | audit  RISK |
|  | ALINJur | Have you or someone else been injured because of your drinking? | NO 0  YES, BUT NOT IN THE LAST YEAR 1  YES, DURING THE LAST YEAR 3  REFUSE TO ANSWER 8 |  | audit  RISK |
|  | ALCNCRN | Has a relative, friend, doctor, or other health care worker been concerned about your drinking or suggested you cut down? | NO 0  YES, BUT NOT IN THE LAST YEAR 1  YES, DURING THE LAST YEAR 3  REFUSE TO ANSWER 8 |  | AUDIT  RISK |
| **NON-INJECTION DRUG USE** | | | | | |
|  | DU1MSG | **The next few questions are on the use of any drugs that you may have used without injecting. Such drugs may be smoked, inhaled or snorted. These include drugs like marijuana, crystal meth, cocaine, crack, ecstasy, heroin, or opium [insert drugs from local context].** |  |  |  |
|  | DUEVER | Have you ever smoked, drunk (dringim) or snorted a drug that was not prescribed by a health care worker?  crystal meth, cocaine, crack, ecstasy, heroin, or opium marijuana, | YES 1  NO 2  DON’T KNOW 7  REFUSE TO ANSWER 8 | ‘2’,’7’ or ‘8’ →SKIP TO END OF SECTION |  |
|  | DU6MO | In the last 6 months, have you smoked, inhaled or snorted marijuana, crystal, meth, cocaine, crack, ecstasy, heroin or opium? | YES 1  NO 2  DON’T KNOW 7  REFUSE TO ANSWER 8 | ‘2’,’7’ or ‘8’ →SKIP TO END OF SECTION | Drugs from local context will be used.  RISK |
|  |  | *The next set of questions is about injection drug use. This means injecting yourself with drugs that are not prescribed for you or having someone who isn't a health care provider inject you.* |  |  |  |
|  | IDEVER | Have you ever injected any drugs other than those prescribed for you? By injecting, I mean anytime you have used any illegal or illicit drugs to get high. | YES 1  NO 2  DON’T KNOW 7  REFUSED 8 | ‘2’, ‘7’ OR ‘8’ →SKIP TO END OF SECTION |  |
|  | ID6mos | In the last 6 months, have you injected any illicit or illegal drugs? | YES 1  NO 2  DON’T KNOW 7  REFUSED 8 | ‘2’, ‘7’ OR ‘8’🡪SKIP TO END OF SECTION |  |
|  | idNLOC | In the last 6 months when you injected, where did you get your needles/syringes from?  CHECK ALL THAT APPLY. | PHARMACY/CHEMIST/DRUG STORE/OTHER STORE A  DOCTOR’S OFFICE, CLINIC OR HOSPITAL, OTHER HEALTH AGENCY, OR HIV PREVENTION PROGRAM B  MARKET PLACE OR STREET VENDOR C  DRUG WORKER OR AGENCY/OUTREACH WORKER OR STREET UNIT D  SEX PARTNER, FRIEND, ACQUAINTANCE, RELATIVE E  DRUG DEALER OR OTHER DRUG USERS F  NEEDLE/SYRINGE EXCHANGE PROGRAM G  OTHER X  DON’T KNOW Y  REFUSED Z |  |  |
|  | IDINJT | In the last 6 months, how often did you use needles that someone else had already injected with? | NEVER 1  RARELY 2  HALF OF THE TIME 3  MOST OF THE TIME 4  ALWAYS 5  DON’T KNOW 7  REFUSED 8 |  |  |
|  |  | **SOCIAL COHESION** |  |  |  |
|  |  | **The next several questions are about your social life and your relationships with other women and girls to sell and or exchange sex. Please mark if you strongly disagree, disagree, are neutral, agree, or strongly agree with the statements.** |  |  |  |
|  | SCDOC | You can count on other sex workers to accompany you to the doctor or hospital. | STRONGLY DISAGREE 1  DISAGREE 2  NEUTRAL 3  AGREE 4  STRONGLY AGREE 5  DON’T KNOW 9997  REFUSE TO ANSWER 9998 |  |  |
|  | SCVIOL | You can count on other sex workers to help you deal with a violent or difficult [client/partner/person in your life]. | STRONGLY DISAGREE 1  DISAGREE 2  NEUTRAL 3  AGREE 4  STRONGLY AGREE 5  DON’T KNOW 9997  REFUSE TO ANSWER 9998 |  |  |
|  | SCSUP | You can count on other sex workers to support your use of condoms. | STRONGLY DISAGREE 1  DISAGREE 2  NEUTRAL 3  AGREE 4  STRONGLY AGREE 5  DON’T KNOW 9997  REFUSE TO ANSWER 9998 |  |  |
|  |  | **The next several questions ask about things you may have done to help others. Please indicate if you have done any of the following in the past 12 months.** |  |  |  |
|  | SCPOLI | In the past 12 months, have you negotiated with or stood up against police in order to help a fellow woman or girl who sells or exchanges sex? | YES 1  NO, NOT PART OF SITUATOIN 2  NO, PART OF SITUATION BUT DID NOT SUPPORT 3  DON’T KNOW 7  REFUSE TO ANSWER 8 |  |  |
|  | SCMAD | In the past 12 months, have you negotiated with or stood up against a madam/broker/pimp in order to help a fellow woman or girl who sells or exchanges sex? | YES 1  NO 2  DON’T KNOW 7  REFUSE TO ANSWER 8 |  |  |
|  | SCPART | In the past 12 months, have you negotiated with or stood up against clients/any other sexual partner in order to help a fellow woman or girl who sells or exchanges sex? | YES 1  NO 2  DON’T KNOW 7  REFUSE TO ANSWER 8 |  |  |
|  |  | **SEXUAL HISTORY** |  |  |  |
|  | SUBSECTION ID: F  LI5MSG | **The next few questions are about your lifetime sexual history. This includes vaginal and anal sex. Vaginal sex is when the penis is inserted into the vagina. Anal sex is when a penis is inserted into the anus.** |  |  |  |
|  | LIFMANAL | Have you ever had anal sex? By anal sex I mean where a man inserts his penis into another person’s anus. | YES 1  NO 2  DON’T KNOW 7  REFUSE TO ANSWER 8 |  |  |
|  |  | **Please think back to the first time you had sex and provide answers to the next questions.** |  |  |  |
|  | LIFM1AGE | How old were you when you first had anal sex with a male partner? | YEARS [__\|__]  MAX: CURRENT AGE  DON’T KNOW 97  REFUSE TO ANSWER 98 |  |  |
|  | LIFmAGPA | Approximately how old was your male sex partner at the time you first had anal sex?  *PLEASE GIVE YOUR BEST GUESS* | MORE THAN 10 YEARS YOUNGER THAN ME 1  5-10 YEARS YOUNGER THAN ME 2  ABOUT THE SAME AGE 3  5-10 YEARS OLDER THAN ME 4  MORE THAN 10 YEARS OLDER THAN ME 5  DON’T KNOW 7  REFUSE TO ANSWER 8 |  |  |
|  | LIFMGET | Did your first male sex partner pay you or give you something in exchange for anal sex? | YES 1  NO 2  DON’T KNOW 7  REFUSE TO ANSWER 8 |  |  |
|  |  | The first time you had anal sex, was in because you wanted to or because you were forced? | WANTED TO 1  FORCED 2  DON’T KNOW 7  REFUSE TO ANSWER 8 |  |  |
|  |  | The first time you had anal sex, were you physically forced were you pressured into having sex through harassment, threats, or tricks? | PHYSICALLY FORCED 1  PRESSURED 2  DON’T KNOW 7  REFUSE TO ANSWER 8 |  |  |
|  | LIFMID | How would you describe the first man you had anal sex with? | BOYFRIEND/PARTNER 1  FRIEND/ACQUAINTANCE/COWORKER   2  BIOLOGICAL FATHER  FATHER FIGURE / UNCLE  OTHER MALE RELATIVE   3  STRANGER   4  ‘AUTHORITY FIGURE’ (GOVERNMENT OFFICIAL RELIGIOUS LEADER, TEACHER, EMPLOYER, MILITARY, POLICE, PRISON GUARD)   5  OTHER   6  DON’T KNOW 7  REFUSE TO ANSWER 8 |  |  |
|  |  | How old were you when you first had vaginal sex with a male partner? | YEARS [__\|__]  MAX: CURRENT AGE  DON’T KNOW 97  REFUSE TO ANSWER 98 |  |  |
|  | LIFmAGPA | Approximately how old was your male sex partner at the time you first had vaginal sex?  *PLEASE GIVE YOUR BEST GUESS* | MORE THAN 10 YEARS YOUNGER THAN ME 1  5-10 YEARS YOUNGER THAN ME 2  ABOUT THE SAME AGE 3  5-10 YEARS OLDER THAN ME 4  MORE THAN 10 YEARS OLDER THAN ME 5  DON’T KNOW 7  REFUSE TO ANSWER 8 |  |  |
|  | LIFMGET | Did your first male sex partner pay you or give you something in exchange for vaginal sex? | YES 1  NO 2  DON’T KNOW 7  REFUSE TO ANSWER 8 |  |  |
|  |  | The first time you had vaginal sex, was in because you wanted to or because you were forced? | WANTED TO 1  FORCED 2  DON’T KNOW 7  REFUSE TO ANSWER 8 |  |  |
|  |  | The first time you had vaginal sex, were you physically forced were you pressured into having sex through harassment, threats, or tricks? | PHYSICALLY FORCED 1  PRESSURED 2  DON’T KNOW 7  REFUSE TO ANSWER 8 |  |  |
|  | LIFMID | How would you describe the first man you had vaginal sex with? | BOYFRIEND/PARTNER 1  FRIEND/ACQUAINTANCE/COWORKER   2  BIOLOGICAL FATHER  FATHER FIGURE / UNCLE  OTHER MALE RELATIVE   3  STRANGER   4  ‘AUTHORITY FIGURE’ (GOVERNMENT OFFICIAL RELIGIOUS LEADER, TEACHER, EMPLOYER, MILITARY, POLICE, PRISON GUARD)   5  OTHER   6  DON’T KNOW 7  REFUSE TO ANSWER 8 | IF <>6 🡪SKIP TO END OF SECTION |  |
|  |  | **SEX WORK CHARACTERISTICS** |  |  |  |
|  |  | **Now we are going to ask you some questions about receiving money to have sex.** |  |  |  |
|  | SWAGE | How old were you when you first had sex with someone in exchange for money, gifts, goods or services? | YEARS: \|__\|__\|  MIN:1  MAX: CURRENT AGE  DON’T KNOW 97  REFUSE TO ANSWER 98 |  |  |
|  | SWReason | What is the main reason you started selling or exchanging sex for money, gifts, goods or services? | NEEDED MONEY TO HELP THE FAMILY 1  NEEDED MONEY TO PAY A DEBT 2  WAS FORCED 3  LIKE TO DO IT/PLEASURE/SELF-ESTEEM 4  FRIENDS/FAMILY WERE DOING IT 5  OTHER 6  DON’T KNOW 7  REFUSE TO ANSWER 8 |  |  |
|  | SWDURAT | For how many months or years have you been selling or exchanging sex for money, gifts, goods or services?  ENTER MONTHS IF LESS THAN 1 YEAR. | MONTHS: \|__\|__\|  YEARS: \|__\|__\|  MAX: CURRENT AGE  DON’T KNOW 97  REFUSE TO ANSWER 98 |  |  |
|  | SWLOC | In the last 6 months, in what *PART OF TOWN OR NEIGHBORHOOD* have you sold or exchanged sex for money, gifts, goods or services?  CHECK ALL THAT APPLY | Y N DK NR  BOROKO  GORDONS  WAIGANI  1 2 7 8  NEIGHBORHOOD B 7 8  NEIGHBORHOOD C 1 2 7 8  OTHER 6  DON’T KNOW 7  REFUSE TO ANSWER 8 |  |  |
|  | SWVENUE | Where do you usually meet or *find* clients? | BAR, CLUB, OR  LODGE /GUEST HOUSE 1  OTHER HOTEL 2  STREET OR PARK OR OTHER PUBLIC PLACES 3  PRIVATE HOME 4  PIMP / HOTEL MANAGER 5  PHONE 6  INTERNET/FACEBOOK 7  THROUGH FRIENDS 8  OTHER 9  DON’T KNOW 98  REFUSE TO ANSWER 99 |  |  |
|  | SWAGENT | Do you have someone who helps you meet clients? | YES 1  NO 2  DON’T KNOW 7  REFUSE TO ANSWER 8 |  |  |
|  | SWINCOME | Is selling or exchanging sex your main source of income/living? | YES 1  NO 2  DON’T KNOW 7  REFUSE TO ANSWER 8 |  |  |
|  | SWEARNA | What is the approximate amount you earn every time you have vaginal sex with a client? | VALUE [ __I__I__]  DON’T KNOW 9997  REFUSE TO ANSWER 9998 |  |  |
|  |  | What is the approximate amount you earn every time you have anal sex with a client? | VALUE [ __I__I__]  DON’T KNOW 9997  REFUSE TO ANSWER 9998 |  |  |
|  | SWEARNW | In the last week, how much money did you earn from selling sex? | VALUE: [__\|__\|__\|__]    DON’T KNOW 9997  REFUSE TO ANSWER 9998 |  |  |
|  | SWGOODS | In the last week, what goods or services did you receive from exchanging sex? | PHONE CARD 1  SCHOOL FEES 2  RENT 3  CLOTHES 4  TRANSPORT 5  FOOD 6  ALCOHOL 7  OTHER 8  DON’T KNOW 97  REFUSE TO ANSWER 98 |  |  |
|  | SWNOCON | In the last 6 months, how often were you able to negotiate condom use, when a client told you he did not want to use a condom? | NEVER 1  RARELY 2  SOMETIMES 3  FREQUENTLY 4  DON’T KNOW 7  REFUSE TO ANSWER 8 | ‘1’,’7’ OR ‘8’🡪SKIP TO SWSTIGMA |  |
|  | SWABUSE | In the last 6 months, has a client abused you? | YES 1  NO 2  DON’T KNOW 7  REFUSE TO ANSWER 8 |  |  |
|  | SWTHREAT | In the last 6 months, has a client ever threatened you? | YES 1  NO 2  DON’T KNOW 7  REFUSE TO ANSWER 8 |  |  |
|  | SWFORCE | In the last 6 months, has a client forced you to have sex? | YES 1  NO 2  DON’T KNOW 7  REFUSE TO ANSWER 8 |  |  |
|  | SWMOB | In the last 12 months, where else outside this town/city have you sold sex?  CHECK ALL THAT APPLY. | Y N DK NR  NOWHERE ELSE 1 2 7 8  OUTSIDE THE TOWN/CITY BUT INSIDE PROVINCE/DISTRICT 1 2 7 8  OUTISDE THE PROVINCE/DISTRICT BUT INSIDE COUNTRY 1 2 7 8  OUTSIDE COUNTRY 1 2 7 8 | SKIP TO END OF SECTION IF NOWHERE. |  |
|  | SWOUT | In the last 12 months, how often did you travel outside of this town/city to sell or exchange sex? | DAILY 1  WEEKLY 2  MONTHLY 3  A FEW TIMES A YEAR 4  DON’T KNOW 7  REFUSE TO ANSWER 8 |  |  |
|  | SWCLNR | In the last week, how many different men who are regular clients did you sell or exchange sex with?  A regular client is someone you have had sex with on more than one occasion. | [___\|___\|___\|___]  DON’T KNOW 9997  REFUSE TO ANSWER 9998 |  |  |
|  | SWCLNRC | The last time you had vaginal sex with a regular client, did you use a condom? | YES 1  NO 2  DON’T KNOW 7  REFUSE TO ANSWER 8 |  |  |
|  | SWCLNRC | The last time you had anal sex with a regular client, did you use a condom? | YES 1  NO 2  DON’T KNOW 7  REFUSE TO ANSWER 8 |  |  |
|  | SWCLNO | In the last week, how many different men who are one time clients did you sell or exchange sex with?  A one-time client is someone you have had sex with only once. | [___\|___\|___]  DON’T KNOW 9997  REFUSE TO ANSWER 9998 |  |  |
|  | SWCLNOC | The last time you had vaginal sex with a one-time client, did you use a condom? | YES 1  NO 2  DON’T KNOW 7  REFUSE TO ANSWER 8 |  |  |
|  | SWCLNOC | The last time you had anal sex with a one-time client, did you use a condom? | YES 1  NO 2  DON’T KNOW 7  REFUSE TO ANSWER 8 |  |  |
|  |  | **RECALL SEXUAL BEHAVIOR** |  |  |  |
|  |  | **This next section is about your sexual behavior in the last 6 months. The questions are about different sex partners, how many times you had sex, and condom use. *With sex we mean either vaginal sex or anal sex.* With vaginal sex we mean a penis enters a vagina. With anal sex we mean a penis enters a person’s anus (butt). We will ask you about different sex partner types: main sex partners, casual sex partners, people you pay money or give goods for sex, and people who pay you money or give you goods for sex. Each person you have had sex with in the last 6 months should be counted in one of these categories.** |  |  |  |
|  |  | **MALE PARTNERS** |  | (section) |  |
|  | LSACON | Did you use a condom the last time you had vaginal sex? | YES 1  NO 2  DON’T KNOW 7  REFUSE TO ANSWER 8 |  |  |
|  | LSACON | Did you use a condom the last time you had anal sex? | YES 1  NO 2  DON’T KNOW 7  REFUSE TO ANSWER 8 |  |  |
|  |  | **MAIN MALE SEX PARTNERS** |  | (SUBsection)  RISK |  |
|  | RCMAMNpa | As mentioned earlier, we will ask you about different types of sex partners. First we will ask you about any main male sex partners you may have had sex with in the last 6 months.  A main sex partner is someone you are committed to, for example your spouse, live-in sex partner, or boyfriend and you don’t receive money, gifts, goods, or services from in exchange for sex.  In the last 6 months, with how many different main male partners did you have sex? Type ‘0’ if none. | [___\|___\|___\|___]  DON’T KNOW 9997  REFUSE TO ANSWER 9998 | ‘0’,’9997’ OR ‘9998’🡪SKIPTO END OF SUBSECTION |  |
|  | RCMAMTYP | The last time you had sex with a main male partner, what type of sex did you have? | VAGINAL 1  ANAL 2  BOTH 3  DON’T KNOW 7  REFUSE TO ANSWER 8 |  |  |
|  | RCMAMNRC | The last time you had vaginal sex with a main male partner, did you use a condom? | YES 1  NO 2  DON’T KNOW 7  REFUSE TO ANSWER 8 |  |  |
|  | RCMAMNRC | The last time you had anal sex with a main male partner, did you use a condom? | YES 1  NO 2  DON’T KNOW 7  REFUSE TO ANSWER 8 |  |  |
|  | RCMAMNFQ | In the last 6 months, how often did you use condoms with your main male partners when having vaginal sex? | ALWAYS 1  MOST OF THE TIME 2  SOMETIMES 3  RARELY 4  NEVER 5  DON’T KNOW 7  REFUSE TO ANSWER 8 |  |  |
|  | RCMAMNFQ | In the last 6 months, how often did you use condoms with your main male partners when having anal sex? | ALWAYS 1  MOST OF THE TIME 2  SOMETIMES 3  RARELY 4  NEVER 5  DON’T KNOW 7  REFUSE TO ANSWER 8 |  |  |
|  | **cl** | **PARTNER TYPE: MALE CLIENTS** |  |  |  |
|  | rcmaclpa | Some people get money for sex. In the last 6 months, how many different men paid you money? Type ‘0’ if none. | [___\|___\|___\|___]  DON’T KNOW 9997  REFUSE TO ANSWER 9998 | ‘0’,’9997’ OR ‘9998’🡪 SKIP TO END OF SUBSECTIONEND OF SUBSECTION |  |
|  | RCMACLFQ | In the last 6 months, how often did you use condoms with men who gave you money for sex? | ALWAYS 1  MOST OF THE TIME 2  SOMETIMES 3  RARELY 4  NEVER 5  DON’T KNOW 7  REFUSE TO ANSWER 8 |  |  |
|  | rcmacla | The last time you had sex with any male who gave you money for sex, what type of sex did you have? | VAGINAL 1  ANAL 2  BOTH  OTHER - ORAL 3  DON’T KNOW 7  REFUSE TO ANSWER 8 |  |  |
|  | rcmaclac | The last time you had sex with a man who **gave you** money, did you use a condom? | YES 1  NO 2  DON’T KNOW 7  REFUSE TO ANSWER 8 |  |  |
|  | rcmaCTpa | Some people get gifts, goods or services in exchange for sex. In the last 6 months, how many different men **gave you** gifts, goods or services for sex? Type ‘0’ if none. | [___\|___\|___\|___]  DON’T KNOW 9997  REFUSE TO ANSWER 9998 | ‘0’,’9997’ OR ‘9998’🡪 SKIP TO END OF SUBSECTIONEND OF SUBSECTION |  |
|  | RCMACTFQ | In the last 6 months, how often did you use condoms with men who **gave you** gifts, goods or services for vaginal sex? | ALWAYS 1  MOST OF THE TIME 2  SOMETIMES 3  RARELY 4  NEVER 5  DON’T KNOW 7  REFUSE TO ANSWER 8 |  |  |
|  |  | In the last 6 months, how often did you use condoms with men who **gave you** gifts, goods or services for anal sex? | ALWAYS 1  MOST OF THE TIME 2  SOMETIMES 3  RARELY 4  NEVER 5  DON’T KNOW 7  REFUSE TO ANSWER 8 |  |  |
|  | rcmacTa | The last time you had sex with any male who gave you gifts, goods, or services for sex, what type of sex did you have? | VAGINAL 1  ANAL 2  BOTH 3  DON’T KNOW 7  REFUSE TO ANSWER 8 |  |  |
|  | rcmacTac | The last time you had anal sex with a man who **gave you** gifts, goods or services, did you use a condom? | YES 1  NO 2  DON’T KNOW 7  REFUSE TO ANSWER 8 |  |  |
|  |  | The last time you had vaginal sex with a man who **gave you** gifts, goods or services, did you use a condom? | YES 1  NO 2  DON’T KNOW 7  REFUSE TO ANSWER 8 |  |  |
|  | **CS** | **CASUAL MALE SEX PARTNERS** |  |  |  |
|  | rcmacspa | These next questions are about any casual male partners you may have had sex with in the last 6 months.  A casual male partner is a man you have sex with but don’t feel committed to. There is no payment or exchange of goods and services for sex with these partners.  In the last 6 months, with how many different casual male partners did you have sex? Type ‘0’ if none. | [___\|___\|___\|___]  DON’T KNOW 9997  REFUSE TO ANSWER 9998 | ‘0’,’9997’ OR ‘9998’🡪 SKIP TO END OF SUBSECTION |  |
|  | RCMACSFQ | In the last 6 months, how often did you use condoms with casual male partners when having vaginal sex? | ALWAYS 1  MOST OF THE TIME 2  SOMETIMES 3  RARELY 4  NEVER 5  DON’T KNOW 7  REFUSE TO ANSWER 8 |  |  |
|  |  | In the last 6 months, how often did you use condoms with casual male partners when having anal sex? | ALWAYS 1  MOST OF THE TIME 2  SOMETIMES 3  RARELY 4  NEVER 5  DON’T KNOW 7  REFUSE TO ANSWER 8 |  |  |
|  | rcmacsa | The last time you had sex with a casual male partner, what type of sex did you have? | VAGINAL 1  ANAL 2  BOTH 3  DON’T KNOW 7  REFUSE TO ANSWER 8 |  |  |
|  | rcmacsac | The last time you had vaginal sex with a casual male partner, did you use a condom? | YES 1  NO 2  DON’T KNOW 7  REFUSE TO ANSWER 8 |  |  |
|  |  | The last time you had anal sex with a casual male partner, did you use a condom? | YES 1  NO 2  DON’T KNOW 7  REFUSE TO ANSWER 8 |  |  |
|  |  | **CONDOM USE** |  |  |  |
|  |  | **Now we are going to ask you some questions about condoms and your experience using them** |  |  |  |
|  | COMLoc | Where can you get male condoms?  CHECK ALL THAT APPLY. | Y N DK NR  CLINIC/HOSPITAL 1 2 7 8  PHARMACY/SHOP 1 2 7 8  CHURCH/MOSQUE/TEMPLE 1 2 7 8  FRIEND/RELATIVE 1 2 7 8  COMMUNITY HEALTH WORKER 1 2 7 8  NGO 1 2 7 8  OTHER 1 2 7 8 |  |  |
|  | COFlOC | Where do you get female condoms?  CHECK ALL THAT APPLY. | Y N DK NR  CLINIC/HOSPITAL 1 2 7 8  PHARMACY/SHOP 1 2 7 8  CHURCH/MOSQUE/TEMPLE 1 2 7 8  FRIEND/RELATIVE 1 2 7 8  COMMUNITY HEALTH WORKER 1 2 7 8  NGO 1 2 7 8  OTHER 1 2 7 8 |  |  |
|  | COCARRY | Are you carrying any condoms with you right now? | YES 1  NO 2  DON’T KNOW 7  REFUSE TO ANSWER 8 |  |  |
|  | COASK | Could you ask your main sex partner to use a condom if you wanted? A main sex partner is someone you are committed to, for example your spouse, live-in sex partner, or boyfriend. There is no payment or exchange of goods or services for sex with these partners. | YES 1  NO 2  DON’T KNOW 7  REFUSE TO ANSWER 8 |  |  |
|  | COVUSE | Under what circumstances do you tend not to use condoms during vaginal sex?  With vaginal sex we mean a penis enters a vagina.  CHECK ALL THAT APPLY. | Y N DK NR  WHEN I’M DRUNK OR STONED 1 2 7 8  WHEN I AM AFRAID TO ASK MY PARTNER TO USE A CONDOM  WHEN MY PARTNER REFUSES 1 2 7 8  WHEN HAVING SEX WITH A REGULAR PARTNER 1 2 7 8  WHEN HAVING SEX WITH A NON-REGULAR PARTNER 1 2 7 8  WHEN THE PERSON DOES NOT EJACULATE INSIDE ME 1 2 7 8  WHEN I CANNOT FIND ONE 1 2 7 8  WHEN I CANNOT AFFORD TO BUY  A CONDOM 1 2 7 8  OTHER 1 2 7 8 | SKIP IF Participant did not have vaginal sex in the last 6 months. |  |
|  | COANO | Under what circumstances do you tend not to use condoms during anal sex? With anal sex we mean a penis enters a person’s anus.  CHECK ALL THAT APPLY. | Y N DK NR  WHEN I’M DRUNK OR STONED 1 2 7 8  WHEN I AM AFRAID TO ASK MY PARTNER TO USE A CONDOM  WHEN MY PARTNER REFUSES 1 2 7 8  WHEN HAVING SEX WITH A REGULAR PARTNER 1 2 7 8  WHEN HAVING SEX WITH A NON-REGULAR PARTNER 1 2 7 8  WHEN THE PERSON DOES NOT EJACULATE INSIDE ME 1 2 7 8  WHEN I CANNOT FIND ONE 1 2 7 8  WHEN I CANNOT AFFORD TO BUY  A CONDOM 1 2 7 8  OTHER 1 2 7 8 | SKIP IF Participant did not HAVE ANAL SEX IN THE LAST 6 MONTHS. |  |
|  | COfree | In the last 12 months, have you been given condoms for free? For example, through an outreach service, drop-in center or health clinic. | YES 1  NO 2  DON’T KNOW 7  REFUSE TO ANSWER 8 | SKIP TO COPREF |  |
|  | COSOURCE | From where did you receive these free condoms? | SOURCE A 1  SOURCE B 2  DON’T KNOW 7  REFUSE TO ANSWER 8 |  | Fill in possible sources during pre-test |
|  | copref | Do you prefer free condoms or branded condoms that you pay for? | FREE 1  BRANDED CONDOMS THAT I PAY FOR 2  NO PREFERENCE 3  DON’T KNOW 7  REFUSE TO ANSWER 8 |  |  |
|  | CONOGT | In the last 12 months, what are some reasons you couldn’t get condoms when you needed them?  CHECK ALL THAT APPLY. | Y N DK NR  CAN ALWAYS GET CONDOMS 1 2 7 8  COSTS TOO MUCH 1 2 7 8  NOT CONVENIENT 1 2 7 8  CLINIC DOES NOT PROVIDE THEM 1 2 7 8  EMBARRASSED TO GET CONDOMS 1 2 7 8  DO NOT KNOW WHERE TO GET CONDOMS 1 2 7 8  CONDOMS NOT AVAILABLE 1 2 7 8  OTHER 1 2 7 8 | IF ‘CAN ALWAYS GET CONDOMS’ NO OTHER CATEGORY CAN BE CHOSEN. |  |
|  | COINFO | In the last 12 months, have you received information on condom use and safer sex? For example, through an outreach service, drop-in center or health clinic. | YES 1  NO 2  DON’T KNOW 7  REFUSE TO ANSWER 8 |  |  |
|  | COINFOSO | From where did you receive this information? | SOURCE A 1  SOURCE B 2  DON’T KNOW 7  REFUSE TO ANSWER 8 |  | Fill in possible sources during pre-test |
|  |  | **LUBRICANT USE** |  |  |  |
|  |  | **Some people use lubricants during vaginal or anal sex. With vaginal sex we mean a penis enters a vagina. With anal sex we mean a penis enters a person’s anus. Lubricants make your penis or your partner’s penis more slippery and easier to insert into the vagina or anus. Lubricants also prevent the condom from breaking. Now we will ask you some questions about your use of lubricants in the last 6 months.** |  |  |  |
|  | LUDRY | Do you dry out your vagina? | YES 1  NO 2  DON’T KNOW 7  REFUSE TO ANSWER 8 |  | Adapt terminology of ‘dry sex’ to local context |
|  | LU6lub | In the last 6 months, have you used a lubricant during anal or vaginal sex? By lubricant I mean something that makes a penis slippery so it can more easily enter a vagina or anus. | YES 1  NO 2  DON’T KNOW 7  REFUSE TO ANSWER 8 | ‘2’,’7’ or ‘8’🡪 SKIP TO END OF SECTION |  |
|  | lutype | In the last 6 months, which lubricant did you use during vaginal or anal sex?  CHECK ALL THAT APPLY | Y N DK NR  WATER-BASED LUBE, KY JELLY, VENDOME 1 2 7 8  SALIVA 1 2 7 8  VASELINE, POMADE OR OTHER PETROLEUM JELLY PRODUCT 1 2 7 8  BODY LOTION, SHEA NUT BUTTER, OR BABY OIL 1 2 7 8  COOKING OIL, MAYONNAISE, BUTTER OR MARGARINE 1 2 7 8  OTHER 1 2 7 8 | SKIP TO LUAVAIL IF WATER-BASED LUBRICANT NOT SELECTED. | Modify sources during pre-test |
|  | lu3useva | In the last 6 months, how often did you use a lubricant during vaginal sex? | ALWAYS 1  SOMETIMES 2  NEVER 3  DON’T KNOW 7  REFUSE TO ANSWER 8 | SKIP IF NO VAGINAL SEX IN THE LAST 6 MONTHS FROM RECALL SEX MODULE. |  |
|  | luusean | In the last 6 months, how often did you use a lubricant during anal sex? | ALWAYS 1  SOMETIMES 2  NEVER 3  DON’T KNOW 7  REFUSE TO ANSWER 8 | ‘1’,’7’ OR ‘8’🡪SKIP TO LUCOST  SKIP IF NO ANAL SEX IN THE LAST 6 MONTHS FROM RECALL SEX MODULE. |  |
|  | lunotuse | In the last 6 months, what is the main reason you do not always use a lubricant during anal sex?  SELECT ONE ONLY. | CAN’T GET THEM EASILY/TOO EXPENSIVE 1  DO NOT LIKE LUBRICANTS 2  PARTNER DOESN’T LIKE THEM 3  I’VE NEVER HEARD OF IT 4  I’M ASHAMED/EMBARRASSED TO BUY IT BECAUSE IT IS ASSOCIATED WITH HOMOSEXUALS 5  DON’T KNOW ANYTHING ABOUT THEM 6  OTHER 7  DON’T KNOW 97  REFUSE TO ANSWER 98 | SKIP IF NO ANAL SEX IN THE LAST 6 MONTHS FROM RECALL SEX MODULE. |  |
|  | lubreak | In the last 6 months, did you ever have a condom break during anal sex? | YES 1  NO 2  DON’T KNOW 7  REFUSE TO ANSWER 8 | ‘2’,’7’ OR ‘8’ 🡪SKIP TO NEXT SECTION  SKIP TO END OF SECTION IF NO VAGINAL OR ANAL SEX IN THE LAST 6 MONTHS; |  |
|  | luUSEBrK | Did you use lubricant the time that it broke? | YES 1  NO 2  DON’T KNOW 7  REFUSE TO ANSWER 8 |  |  |
|  |  | In the last 6 months, did you ever have a condom break during vaginal sex? | YES 1  NO 2  DON’T KNOW 7  REFUSE TO ANSWER 8 |  |  |
|  |  | Did you use lubricant the time that it broke? | YES 1  NO 2  DON’T KNOW 7  REFUSE TO ANSWER 8 |  |  |
|  | LUFREE | In the last 12 months, have you been given “packets” of lubricant for free? For example, through an outreach service, drop-in center or health clinic. | YES 1  NO 2  DON’T KNOW 7  REFUSE TO ANSWER 8 |  |  |
|  | LUSOURCE | From where did you receive these packets? | SOURCE A 1  SOURCE B 2  DON’T KNOW 7  REFUSE TO ANSWER 8 |  | Fill in possible sources during pre-test |
|  |  | **SHAME, STIGMA, HARASSMENT & DISCRIMINATION** |  |  |  |
|  |  | **Please tell us how strongly you agree or disagree with each statement.** |  |  |  |
|  | STGSHM | I am ashamed that I sell/exchange sex for money, gifts, goods, or services. . | STRONGLY AGREE 1  AGREE 2  NEUTRAL 3  DISAGREE 4  STRONGLY DISAGREE 5  DON’T KNOW 9997  REFUSE TO ANSWER 9998 |  |  |
|  | STGMMEET | I am not ashamed to say I sell / exchange sex in a gathering with other people who do the same. | STRONGLY AGREE 1  AGREE 2  NEUTRAL 3  DISAGREE 4  STRONGLY DISAGREE 5  DON’T KNOW 9997  REFUSE TO ANSWER 9998 |  |  |
|  | STGHLCR | I am not ashamed to say I sell/exchange sex when I meet with a health care worker. | STRONGLY AGREE 1  AGREE 2  NEUTRAL 3  DISAGREE 4  STRONGLY DISAGREE 5  DON’T KNOW 9997  REFUSE TO ANSWER 9998 |  |  |
|  | STGTOLD | Who have you told that you sell sex or exchange sex for money?  CHECK ALL THAT APPLY. | YES NO DK NR  NO ONE 1 2 7 8  PARTNER/SPOUSE 1 2 7 8  FAMILY 1 2 7 8  FRIENDS/ACQUAINTANCES WHO ARE SEX WORKERS 1 2 7 8  FRIENDS/ACQUAINTANCES WHO ARE NOT SEX WORKERS 1 2 7 8  HEALTHCARE PROVIDERS 1 CHILDREN2 7 8  OTHER 1 2 7 8 | IF ‘NO ONE’ NO OTHER CATEGORY CAN BE CHOSEN. |  |
|  | STGFRND | In the last 12 months, which of the following people have treated you badly or excluded you **because** you sell or exchange sex? | YES NO DK NR  NO ONE 1 2 7 8  PARTNER/SPOUSE 1 2 7 8  FAMILY 1 2 7 8  FRIENDS/ACQUAINTANCES WHO ARE SEX WORKERS 1 2 7 8  FRIENDS/ACQUAINTANCES WHO ARE NOT SEX WORKERS 1 2 7 8  HEALTHCARE PROVIDERS 1 2 7 8  OTHER 1 2 7 8 |  |  |
|  | STGHEAL | Have you ever been treated unfairly or denied health care because you sell/exchange sex? | YES 1  NO 2  DON’T KNOW 7  REFUSE TO ANSWER 8 |  |  |
|  | STGHIDE | When you seek health care, do you feel the need to hide that you sell / exchange sex ? | YES 1  NO 2  DON’T KNOW 7  REFUSE TO ANSWER 8 |  |  |
|  | STGARST | Have you ever been arrested and held in a police holding cell because you sell or exchange sex? | YES 1  NO 2  DON’T KNOW 7  REFUSE TO ANSWER 8 |  |  |
|  |  | Have you ever been sent to prison because you sell or exchange sex? | YES 1  NO 2  DON’T KNOW 7  REFUSE TO ANSWER 8 |  |  |
|  | SWPOGivE | In the past 12 months, how many times have you given something (in cash or kind) to the police to avoid trouble with them?  Type ‘0’ if none. | TIMES: \|__\|__\|__\|  DON’T KNOW 997  REFUSE TO ANSWER 998 | ‘0’,’997’ OR ‘998’🡪 SKIP TO SWCARRY |  |
|  | SWPOITEM | What did you give to the police to avoid trouble with them?  CHECK ALL THAT APPLY. | Y N DK NR  MONEY 1 2 7 8  SEX 1 2 7 8  OTHER 1 2 7 8 |  |  |
|  |  | **Next we ask some questions about people with HIV.** |  |  |  |
|  | STFRTST | In your opinion, are girls and women who sell or exchange sex hesitant to test for HIV out of fear how people will react if they test positive? | YES 1  NO 2  NOT SURE 3  DON’T KNOW 7  REFUSE TO ANSWER 8 |  |  |
|  | STTLK | Do girls and women who sell or exchange sex talk badly about people living with or thought to be living with HIV? | YES 1  NO 2  IT DEPENDS 3  DON’T KNOW 7  REFUSE TO ANSWER 8 |  |  |
|  |  | **HIV KNOWLEDGE AND PERCEPTIONS** |  |  |  |
|  |  | **Thank you. Next we will ask you some questions about what you know about HIV.** |  |  |  |
|  | HKONEPAR | Can the risk of HIV transmission be reduced by having sex with only one uninfected partner who has no other partners? | YES 1  NO 2  DON’T KNOW 3  REFUSE TO ANSWER 8 |  |  |
|  | HKCONUSE | Can a person reduce the risk of getting HIV by using a condom every time they have sex? | YES 1  NO 2  DON’T KNOW 3  REFUSE TO ANSWER 8 |  |  |
|  | HKHEALTH | Can a healthy-looking person have HIV? | YES 1  NO 2  DON’T KNOW 3  REFUSE TO ANSWER 8 |  |  |
|  | HKMOSBIT | Can a person get HIV from mosquito bites? | YES 1  NO 2  DON’T KNOW 3  REFUSE TO ANSWER 8 |  |  |
|  | HKSRFOOD | Can a person get HIV by sharing food with someone who is infected? | YES 1  NO 2  DON’T KNOW 3  REFUSE TO ANSWER 8 |  |  |
|  | HKMORSK | If a condom is not used, what kind of sex puts you at the **most** risk for HIV? | ORAL SEX 1  VAGINAL SEX 2  ANAL SEX 3  MUTUAL MASTURBATION 4  ALL OF THE ABOVE EQUALLY 5  DON’T KNOW 7  REFUSE TO ANSWER 8 |  |  |
|  | HKBEHA | Do you agree with this statement: “*You are not as careful about HIV and sex now because there is better treatment for HIV?*” | AGREE 1  DON’T AGREE 2  DON’T KNOW 7  REFUSE TO ANSWER 8 |  |  |
|  |  | **REPRODUCTIVE HEALTH** |  |  |  |
|  |  | **Now some questions about family planning, pregnancies, and children. First, we would like to ask you about pregnancy.** |  |  |  |
|  | rhevrprg | Have you ever been pregnant? | YES 1  NO 2  DON’T KNOW 9997  REFUSE TO ANSWER 9998 | ‘2’,’9997’ OR ‘9998’🡪SKIP to rhtryprg |  |
|  | rhprenum | How many times have you been pregnant? | [__\|__]  DON’T KNOW 9997  REFUSE TO ANSWER 9998 | ‘0’, ‘9997’ OR ‘9998’ 🡪SKIP to RHCONUSE |  |
|  | RHPRGNOW | When were you last pregnant? | I AM PREGNANT NOW 1  WITHIN THE LAST 12 MONTHS 2  BETWEEN 12 MONTHS AND 3 YEARS AGO 3  LONGER THAN THREE YEARS AGO 4  DON’T KNOW 9997  REFUSE TO ANSWER 9998 |  |  |
|  |  | **The next few questions are about the last pregnancy that resulted in a birth. This does not include a current pregnancy***.* |  |  |  |
|  | RHANCUSE | Think about your last pregnancy that resulted in a birth. Did you go to an antenatal care (ANC) clinic? | YES 1  NO 2  DID NOT HAVE A PREGNANCY THAT RESULTED IN A BIRTH 3  DON’T KNOW 9997  REFUSE TO ANSWER 9998 | ‘2’,’3’,’9997’ OR ‘9998’🡪SKIP TO RHHIVTST |  |
|  | RHANCHIV | During that last pregnancy, were you offered an HIV test at any of your ANC visits? | YES 1  NO 2  DON’T KNOW 9997  REFUSE TO ANSWER 9998 | ‘2’,’9997’ OR ‘9998’🡪skip to rhanctp |  |
|  | rhhivtst | During that last pregnancy, how many times did you test for HIV? | NEVER 1  ONE TIME 2  TWO OR MORE TIMES 3  DON’T KNOW 9997  REFUSE TO ANSWER 9998 | ‘1’,’9997’ OR ‘9998’ 🡪SKIP to rhtptest |  |
|  | rhhivtri | During that last pregnancy, in which trimester did you last test for HIV? | FIRST TRIMESTER 1  SECOND TRIMESTER 2  THIRD TRIMESTER 3  DON’T KNOW 9997  REFUSE TO ANSWER 9998 |  |  |
|  | rhhivres | During that last pregnancy, what was the result of the last HIV test? | NEGATIVE 1  POSITIVE 2  UNCLEAR / NEITHER POSITIVE OR NEGATIVE 3  DID NOT RECEIVE RESULT 4  DON’T KNOW 9997  REFUSE TO ANSWER 9998 | IF <>’1’ 🡪SKIP TO rhtptest |  |
|  | rhhivarv | Some HIV-positive women who are pregnant take ARV medicine to reduce the risk of passing HIV on to their baby. Did you take ARVs before giving birth? | YES 1  NO 2  DON’T KNOW 9997  REFUSE TO ANSWER 9998 |  |  |
|  | RHANCTP | During the last time you were pregnant, were you offered a syphilis test? | YES 1  NO 2  DON’T KNOW 9997  REFUSE TO ANSWER 9998 | ’2’,’9997’ or ‘9998’🡪 SKip to rhtryprg |  |
|  | rhtptest | The last time you were pregnant, were you tested for syphilis? | YES 1  NO 2  DON’T KNOW 9997  REFUSE TO ANSWER 9998 | ‘2’,’9997’ or ‘9998’🡪SKIP to rhtryprg |  |
|  |  |  |  |  |  |
|  | rhtptx | Did you get treatment for your syphilis infection? | YES 1  NO 2  DON’T KNOW 9997  REFUSE TO ANSWER 9998 |  |  |
|  | RHTRYPRG | Are you currently trying to get pregnant? | YES 1  NO 2  DON’T KNOW 9997  REFUSE TO ANSWER 9998 | ‘1’,’9997’ or ‘9998’🡪SKIP to rhprdvag |  |
|  | rhcnnow | Do you use any methods to prevent yourself from getting pregnant? | YES 1  NO 2  DON’T KNOW 9997  REFUSE TO ANSWER 9998 | ‘2’,’9997’ or ‘9998 🡪SKIP to rhprdvag |  |
|  | rhtypnow | Which method do you mainly use? | PILL 1  INJECTION 2  IMPLANT / DEPO 3  CONDOMS 4  IUD 5  OTHER 9996  DON’T KNOW 9997  REFUSE TO ANSWER 9998 | IF <>‘9996’🡪SKIP TO RHPrDVAG |  |
|  | rhtypoth | You said “other”. Which of the following family planning methods do you mainly use? | I AM STERILIZED/PARTNER STERILIZED 1  PATCH 2  VAGINAL RING 3  OTHER 9996 |  |  |
|  |  | **SERVICES UPTAKE** |  |  |  |
|  |  | **Outreach** |  |  |  |
|  |  | **We will now ask you about services that peer educators or outreach workers may have given you. A peer educator is someone like you who has been trained in HIV. An outreach worker is someone employed by an organization, government or private agency, which might provide these same services to people like you.** |  |  |  |
|  | CSOREV | Has a peer educator or outreach worker ever talked to you about HIV? | YES 1  NO 2  DON’T KNOW 7  REFUSE TO ANSWER 8 | IF ‘2’,’7’ OR ‘8’🡪SKIP TO NEXT SUBSECTION |  |
|  | CSORTI | When was the last time a peer educator or outreach worker talk to you about HIV? | IN THE LAST 30 DAYS 1  IN THE LAST 3 MONTHS 2  IN THE LAST YEAR 3  MORE THAN ONE YEAR AGO 4  DON’T KNOW 7  REFUSE TO ANSWER 8 |  |  |
|  | CSORPR | What things did the outreach worker give you the last time you met one?  CHECK ALL THAT APPLY. | Y N DK NR  NOTHING 1 2 7 8  CONDOMS 1 2 7 8  LUBRICANTS 1 2 7 8  PAMPHLET OR BROCHURE 1 2 7 8  MEDICINES 1 2 7 8  OTHER 1 2 7 8 | SKIP IF PWID.  IF ‘NOTHING’ NO OTHER CATEGORY CAN BE CHOSEN. |  |
|  | CSORSV | What services did you get the last time you met a peer educator or outreach worker? CHECK ALL THAT APPLY. | Y N DK NR  HIV TESTING 1 2 7 8  TRAINING ON CONDOM USE 1 2 9997 9998TRAINING ON CONDOM USE 1 2 7 8  COUNSELING ON RISK 1 2 9997 9998COUNSELING ON RISK 1 2 7 8  REFERRAL 1 2 7 8  NOTHING 1 2 7 8  OTHER 1 2 798 | IF ‘NOTHING’ NO OTHER CATEGORY CAN BE CHOSEN |  |
|  | CSSOURCE | Which organization did that peer educator come from? | SOURCE A 1  SOURCE B 2  DON’T KNOW 7  REFUSE TO ANSWER 8 |  | Fill in possible sources during pre-test |
|  |  | **Counseling and Testing** |  |  |  |
|  |  | **We will now ask you a few questions about HIV testing.** |  |  |  |
|  | csctev | Have you ever been tested for HIV? | YES 1  NO 2  DON’T KNOW 7  REFUSE TO ANSWER 8 | ’1’🡪SKIP TO CSCTPL1 |  |
|  | csctnv | What is the main reason you have never tested for HIV? | I FEEL I AM NOT AT RISK FOR HIV 1  FEAR OF POSITIVE RESULT 2  NO MONEY TO GET TESTED 3  NO TIME TO GET TESTED 4  STIGMA BY HEALTH CARE WORKERS 5  OTHER 6  REFUSE TO ANSWER 8 | SKIP TO END OF SECTION |  |
|  | csctmm2 | If you have a main sex partner, have you ever tested for HIV together with him? | I DON’T HAVE A MAIN SEX PARTNER 1  YES – WE TESTED TOGETHER 2  NO – WE NEVER TESTED TOGETHER 3  NO—PARTNER WAS TESTED BUT WITHOUT ME 4  DON’T KNOW 7  REFUSE TO ANSWER 8 |  |  |
|  | CSCTTI | When did you last test for HIV? | IN THE LAST 6 MONTHS 1 BETWEEN 6-12 MONTHS AGO 2 MORE THAN 12 MONTHS AGO 3 DON’T KNOW 7 REFUSE TO ANSWER 8 | If CSCTTI^=3 skip to CSCTPAR |  |
|  | CSCNOT | Why have you not tested in the last 12 months? | I FEEL I AM NOT AT RISK FOR HIV 1  FEAR OF POSITIVE RESULT 2  NO MONEY TO GET TESTED 3  NO TIME TO GET TESTED 4  STIGMA BY HEALTH CARE WORKERS 5  OTHER 6  REFUSE TO ANSWER 8 |  |  |
|  | csctpl2 | Where did you have your last test? | TESTING AND COUNSELING CENTER 1  HEALTH CLINIC, HOSPITAL, OR SIMILAR 2  OUTREACH/MOBILE TESTING 3  OTHER 6  DON’T KNOW 7  REFUSE TO ANSWER 8 | go to csctwho | Complete possible locations during pre-test |
|  | csctpl3 | Where did you get tested? | IN MY HOME 1  AT MY WORK 2  WHERE I HANG OUT 3  OUTREACH/MOBILE TESTING 4  OTHER 6  DON’T KNOW 7  REFUSE TO ANSWER 8 | if <>’6’ skip csctpl4 |  |
|  | csctpl4 | You said “Other”. Where was that? | SCHOOL 1  PRISON 2  CHURCH 3  OTHER 6  DON’T KNOW 7  REFUSE TO ANSWER 8 |  |  |
|  | csctwho | Who tested you? Choose the best answer. | GOVERNMENT WORKER 1  *NGO/CBO NAME A* 2  *NGO/CBO NAME B* 3  *NGO/CBO NAME C* 4  OTHER PRIVATE OR COMMERCIAL PLACE 5  OTHER 6  DON’T KNOW 7  REFUSE TO ANSWER 8 |  |  |
|  | CSCTRS1 | Why did you test for HIV? | HEALTH CARE WORKER OR OUTREACH WORKER OFFERED TEST 1  I JUST WANTED TO KNOW 2  FELT AT RISK OR SICK 3  GOT A NEW PARTNER 4  OTHER 6  DON’T KNOW 7  REFUSE TO ANSWER 8 | if <> ’6’ skip TO csctT1 |  |
|  | CSCTRS2 | You said “Other”. Which of the following best fit your reason to test? | EMPLOYER ASKED ME TO TEST 1  PRE-MARITAL TESTING 2  PARTNER ASKED ME TO TEST 3  I WAS TESTED WITHOUT MY CONSENT  I WAS SICK WITH TB  I WAS PREGNANT  I TEST REGULARLY FOR MY OWN HEALTH  OTHER 6  DON’T KNOW 7  REFUSE TO ANSWER 8 |  |  |
|  | csctti | When did you last test for HIV? | IN THE LAST ONE MONTH?  IN THE LAST 6 MONTHS 1  BETWEEN 6-12 MONTHS AGO 2  MORE THAN 12 MONTHS AGO 3  DON’T KNOW 7  REFUSE TO ANSWER 8 |  |  |
|  | csctrs | What was the result of your last test? | POSITIVE 1  NEGATIVE 2 UNCLEAR / NEITHER POSITIVE OR NEGATIVE 3  DID NOT RECEIVE RESULT 4  DON’T KNOW 7  REFUSE TO ANSWER 8 | IF NEGATIVE GO TO 164  IF POSITIVE SKIP… |  |
|  | CSCTsw | When you last tested for HIV, did you tell the counselor/health care worker that you sell / exchange sex for money? | YES 1  NO 2  DON’T KNOW 9997  REFUSE TO ANSWER 9998 |  |  |
|  | CSCTqu1 | Which of the following best describes the pre and post test counseling you received? | RESPECTFUL, CARING, UNDERSTANDING 1  DISRESPECTFUL, UNCARING, STIGMATIZING, UNCOMFORTABLE 2  NEITHER RESPECTFUL NOR DISRESPECTFUL 3  DON’T KNOW 7  REFUSE TO ANSWER 8 |  |  |
|  |  | **Disclosure** |  |  |  |
|  | csctTL | Of the following people, who have you told that you are HIV positive?  CHECK ALL THAT APPLY. | Y N DK NR  NO ONE 1 2 7 8  SPOUSE 1 2 7 8  CLIENT 1 2 7 8  DOCTOR 1 2 7 8  FRIEND – FELLOW SEX WORKER 1 2 7 8  FRIEND – NOT INVOLVED IN SEX WORK 1 2 7 8  PARENTS / AUNTS /UNCLES  SIBLINGS/COUNSINS  CHILD/CHILDREN  1 2 7 8  PASTOR/PRIEST 1 2 7 8  OTHER 1 2 7 8  2 7 8 |  |  |
|  | cscFP | Was the last test your first positive test? | YES 1  NO – I TESTED POSITIVE BEFORE ALREADY 2  DON’T KNOW 7  REFUSE TO ANSWER 8 | ‘1’🡪SKIP TO CSCFPY  ’7’ OR ‘8’🡪SKIP TO next subsection |  |
|  | cscFPy | What year did you first test positive? | \|__ \|__ \|__ \|__\|  MIN: 1900  MAX: SYSTEMYEAR  DON’T KNOW 7  REFUSE TO ANSWER 8 |  |  |
|  | cscFPm | What month did you first test positive? | \|__ \|__\|  RANGE: 1-12  IF CSCTYY=SYSTEMYEAR, THEN MAXIMUM=SYSTEMMONTH  DON’T KNOW 7  REFUSE TO ANSWER 8 |  |  |
|  |  | **Care and treatment** |  |  |  |
|  |  | **Thank you. You told us you tested positive for HIV. Now we will ask you about your participation in a support group.** |  |  |  |
|  | csPSev | Have you ever attended a peer support group for HIV-positive people? | YES 1  NO 2  DON’T KNOW 7  REFUSE TO ANSWER 8 |  |  |
|  | csPSFQ | How frequently do you attend a peer support group? | DO NOT ATTEND ANYMORE 1  LESS THAN ONCE A MONTH 222  AT LEAST ONCE A MONTH 333  WEEKLY OR MORE 4  DON’T KNOW 7  REFUSE TO ANSWER 8 |  |  |
|  | CSORG | Which organization organizes that support group? | SOURCE A 1  SOURCE B 2  DON’T KNOW 7  REFUSE TO ANSWER 8 |  | Fill in possible sources during pre-test |
|  |  | **Linkage to care** |  |  |  |
|  |  | **You told us you tested positive for HIV. Many people with HIV register with a health care provider to get care. By “care” we mean someone goes for check-ups to a health care provider or gets ARVs for their HIV infection. The next few questions are about the first time you saw a provider for your HIV.** |  |  |  |
|  | CSLKEV | Have you ever attended any clinic or health care facility for your HIV care? | YES 1  NO 2  DON’T KNOW 7  REFUSE TO ANSWER 8 | ‘1’🡪SKIP TO CSLKTY1 |  |
|  | CSlknv | What is the main reason you have never visited a health care provider for HIV care? | FEEL HEALTHY 1  STIGMA, DON’T WANT OTHERS TO KNOW 2  COST/DISTANCE TO CLINIC 3  POOR ATTITUDE OF HEALTH CARE WORKERS 4  WAITING TIME OR CLINIC HOURS NOT GOOD 5  OTHER 6  DON’T KNOW 7  REFUSE TO ANSWER 8 |  |  |
|  | CSLKTI | After you first tested positive, how long did it take you to go back to the health care provider for ongoing care? | LESS THAN ONE MONTH 1  BETWEEN 1 AND 3 MONTHS 2  BETWEEN 4 AND 12 MONTHS 3  LONGER THAN ONE YEAR 4  DON’T KNOW 7  REFUSE TO ANSWER 8 |  |  |
|  |  | **Care** |  |  |  |
|  |  | **Thank you. The next few questions are about the care you may receive now.** |  |  |  |
|  | CSCANW | After learning of your HIV diagnosis, have you ever received HIV medical care from a health care provider? | yes 1  no 2  REFUSE TO ANSWER 8 | ‘1’🡪SKIP TO CSCALST |  |
|  | CSCARS | What is the main reason that you have never seen a health care provider for HIV medical care? | THE facility is too far AWAY 1  i don’t know where TO GET HIV MEDICAL CARE 2  COST OF CARE 3  COST OF TRANSPORT 4  I feel healthy/not sick 5  i fear people will know that i have HIV if I go to a clinic 6  I’M TAKING TRADITIONAL MEDICINE 7  RELIGIOUS REASONS 8  other 96  don’t know 98  reFUSED 99 |  |  |
|  | CSCALST | After learning your HIV diagnosis, what month and year did you first see a health care provider for HIV medical care? | MONTH ___ ___  YEAR ___ ___ ___ ____  DON’T KNOW month 98  REFUSED MONTH 99  don’t KNOW year 9998  REFUSED YEAR 9999 |  |  |
|  | CSLAST | What month and year did you last see a health care provider for HIV medical care? | MONTH ___ ___  YEAR ___ ___ ___ ____  DON’T KNOW MONTH 98  REFUSED MONTH 99  DON’T KNOW YEAR 9998  REFUSED 9999 |  |  |
|  | CSNONOW | What is the main reason for not seeing a health care provider for HIV medical care in the past 6 months? | THE facility is too far AWAY 1  i don’t know where TO GET HIV MEDICAL CARE 2  COST OF CARE 3  COST OF TRANSPORT 4  I feel healthy/not sick 5  i fear people will know that i have HIV if I go to a clinic 6  I’M TAKING TRADITIONAL MEDICINE 7  RELIGIOUS REASONS 8  other 96  don’t know 98  reFUSED =99 |  |  |
|  |  | **Cotrimoxazole** |  |  |  |
|  | CSCXIN | **Now some questions about Cotrimoxazole or Cotrim. Cotrim is a medicine recommended for people with HIV, even if they have not started treatment. It helps prevent certain infections but it does not treat HIV.** |  |  |  |
|  | CSCXnw | Are you currently taking cotrimoxazole? | YES 1  NO 2  DON’T KNOW 7  REFUSE TO ANSWER 8 |  |  |
|  | CXNOT | Why are you not taking cotrimoxazole? | FEEL HEALTHY 1  FEAR OF STIGMA 2  NOT OFFERED BY CLINIC 3  NO TIME/CLINIC TOO FAR 4  COST 5  OTHER 6  DON’T KNOW 7  REFUSE TO ANSWER 8 |  |  |
|  |  | **CD4+ T cell count** |  |  |  |
|  |  | **We will now ask you a few questions about your “CD4” or “T-cell” count. The CD4 count tells how sick you are with HIV or how weak your immune system is and if you need to take ARVs.** |  |  |  |
|  | CScdEV | Have you ever had your CD4 tested? | YES 1  NO 2  DON’T KNOW 7  REFUSE TO ANSWER 8 | ‘2’,’7’,’8’ 🡪 SKIP TO NEXT SUBSECTION |  |
|  | CScdEV | When did your health care provider last test your CD4 count? | IN THE LAST 6 MONTHS 1  BETWEEN 7 AND 12 MONTHS AGO 2  MORE THAN 12 MONTHS AGO 3  DON’T KNOW 7  REFUSE TO ANSWER 8 |  |  |
|  |  | **ART** |  |  |  |
|  |  | **Thank you. Now I will ask you some questions on anti-retroviral treatment, also called ARVs, to treat HIV.** |  |  |  |
|  | CSTRev | Have you ever taken ARVs, that is, antiretroviral medication, to treat your HIV infection? | YES 1  NO 2  DON’T KNOW 7  REFUSE TO ANSWER 8 | ‘1’🡪 SKIP TO csTRYY |  |
|  | CSTRNV1 | What is the main reason you never started taking ARVs? | MY CD4 COUNT IS STILL HIGH 1  MY CD4 COUNT IS UNKNOWN 2  I AM ON A WAITING LIST TO START 3  HEALTH CARE PROVIDER TOLD ME ITS TOO EARLY TO START 4  I HAVE TB  I DON’T WANT TO TAKE THEM 5  OTHER REASON 6  DON’T KNOW 7  REFUSE TO ANSWER 8 | IF <>’6’ 🡪 SKIP TO CSVLEV |  |
|  | CSTRNV2 | You said other. Which of the following is the main reason you never started taking ARVs? | NO CLINIC NEAR ME OFFERS IT 1  HEALTH CARE PROVIDER REFUSED TO GIVE ME ARVs 2  FEAR OF STIGMA OR THAT OTHERS FIND OUT 3  COSTS TOO MUCH 4  I AM NOT FROM PNG 5  OTHER 6  DON’T KNOW 7  REFUSE TO ANSWER 8 |  |  |
|  | CS7MSG | Do you currently taking ARVs? | YES 1  NO 2  DON’T KNOW 7  REFUSE TO ANSWER 8 | ‘1’🡪CSTRSV  IF |  |
|  | CSTRSP1 | Some people decide they no longer want to take ARVs. Others want to take it but have trouble getting it. Which best describes the reason you stopped taking ARVs? | I DIDN’T WANT TO TAKE ARVs ANYMORE 1  IT WAS DIFFICULT FOR ME TO GET ARVs 2  BOTH 3  OTHER 6  DON’T KNOW 7  REFUSE TO ANSWER 8 | IF<>’1’🡪CSTRSP3 |  |
|  | CSTRSP2 | Which answer best fits why you found it difficult to regularly get ARVs? | CLINIC TOO FAR, TRANSPORT COSTS TOO HIGH 1  COSTS AT CLINIC TOO HIGH 2  POOR SERVICE AT CLINIC 3  CLINIC RAN OUT OF ARVs 4  OTHER 6  REFUSE TO ANSWER 8 | SKIP TO END OF SUBSECTION |  |
|  | CSTRSP3 | Which answer best fits why you stopped taking ARVs? | FEEL HEALTHY 1  TOO MANY SIDE EFFECTS 2  GOD HAS HEALED ME 3  OTHER 6  REFUSE TO ANSWER 8 |  |  |
|  |  | **TUBERCULOSIS** |  |  |  |
|  |  | **The next few questions are about tuberculosis or TB. All people with HIV should be “screened” for TB. With “screening”, we mean health care staff ask you about if you have a cough, fever, or sudden weight loss.** |  |  |  |
|  | csTBsc1 | During the last 12 months, during any of your visits to the HIV clinic, were you asked if you had night sweats, chronic cough, fever, and unexplained weight loss? | YES 1  NO, I WAS NOT ASKED 2  NO, I AM CURRENTLY ON TB TREATMENT 3  DON’T KNOW 7  REFUSE TO ANSWER 8 | , ‘2’,’3’,’7’,’8’ 🡪skip to end of SUBsection |  |
|  | csTBsc1 | In the last 12 months, how often were you screened with these questions? | AT EVERY VISIT 1  ON SOME VISITS 2  ON ONE VISIT 3  DON’T KNOW 7  REFUSE TO ANSWER 8 |  |  |
|  | csTBTT | In the last 12 months, were any of these tests done to look for TB? This could include a chest x-ray or sputum test.  CHECK ALL THAT APPLY. | Y N DK NR  NONE OF THESE 1 2 7  CHEST X-RAY 1 2 7 8  SPUTUM TEST 1 2 7 8 | IF ‘NONE OF THESE’🡪SKIP TO CSTREV |  |
|  | csTBrs | What was the result of your last TB test? | TB-NEGATIVE 1  TB-POSITIVE 2DON’T KNOW 7  REFUSE TO ANSWER 8 |  |  |
|  | csTBTR1 | Did you ever get treatment for TB? | YES 1  NO 2  DON’T KNOW 7  REFUSE TO ANSWER 8 |  |  |
|  | CSTBTR2 | When did you take treatment for TB? | BEFORE I TESTED HIV-POSITIVE 1  AFTER I TESTED HIV-POSiTIVE 2  BOTH BEFORE AND AFTER I TESTED HIV-POSITIVE 3  DON’T KNOW 7  REFUSE TO ANSWER 8 |  |  |
|  | CSTBIPT | Are you taking medicine to prevent you from getting TB? | YES 1  NO 2  DON’T KNOW 9997  REFUSE TO ANSWER 9998 |  |  |
|  |  | **SEXUALLY TRANSMITTED INFECTIONS** |  |  |  |
|  | STABNF | During the last 12 months, have had an abnormal discharge from your vagina? This may include an unusual smell, color, or texture. | YES 1  NO 2  DON’T KNOW 9997  REFUSE TO ANSWER 9998 |  |  |
|  | STULCF | In the last 12 months, have you had an ulcer or sore on or near your vagina? | YES 1  NO 2  DON’T KNOW 7  REFUSE TO ANSWER 8 |  |  |
|  | STULCM | In the last 12 months, have you found an ulcer or sore on or near a sex partner’s penis? | YES 1  NO 2  DON’T KNOW 7  REFUSE TO ANSWER 8 |  |  |
|  | STSTdHCW | Did you see a healthcare provider because of these symptoms? | YES 1  NO 2  DON’T KNOW 7  REFUSE TO ANSWER 8 | ‘1’🡪SKIP TO STSTDX |  |
|  | STSTdxPH | Did you take treatment? | YES 1  NO 2  DON’T KNOW 7  REFUSE TO ANSWER 8 | SKIP TO STSTHPV |  |
|  | STSTime | How long did it take you to go see the healthcare provider since the appearance of these symptoms? | LESS THAN ONE WEEK 1  MORE THAN ONE WEEK LESS THAN ONE MONTH 2  MORE THAN ONE MONTH 3  DON’T KNOW 7  REFUSE TO ANSWER 8 |  |  |
|  | STTELL | Did the healthcare provider tell you that you had a sexually transmitted infection? | YES 1  NO 2  DON’T KNOW 7  REFUSE TO ANSWER 8 | ‘2’,’9997’ OR ‘9998’🡪SKIP TO STSTHPV |  |
|  | STTRT | Did you get treatment for this sexually transmitted infection? | YES 1  NO 2  DON’T KNOW 7  REFUSE TO ANSWER 8 | ‘2’,’9997’ OR ‘9998’🡪SKIP TO STSTHPV |  |
| **PHYSICAL VIOLENCE** |  | **Physical Violence** |  |  |  |
| NA | PVMSG1 | We would now like you to answer some questions about whether or not certain things have happened to you. Please remember that your answers are confidential. If you would like to speak to a health care worker or social worker or anyone else about your experiences, please let the survey know. |  |  |  |
| 1. 190 |  | Has anyone ever done any of these things to you:   - Punched, kicked, whipped, or beat you with an object - Slapped you, threw something at you that could hurt you, pushed you or shoved you - Choked smothered, tried to drown you, or burned you intentionally - Used or or threatened you with a knife, gun or other weapon? | YES 1  NO 2  DON’T KNOW 7  REFUSE TO ANSWER 8 |  |  |
| 1. 191 |  | Thinking about all these experiences that we just discussed, whether someone has done the following:   - Punched, kicked whipped or beat you with an object - Slapped you, threw something at you that could hurt you, pushed you or shoved you - Choked, smothered, tried to drown you or burned you intentionally - Used or threatened you with a knife, gun or other weapon   Did you try to seek professional help or services for any of these incidents from any of the following?  SELECT ALL THAT APPLY. | I DID NOT TRY TO SEEK HELP = A  HEALTHCARE PROFESSIONAL = B  POLICE OR OTHER SECURITY PERSONNEL = C  SOCIAL WORKER, COUNSELOR OR NON-GOVERNMENTAL ORGANIZATION = D  RELIGIOUS LEADER = E  OTHER = X  DON’T KNOW = Y  REFUSED = Z |  |  |
| 1. 192 |  | What was the main reason that you did not try to seek professional help or services? | DID NOT KNOW SERVICES WERE AVAILABLE = 1  SERVICES NOT AVAILABLE = 2  AFRAID OF GETTING IN TROUBLE = 3  ASHAMED FOR SELF/FAMILY = 4  COULD NOT AFFORD SERVICES = 5  DID NOT THINK IT WAS A PROBLEM = 6  FELT IT WAS MY FAULT = 7  AFRAID OF BEING ABANDONED = 8  DID NOT NEED/WANT SERVICES = 9  AFRAID OF MAKING SITUATION WORSE = 10  OTHER = 96  DON’T KNOW = 97  REFUSED = 98 |  |  |
| 1. **SEXUAL VIOLENCE** | PVYRBEH | In any of the times in the last 12 months when these happened, do you think it was because you sell or exchange sex? | YES 1  no 2  don’t know 7  refuse to answer 8 |  |  |
| NA | SVMSG1 | We would like to ask you some questions about sex that may have happened to you. When we say ‘sex’ here we are only talking about vaginal sex and anal sex. With vaginal sex we mean a penis enters a vagina. With anal sex we mean a penis enters a person’s anus (butt).   Sometimes people use lies, tricks, threats or physical force to make someone else have sex with them when they do not want to. For the next few questions, we will ask you about when someone tricked you, lied to you or threatened you to make you have sex with them, and NOT about when someone may have physically forced you to have sex.   Please remember that your answers are confidential. If you would like to speak to a health care worker or social worker or anyone else about your experiences, please let the Interview Facilitator know. |  |  |  |
| 1. 193 | SVCEVFR | In your lifetime, how many times has anyone ever physically forced you to have sex?  CODE ‘00” IF NONE | NUMBER OF TIMES ____  DON’T KNOW 7  REFUSE TO ANSWER 8 | ‘1’,’7’,’8’→ SKIP TO SVRFBEH |  |
| 1. 194 |  | How old were you the first time someone physically forced you to have sex? | AGE IN YEARS ____  DON’T KNOW 7  REFUSE TO ANSWER 8 |  |  |
| 1. 195 |  | The first time someone physically forced you to have sex, was the person male or female? If it was more than one person, what was the sex of the person you knew the best? | MALE 1  FEMALE 2  DON’T KNOW 7  REFUSE TO ANSWER 8 |  |  |
| 1. 196 |  | What was this person’s relationship to you? If it was more than one person, what was the relationship with the person you knew the best? | boyfriend/GIRLFRIEND/LIVE-IN PARTNER/SPOUSE = 1  EX-BOYFRIEND/GIRLFRIEND/PARTNER/SPOUSE = 2  RELATIVE/FAMILY MEMBER = 3  classmate/schoolmate = 4  teacher = 5  police/security officer/military= 6  employer = 7  neighbor = 8  community/  religious leader = 9  friend = 10  stranger = 11  other = 96  DOn’t know = 97  refused = 98 |  |  |
| 1. 197 |  | In the last 12 months, did someone physically force you to have sex? | YES 1 NO 2 DON’T KNOW 7 REFUSE TO ANSWER 8 |  |  |
| 1. 198 |  | In the last 12 months, did a partner physically force you to have sex?  By partner, I mean a live-in partner whether or not you were married at the time. | YES 1  NO, DID NOT FORCE 2  NO, DID NOT HAVE A LIVE-IN PARTNER IN THE LAST 12 MONTHS 3  DON’T KNOW 7  REFUSE TO ANSWER 8 |  |  |
| 1. 199 |  | The last time someone tried to make you have sex against your will but did not succeed, was the person male or female? If it was more than one person, what was the sex of the person you knew the best? | MALE 1  FEMALE 2  DON’T KNOW 7  REFUSE TO ANSWER 8 |  |  |
| 1. 200 |  | The last time this happened, what was your relationship to the person who did this? If it was more than one person, what was your relationship with the person you knew the best? | boyfriend/GIRLFRIEND/LIVE-IN PARTNER/SPOUSE = 1  EX-BOYFRIEND/GIRLFRIEND/PARTNER/SPOUSE = 2  RELATIVE/FAMILY MEMBER = 3  classmate/schoolmate = 4  teacher = 5  police/security officer/military= 6  employer = 7  neighbor = 8  community/  religious leader = 9  friend = 10  stranger = 11  other = 96  DOn’t know = 97  refused = 98 |  |  |
| 1. 201 |  | After any of these unwanted sexual experiences, did you try to seek professional help or services from any of the following?  SELECT ALL THAT APPLY. | I DID NOT TRY TO SEEK HELP = A  HEALTHCARE PROFESSIONAL = B  POLICE OR OTHER SECURITY PERSONNEL = C  SOCIAL WORKER, COUNSELOR OR NON-GOVERNMENTAL ORGANIZATION = D  RELIGIOUS LEADER = E  OTHER = X  DON’T KNOW = Y  REFUSED = Z |  |  |
| 1. 202 |  | What was the main reason that you did not try to seek professional help or services? | DID NOT KNOW SERVICES WERE AVAILABLE = 1  SERVICES NOT AVAILABLE = 2  AFRAID OF GETTING IN TROUBLE = 3  ASHAMED FOR SELF/FAMILY = 4  COULD NOT AFFORD SERVICES = 5  DID NOT THINK IT WAS A PROBLEM = 6  FELT IT WAS MY FAULT = 7  AFRAID OF BEING ABANDONED = 8  DID NOT NEED/WANT SERVICES = 9  AFRAID OF MAKING SITUATION WORSE = 10  OTHER = 96  DON’T KNOW = -8  REFUSED = -9 |  |  |
|  | SVCYRFRQ | In any of the times in the last 12 months when these happened, do you think it was because you sell/exchange sex? | YES 1  no 2  don’t know 7  refuse to answer 8 |  |  |
|  |  | The last set of questions are about where you accessed services in 2014 and any NGO or community organization you might have been a member of. | | | |
|  | MQTEST | In 2014, at which of these health facilities did you test for HIV? | Y N DK NR  Poro Sapot Clinic 1 2 7 8  Anglicare Stop AIDS 1 2 7 8 Koki Clinic 1 2 7 8 Hederu Clinic, POM General Hospital 1 2 7 8  Laws Road 1 2 7 8  9 Mile Clinic 1 2 7 8  Kaugere Clinic 1 2 7 8  St Joseph’s Medical Centre 1 2 7 8  St Ferdanaz  1 2 7 8  St John’s, Gerehu 1 2 7 8  6 Mile Clinic 1 2 7 8 |  |  |
|  | MQSTD | In 2014, at which of these health facilities did you get treated for an STD? | Y N DK NR  Poro Sapot Clinic 1 2 7 8  Anglicare Stop AIDS 1 2 7 8 Koki Clinic 1 2 7 8 Hederu Clinic, POM General Hospital 1 2 7 8  Laws Road 1 2 7 8  9 Mile Clinic 1 2 7 8  Kaugere Clinic 1 2 7 8  St Joseph’s Medical Centre 1 2 7 8  St Ferdanaz  1 2 7 8  St John’s, Gerehu 1 2 7 8  6 Mile Clinic 1 2 7 8 |  |  |
|  | MQMEM | In 2014, did you belong to any of these NGOs or community organizations? | Y N DK NR  Kapul Champions 1 2 7 8  Friends Frangipani 1 2 7 8  Igat Hope 1 2 7 8 |  |  |
|  | MQACT | In 2014, did you participate in an activity of any of these NGOs or community organizations? | Y N DK NR  Tingim Laip 1 2 7 8  Poro Sapot 1 2 7 8  Kapul Champions 1 2 7 8  Friends Frangipani 1 2 7 8  Igat Hope 1 2 7 8  Salvation Army 1 2 7 8  Hope Worldwide 1 2 7 8  Four Square / Living light 1 2 7 8  Women in Hope 1 2 7 8  Anglicare Stop AIDS 1 2 7 8  PNG DLA (Development Law Association) 1 2 7 8  Friends Foundation 1 2 7 8 |  |  |
