# Supplementary material for: One size does not fit all: HIV prevalence and correlates of risk for men who have sex with men, transgender women in multiple cities in Papua New Guinea
Source: BMC Public Health. 2019 May 22;19:623. doi: 10.1186/s12889-019-6942-7 (PMC6532262; doi:10.1186/s12889-019-6942-7)
Supplement: Supplementary file 2 — MSM TGW Questionnaire, MSM/TGW questionnaire, Quantitative interview questionnaire used with MSM/TGW participants. (DOCX 112 kb) [file 12889_2019_6942_MOESM2_ESM.docx]

**MSM/TGW QUESTIONNAIRE**

READ TO THE PARTICIPANT:

Thank you for participating in the survey. Please remember that your answers to this survey are completely confidential, your name will not be attached to any of your answers, and no one other than me will know your answers. Your answers will be coded so we will not know who they belong to. So please answer as honestly as possible.

I would like to begin the interview by asking some questions about you.

| **NO** | **VARIABLE ID** | **QUESTION** | **RESPONSE OPTIONS** | **SKIPS** | **COMMENTS** |  |  |  |  |  |  |  |  |  |
| --- | --- | --- | --- | --- | --- | --- | --- | --- | --- | --- | --- | --- | --- | --- |
| **BACKGROUND** | | | | | |  |  |  |  |  |  |  |  |  |
| 1 | DESEXBTH | What was your sex at birth? | MALE 1 FEMALE 2 OTHER 3 DON’T KNOW 7 REFUSE TO ANSWER 8 |  |  |  |  |  |  |  |  |  |  |  |
| 2 | DESEXNOW | Do you consider yourself as man, woman, or transgender or other? | MALE 1 FEMALE 2 TRANSGENDER 3 OTHER 4  DON’T KNOW 7 REFUSE TO ANSWER 8 |  |  |  |  |  |  |  |  |  |  |  |
| **3** | DEAGENUM | How old were you at your last birthday? | AGE IN COMPLETED YEARS: [__\|__] MIN: 1 MAX: CURRENT YEAR-DEAGEY  DON’T KNOW 97  REFUSE TO ANSWER 98 |  |  |  |  |  |  |  |  |  |  |  |
| 4 | DEREADWR | Can you read and write? | CANNOT READ AND WRITE 1  CAN READ ONLY 2  CAN READ AND WRITE 3  DON’T KNOW 7  REFUSE TO ANSWER 8 |  |  |  |  |  |  |  |  |  |  |  |
| 5 | DEEDEVER | Have you ever attended school? | YES 1 NO 2 DON’T KNOW 7  REFUSE TO ANSWER 8 | ‘2’,’7’ OR ‘8’→ SKIP TO DEMARSTA |  |  |  |  |  |  |  |  |  |  |
| 6 | DEEDHIGH | What is the highest level/grade/year of school you completed: | PRIMARY 1 SECONDARY 2 HIGHER 3 DON’T KNOW 7  REFUSE TO ANSWER 8 |  | Use responses based on local context. OPTION A |  |  |  |  |  |  |  |  |  |
| 7 | DEMARSTA | What is your current marital status? | SINGLE, NEVER MARRIED 1 MARRIED ONE WOMAN 2  MARRIED TO MORE THAN ONE WOMAN (polygamist) 3  MARRIED TO A MAN 4 SEPARATED/DIVORCED 5 WIDOWED 6 DON’T KNOW 7 |  |  |  |  |  |  |  |  |  |  |  |
| 8 | DELIVESX | Are you currently living with a sexual partner? | YES 1 NO 2 DON’T KNOW 7 REFUSE TO ANSWER 8 |  |  |  |  |  |  |  |  |  |  |  |
| 9 | DEREG | Do you sleep in the same place most nights?? | YES 1  NO 2  DON’T KNOW 7  REFUSE TO ANSWER 8 |  |  |  |  |  |  |  |  |  |  |  |
| 10 | DECURLIV | In which neighborhood do you currently live? | Boroko 1  Gerehu 2  Hanubada 3  Gordons 4  Waigani 5  Murray Barracks 6  Morata 7  Garden Hill 8  Taurama 9  Erima 10  Koki 11  Downtown Moresby 12  Pari/ Kaugere / Kilakila 13  Porebada 14  Bomana 15  Konedobu 16  Pagha Hills 17  6 Mile 18  7 Mile 19  8 Mile 20  9 Mile 21  Along the Hiritana Higway 22  Along the Magi Highway 23  Other 24  (Specify)  DON’T KNOW 97  REFUSE TO ANSWER 98 |  |  |  |  |  |  |  |  |  |  |  |
| 11 | DERESIDE | How long have you been visiting or lived here in Port Moresby/Lae/Hagen?  ENTER ‘00’ IF LESS THAN 1 YEAR. | # OF YEARS: [__\|__]  MIN: 0  MAX: 96  DON’T KNOW 97  REFUSE TO ANSWER 98 |  |  |  |  |  |  |  |  |  |  |  |
| 12 | DEAWAYT | In the last 6 months, how many times have you been away from home for one or more nights? By “home”, we mean the place you usually live. By “away from home”, we mean spending one or more nights away from the place you usually live. This could be even in the same province.  ENTER ‘000’ IF NONE. | # TIMES [__\|__\|__]  MIN: 000  MAX: 180  DON’T KNOW 997  REFUSE TO ANSWER 998 | ‘0’ OR ‘997’ OR ‘998’🡪 SKIP TO DEEMPSRC |  |  |  |  |  |  |  |  |  |  |
| 13 | DEAWAYM | In the last 6 months, have you been away from home for more than one month at a time? | YES 1  NO 2  DON’T KNOW 7  REFUSE TO ANSWER 8 |  |  |  |  |  |  |  |  |  |  |  |
| 14 | DEEMPSRC | What is your main source of income? | Private Company 1  Public Servant in Government 2  No income 3  Police 4  Security 5  Street sales 6  NGO 7  Sex work 8  OTHER 96  DON’T KNOW 97  REFUSE TO ANSWER 98 |  |  |  |  |  |  |  |  |  |  |  |
| 15 | DEINCOME | How much do you earn in a normal month? | AMOUNT_______  DON’T KNOW 9997  REFUSE TO ANSWER 9998 |  |  |  |  |  |  |  |  |  |  |  |
| 16 | DEchiliv | How many children do you have currently living with you that you are responsible for (including those you may have adopted or care for)?  ENTER ‘0’ IF NONE. | NUMBER [__\|__]  MIN: 0  MAX: 96  DON’T KNOW 97  REFUSE TO ANSWER 98 |  |  |  |  |  |  |  |  |  |  |  |
| 17 | DEETHNIC | To which [ethnic group/race/tribe] do you belong? | ETHNICITY/RACE A 1  ETHNICITY/RACE B 2  ETHNICITY/RACE C 3  MIXED ETHNICITY/RACE 4  OTHER 6  DON’T KNOW 7  REFUSES TO ANSWER 8 |  |  |  |  |  |  |  |  |  |  |  |
| 18 | DERELIG | What religion are you? | CATHOLIC 1  ANGLICAN 2  UNITED CHURCH 3  SEVENTH DAY ADVENTIST 4  REVIVAL CHURCH PNG 5  FOUR SQUARE CHURCH 6  ASSEMBLY OF GOD 7  SALVATION ARMY8  LUTHERAN 9  BAHAI 10  MUSLIM 11  OTHER 66  DON’T KNOW 97  REFUSES TO ANSWER 98 |  |  |  |  |  |  |  |  |  |  |  |
| 19 | DERGO | How many times did you go to church or place of worship in the last month? | VALUE [ __I__I__]  DON’T KNOW 9997  REFUSE TO ANSWER 9998 |  |  |  |  |  |  |  |  |  |  |  |
|  |  | *Next we will ask you some questions on depression. Over the last 2 weeks, how often have you been bothered by any of the following problems:* | | | |  |  |  |  |  |  |  |  |  |
| 20 | dpintrst | Little interest or pleasure in doing things | NOT AT ALL 0  SEVERAL DAYS 1  MORE THAN HALF THE DAYS 2  NEARLY EVERY DAY 3  REFUSED 8 |  |  |  |  |  |  |  |  |  |  |  |
| 21 | dpdown | Feeling down, depressed, or hopeless | NOT AT ALL 0  SEVERAL DAYS 1  MORE THAN HALF THE DAYS 2  NEARLY EVERY DAY 3  REFUSED 8 |  |  |  |  |  |  |  |  |  |  |  |
| **GENDER IDENTITY AND SEXUAL ORIENTATION** | | | | | |  |  |  |  |  |  |  |  |  |
| NA | SO1MSG | The next few questions are about your gender identity and sexual orientation. All of your answers are confidential. By homosexual/gay/lesbian we mean that you identify as someone who is sexually attracted to someone of the same sex. By bisexual we mean someone who identifies as being attracted to both men and women. By heterosexual we mean that you identify as someone who is attracted to the opposite sex. | | | |  |  |  |  |  |  |  |  |  |
| 22 | SOMIDENT | Which would you describe yourself as (read responses): | GAY  HOMOSEXUAL  /MSM / MEN OF DIVERSE SEXUALITIES (MDS) 1 BISEXUAL 2 STRAIGHT  HETEROSEXUAL 3 OTHER 6 DON’T KNOW 7 REFUSED TO ANSWER 8 |  |  |  |  |  |  |  |  |  |  |  |
| 23 | SOATTRA | Who are you most sexually attracted to? | ONLY OR MOSTLY MALE 1 EQUALLY MALE AND FEMALE 2 ONLY OR MOSTLY FEMALE 3  TG 4 NONE 5 DON’T KNOW 7 REFUSE TO ANSWER 8 |  |  |  |  |  |  |  |  |  |  |  |
| 24 | SOMTEL | Other than your male/TG sex partners, whom have you told that you have sex with men/TG?      CHECK ALL THAT APPLY | Y N DK NR  NO ONE 1 2 7 8 FAMILY MEMBERS 1 2 7 8  SPOUSE OR PARTNER 1 2 7 8 FRIENDS WHO ARE NOT GAY, LESBIAN, OR BISEXUAL 1 2 7 8 FRIENDS WHO ARE GAY, LESBIAN, OR BISEXUAL 1 2 7 8 HEALTH CARE PROVIDER 1 2 7 8 OTHER 1 2 7 8 | IF ‘NO ONE’ NO OTHER CATEGORY CAN BE CHOSEN.  SKIP FOR TG |  |  |  |  |  |  |  |  |  |  |
| 25 | TGLive | [In the past 6 months, have you ever lived as a woman? By living as a woman, I mean dressing and presenting yourself as a woman.](javascript:__doPostBack('ctl00$ContentPlaceHolder1$dgdQuestion$ctl06$ctl01','')) | NO ONE 1 2 7 8  SPOUSE 1 2 7 8  CLIENT 1 2 7 8  DOCTOR 1 2 7 8  FRIEND – MSM/LESBIAN/TG 1 2 7 8  FRIEND – Heterosexual 1 2 7 8  PARENTS / AUNTS /UNCLES  SIBLINGS/COUNSINS  CHILD/CHILDREN  1 2 7 8  PASTOR/PRIEST 1 2 7 8 | SKIP IF NOT TG |  |  |  |  |  |  |  |  |  |  |
| 26 | TGINFO | Which of the following people have you told about your transgender identity?  CHECK ALL THAT APPLY | NO ONE 1 2 7 8  SPOUSE 1 2 7 8  CLIENT 1 2 7 8  DOCTOR 1 2 7 8  FRIEND – FELLOW SEX WORKER 1 2 7 8  FRIEND – NOT INVOLVED IN SEX WORK 1 2 7 8  PARENTS / AUNTS /UNCLES  SIBLINGS/COUNSINS  CHILD/CHILDREN  1 2 7 8  PASTOR/PRIEST 1 2 7 8 | SKIP IF NOT TG |  |  |  |  |  |  |  |  |  |  |
| 27 | TGHORM | Which type of hormones have you used?  CHECK ALL THAT APPLY. | NONE A  INJECTION B  GEL C  PILLS D  PATCH E  OTHER X  DON’T KNOW Y  REFUSED Z | IF ‘NONE’ NO OTHER CATEGORY CAN BE CHOSEN.  SKIP IF NOT TG |  |  |  |  |  |  |  |  |  |  |
| 28 | TGINJ | In the last 6 months, how often did you get hormone injections? | NEVER 1 AT LEAST ONCE A DAY 2 ONCE A WEEK 3 ONCE A MONTH 4  DON’T KNOW 7 REFUSED 8 | ONLY IF INJECTED HORMONES.  SKIP IF NOT TG |  |  |  |  |  |  |  |  |  |  |
| 29 | TGSHARE | In the last 6 months, with how many people did you use a needle after someone else had injected hormones with it? | #PEOPLE\|__\|__\|  CODE ‘00’ IF NONE  DON’T KNOW 97  REFUSED 98  MIN: 0  MAX: 95 | ONLY IF INJECTED HORMONES.  SKIP IF NOT TG |  |  |  |  |  |  |  |  |  |  |
|  | TGPROF | Does a health professional know about or supervise your use of hormones? | YES 1  NO 2  DON’T KNOW 7  REFUSED 8 | SKIP IF NOT TG |  |  |  |  |  |  |  |  |  |  |
| 30 | SOMACCEP | About your family: Would you say that your family accepts that you have sex with men/TG, doesn’t know about it, or rejects you for it? | ACCEPTS ME 1  DOESN’T KNOW 2  REJECTS ME 3  DON’T KNOW 7  REFUSE TO ANSWER 8 |  |  |  |  |  |  |  |  |  |  |  |
| **ALCOHOL** | | | | | |  |  |  |  |  |  |  |  |  |
| NA | AL1MSG | Now we are going to ask you some questions about your intake of alcoholic beverages during this past year. | | | |  |  |  |  |  |  |  |  |  |
| 31 | ALFRQ | How often do you have a drink containing alcohol? | NEVER 0  MONTHLY OR LESS 1 2-4 TIMES A MONTH 2 2-3 TIMES A WEEK 3 4 OR MORE TIMES A WEEK 4 REFUSE TO ANSWER 8 | ‘0’🡪SKIP TO END OF SECTION | AUDIT-C/AUDIT |  |  |  |  |  |  |  |  |  |
| 32 | ALDAY | How many drinks containing alcohol do you have on a typical day when you are drinking? | 1 OR 2 0  3 OR 4 1  5 OR 6 2 7-9 3 10 OR MORE 4 REFUSE TO ANSWER 8 |  | AUDIT-C/AUDIT |  |  |  |  |  |  |  |  |  |
| 33 | ALBNGE | On one occasion, how often do you have six or more drinks? | NEVER 0  LESS THAN MONTHLY 1 MONTHLY 2 WEEKLY 3 DAILY OR ALMOST DAILY 4 REFUSE TO ANSWER 8 | IF ALDAY=’0’ AND ALBINGE = ‘0’→SKIP TO END OF SECTION | AUDIT-C/AUDIT |  |  |  |  |  |  |  |  |  |
| 34 | ALCTRL | During the last year, how often have you found that you were not able to stop drinking once you had started? | NEVER 0  LESS THAN MONTHLY 1  MONTHLY 2  WEEKLY 3  DAILY OR ALMOST DAILY 4  REFUSE TO ANSWER 8 |  |  |  |  |  |  |  |  |  |  |  |
| 35 | ALFAIL | During the last year, how often have you failed to do what was normally expected of you because of drinking? | NEVER 0  LESS THAN MONTHLY 1  MONTHLY 2  WEEKLY 3  DAILY OR ALMOST DAILY 4  REFUSE TO ANSWER 8 |  |  |  |  |  |  |  |  |  |  |  |
| 36 | ALMORN | During the last year, how often have you needed a first drink in the morning to get yourself going after a heavy drinking session? | NEVER 0  LESS THAN MONTHLY 1  MONTHLY 2  WEEKLY 3  DAILY OR ALMOST DAILY 4  REFUSE TO ANSWER 8 |  |  |  |  |  |  |  |  |  |  |  |
| 37 | ALGUILT | During the last year, how often have you had a feeling of guilt or remorse after drinking? | NEVER 0  LESS THAN MONTHLY 1  MONTHLY 2  WEEKLY 3  DAILY OR ALMOST DAILY 4  REFUSE TO ANSWER 8 |  |  |  |  |  |  |  |  |  |  |  |
| 38 | ALMEM | During the last year, how often have you been unable to remember what happened the night before because of your drinking? | NEVER 0  LESS THAN MONTHLY 1  MONTHLY 2  WEEKLY 3  DAILY OR ALMOST DAILY 4  REFUSE TO ANSWER 8 |  |  |  |  |  |  |  |  |  |  |  |
| 39 | ALINJur | Have you or someone else been injured because of your drinking? | NO 0  YES, BUT NOT IN THE LAST YEAR 1  YES, DURING THE LAST YEAR 3  REFUSE TO ANSWER 8 |  |  |  |  |  |  |  |  |  |  |  |
| 40 | ALCNCRN | Has a relative, friend, doctor, or other health care worker been concerned about your drinking or suggested you cut down? | NO 0  YES, BUT NOT IN THE LAST YEAR 1  YES, DURING THE LAST YEAR 3  REFUSE TO ANSWER 8 |  |  |  |  |  |  |  |  |  |  |  |
| **NON-INJECTION DRUG USE** | | | | | |  |  |  |  |  |  |  |  |  |
| NA | DU1MSG | The next few questions are on the use of any drugs that you may have used without injecting. Such drugs may be smoked, inhaled or snorted. These include drugs like marijuana, crystal meth, cocaine, crack, ecstasy, heroin, or opium [insert drugs from local context]. | | | |  |  |  |  |  |  |  |  |  |
| 41 | DUEVER | Did you ever smoke, inhale or snort crystal meth, cocaine, crack, ecstasy, heroin, marijuana or opium? | YES 1 NO 2 DON’T KNOW 7 REFUSE TO ANSWER 8 | ‘2’,’7’ OR ‘8’ →SKIP TO END OF SECTION |  |  |  |  |  |  |  |  |  |  |
| 42 | DU6MO | In the last 6 months, have you smoked, inhaled or snorted marijuana, crystal, meth, cocaine, crack, ecstasy, heroin or opium? | YES 1 NO 2 DON’T KNOW 7 REFUSE TO ANSWER 8 | ‘2’,’7’ OR ‘8’ →SKIP TO END OF SECTION |  |  |  |  |  |  |  |  |  |  |
| **INJECTION DRUG USE** | | | | | |  |  |  |  |  |  |  |  |  |
|  |  | The next set of questions is about injection drug use. This means injecting yourself with drugs to get high, including drugs that are not prescribed for you or pres. | | | |  |  |  |  |  |  |  |  |  |
| 43 | IDEVER | Have you ever injected any drugs other than those prescribed for you? By injecting, I mean anytime you have used any illegal or illicit drugs to get high. | YES 1 NO 2 DON’T KNOW 7 REFUSE TO ANSWER 8 | ‘2’, ‘7’ OR ‘8’ →SKIP TO END OF SECTION |  |  |  |  |  |  |  |  |  |  |
| 44 | ID6MOS | In the last 6 months, have you injected any illicit or illegal drugs? | YES 1 NO 2 DON’T KNOW 7 REFUSE TO ANSWER 8 | ‘2’, ‘7’ OR ‘8’→SKIP TO END OF SECTION |  |  |  |  |  |  |  |  |  |  |
| **SOCIAL COHESION** | | | | | |  |  |  |  |  |  |  |  |  |
|  |  | The next several questions are about your social life and your relationships with other sex workers. Please mark if you strongly disagree, disagree, are neutral, agree, or strongly agree with the statements. | | | |  |  |  |  |  |  |  |  |  |
| 45 | SCDOC | You can count on other MSM/TG to accompany you to the doctor or hospital. | STRONGLY DISAGREE 1  DISAGREE 2  NEUTRAL 3  AGREE 4  STRONGLY AGREE 5  DON’T KNOW 9997  REFUSE TO ANSWER 9998 |  |  |  |  |  |  |  |  |  |  |  |
| 46 | SCVIOL | You can count on other MSM/TG to help you deal with a violent or difficult /person in your life. | STRONGLY DISAGREE 1  DISAGREE 2  NEUTRAL 3  AGREE 4  STRONGLY AGREE 5  DON’T KNOW 9997  REFUSE TO ANSWER 9998 |  |  |  |  |  |  |  |  |  |  |  |
| 47 | SCSUP | You can count on other MSM/TG to support your use of condoms. | STRONGLY DISAGREE 1  DISAGREE 2  NEUTRAL 3  AGREE 4  STRONGLY AGREE 5  DON’T KNOW 9997  REFUSE TO ANSWER 9998 |  |  |  |  |  |  |  |  |  |  |  |
|  | SCPOLI | In the past 12 months, have you negotiated with or stood up against police in order to help an MSM/TG? | YES 1  NO, NOT PART OF SITUATOIN 2  NO, PART OF SITUATION BUT DID NOT SUPPORT 3  DON’T KNOW 7  REFUSE TO ANSWER 8 |  |  |  |  |  |  |  |  |  |  |  |
| **SEXUAL HISTORY** | | | | | |  | | |  | | |  | | |
| NA |  | The next few questions are about your lifetime sexual history. This includes vaginal and anal sex. With vaginal sex we mean a penis enters a vagina. With anal sex we mean a penis enters a person’s anus (butt). | | | |  | | |  |  |  |  |  |  |
| NA |  | First we will ask you some questions about your sexual experiences with women. | | | |  | | |  |  |  |  |  |  |
| 48 | LIMFVAG | Have you ever had vaginal sex? By vaginal sex we mean your penis enters a woman’s vagina. | YES 1 NO 2 DON’T KNOW 7 REFUSE TO ANSWER 8 |  |  |  |  |  |  |  |  |  |  |  |
| 49 | LIMFANAL | Have you ever had anal sex with a woman? By anal sex I you put your penis into a woman’s anus. | YES 1 NO 2 DON’T KNOW 7 REFUSE TO ANSWER 8 | IF LIMFVAG=’2’,’7’ OR ‘8’ AND LIMFANAL=’2’,’7’ OR ‘8’ → SKIP TO LIMMANAL |  |  |  |  |  |  |  |  |  |  |
| NA |  | Now we will ask you about your sexual experiences with men. | | | |  |  |  |  |  |  |  |  |  |
| 50 | LIMMANAL | Have you ever had anal sex with a man/TG? | YES 1  NO 2  DON’T KNOW 7  REFUSE TO ANSWER 8 | ‘2’,’7’ OR ‘8’→ SKIP TO END OF SECTION |  |  |  |  |  |  |  |  |  |  |
| NA |  | Please think back to the first time you had anal sex with a man. | | | |  |  |  |  |  |  |  |  |  |
| 51 | limmpart | In your **lifetime** with how many **men/TG** have you had anal sex? | MEN [__\|__\|__\|__]  DON’T KNOW 9997  REFUSE TO ANSWER 9998 |  |  |  |  |  |  |  |  |  |  |  |
|  |  | *Please think back to the first time you had sex with a man/TG.* |  |  |  |  |  |  |  |  |  |  |  |  |
| 52 | limm1age | How old were you when you first had anal sex with a male/TG partner? | YEARS [__\|__]  MAX: CURRENT AGE  DON’T KNOW 97  REFUSE TO ANSWER 98 |  |  |  |  |  |  |  |  |  |  |  |
| 53 | LImmAGPA | Approximately how old was your male/TG sex partner at the time you first had sex?  *PLEASE GIVE YOUR BEST GUESS* | MORE THAN 10 YEARS YOUNGER THAN ME 1  5-10 YEARS YOUNGER THAN ME 2  ABOUT THE SAME AGE 3  5-10 YEARS OLDER THAN ME 4  MORE THAN 10 YEARS OLDER THAN ME 5  DON’T KNOW 7  REFUSE TO ANSWER 8 |  |  |  |  |  |  |  |  |  |  |  |
| 54 |  | The first time you had anal sex, was it because you wanted to or because you were you forced? | WANTED TO = 1  FORCED = 2  DOn’T KNOW = 7  refused = 8 |  |  |  |  |  |  |  |  |  |  |  |
| 55 |  | The first time you had anal sex, were you physically forced or were you pressured into having sex through harassment, threats or tricks? | PHYSICALLY FORCED = 1  PRESSURED = 2  DON’T KNOW = 7  REFUSED = 8 |  |  |  |  |  |  |  |  |  |  |  |
| 56 | LIMMID | How would you describe the first man/TG you had sex with? | BOYFRIEND/PARTNER 1  FRIEND/AQUANTANCE/COWORKER 2  RELATIVE 3  STRANGER 4  ‘AUTHORITY FIGURE’ (GOVERNMENT OFFICIAL, RELIGIOUS LEADER, TECHER, EMPLOYER, MILITARY, POLICE, PRISON GUARD) 5  OTHER 6  DON’T KNOW 7  REFUSE TO ANSWER 8 |  |  |  |  |  |  |  |  |  |  |  |
| 57 | limmget | Did this man/TG pay you or give you something in exchange for sex? | YES 1  NO 2  DON’T KNOW 7  REFUSE TO ANSWER 8 |  |  |  |  |  |  |  |  |  |  |  |
| **RECALL SEXUAL BEHAVIOR** | | | | | |  |  |  |  |  |  |  |  |  |
| NA | RCMSG1 | This next section is about your sexual behavior in the last 6 months. The questions are about different sex partners, how many times you had sex, and condom use. With sex we mean either vaginal sex or anal sex. With vaginal sex we mean a penis enters a vagina. With anal sex we mean a penis enters a person’s anus (butt). We will ask you about four different sex partner types: main sex partners, casual sex partners, people you pay money or give goods for sex, and people who pay you money or give you goods for sex. Each person you have had sex with in the last 6 months should be counted in one of these categories. | | | |  |  |  |  |  |  |  |  |  |
| 58 | RCMAPA | First we will ask you some questions about any male/TG sex partners you may have had. In the last 6 months, did you have anal sex with any males? | YES 1 NO 2 DON’T KNOW 7 REFUSE TO ANSWER 8 | ‘2’,’7’ OR ‘8’→SKIP TO FEMALE PARTNER SECTION |  |  |  |  |  |  |  |  |  |  |
| NA | MN | MAIN MALE SEX PARTNERS | | (SUBSECTION) |  |  |  |  |  |  |  |  |  |  |
| 59 | RCMAMNPA | As mentioned earlier, we will ask you about different types of sex partners. First we will ask you about any main male /TG sex partners you may have had anal sex with in the last 6 months.   A main sex partner is someone you are committed to, for example your spouse, live-in sex partner, or boyfriend. There is no payment or exchange of goods or services gifts, goods or services for sex with these partners.   In the last 6 months, with how many different main male / tg partners did you have sex? Type ‘0’ if none. | [___\|___\|___\|___] DON’T KNOW 9997 REFUSE TO ANSWER 9998 | ‘0’,’9997’ OR ‘9998’→SKIPTO END OF SUBSECTION |  |  |  |  |  |  |  |  |  |  |
| 60 | RCMAMNNU | With how many of these people did you ever not use a condom? | [___\|___\|___\|___] DON’T KNOW 9997 REFUSE TO ANSWER 9998 | <= RCMAMNPA |  |  |  |  |  |  |  |  |  |  |
| 61 | RCMAMNRA | In the last six months with your main male/TG partner, have you been the receptive partner, insertive partner, or both?  Receptive anal sex is when he/partner puts their penis in your anus (butt) and insertive anal sex is when you put your penis in their anus (butt). | RECEPTIVE 1  INSERTIVE 2 BOTH 3 DON’T KNOW 7 REFUSE TO ANSWER 8 |  |  |  |  |  |  |  |  |  |  |  |
| 62 | RCMAMNR | The last time you had anal sex with your main male/TG partner, were you the receptive partner or insertive partner or both?  Receptive anal sex is when he puts his penis in your anus (butt) and insertive anal sex is when you put your penis in his anus (butt). | RECEPTIVE 1  INSERTIVE 2 BOTH 3 DON’T KNOW 7 REFUSE TO ANSWER 8 |  |  |  |  |  |  |  |  |  |  |  |
| 63 | RCMAMNRC | The last time you had sex with a main male/TG partner, did you use a condom? | YES 1  NO 2 DON’T KNOW 7 REFUSE TO ANSWER 8 |  |  |  |  |  |  |  |  |  |  |  |
| 64 | RCMAMNFQ | In the last 6 months, how often did you use condoms with your main male /TG partners? | ALWAYS 1 MOST OF THE TIME 2 SOMETIMES 3 RARELY 4 NEVER 5 DON’T KNOW 7 REFUSE TO ANSWER 8 |  |  |  |  |  |  |  |  |  |  |  |
| NA | CS | CASUAL MALE SEX PARTNERS | |  |  |  |  |  |  |  |  |  |  |  |
|  |  | These next questions are about any casual male/TG partners you may have had sex with in the last 6 months. | | | |  |  |  |  |  |  |  |  |  |
| 65 | RCMACSPA | A casual male/TG partner is a man you have sex with but don’t feel committed to. There is no payment or exchange of goods and services for sex with these partners.   In the last 6 months, with how many different casual male/TG partners did you have sex? Type ‘0’ if none. | [___\|___\|___\|___] DON’T KNOW 9997 REFUSE TO ANSWER 9998 | ‘0’,’9997’ OR ‘9998’→ SKIP TO END OF SUBSECTION |  |  |  |  |  |  |  |  |  |  |
| 66 | RCMACSNU | With how many of these people did you ever not use a condom? | [___\|___\|___\|___] DON’T KNOW 9997 REFUSE TO ANSWER 9998 | <= RCMACSPA |  |  |  |  |  |  |  |  |  |  |
| 67 | RCMACSAC | The last time you had anal sex with your casual male/TG partner, did you have receptive or insertive anal sex?   Receptive anal sex is when he puts his penis in your anus (butt) and insertive anal sex is when you put your penis in his anus (butt). | RECEPTIVE 1  INSERTIVE 2 BOTH 3 DON’T KNOW 7 REFUSE TO ANSWER 8 |  |  |  |  |  |  |  |  |  |  |  |
| 68 | RCMACSAC | The last time you had sex with a casual male/TG partner, did you use a condom? | YES 1 NO 2 DON’T KNOW 7 REFUSE TO ANSWER 8 |  |  |  |  |  |  |  |  |  |  |  |
| 69 | RCMACSFQ | In the last 6 months, how often did you use condoms with casual male/TG partners? | ALWAYS 1 MOST OF THE TIME 2 SOMETIMES 3 RARELY 4 NEVER 5 DON’T KNOW 7 REFUSE TO ANSWER 8 |  |  |  |  |  |  |  |  |  |  |  |
| NA | SW | PARTNER TYPE: MALE/TG SEX WORKER | |  |  |  |  |  |  |  |  |  |  |  |
|  |  | This next section asks about people you pay money to for sex. | | | |  |  |  |  |  |  |  |  |  |
| 70 | RCMASWPA | Now we ask questions about buying sex with money or gifts. In the last 6 months, how many different men/TG did you pay money for give goods, gifts or services to in exchange for sex?   Type ‘0’ if none. | [___\|___\|___\|___] DON’T KNOW 9997 REFUSE TO ANSWER 9998 | ‘0’,’9997’ OR ‘9998’→ SKIP TO END OF SUBSECTION |  |  |  |  |  |  |  |  |  |  |
| 71 | RCMASWNU | With how many of these people did you ever not use a condom? | [___\|___\|___\|___] DON’T KNOW 9997 REFUSE TO ANSWER 9998 | <= RCMASWPA |  |  |  |  |  |  |  |  |  |  |
| 72 | rcmaswac | The last time you had sex with a man/TG you gave money, gifts, goods or services for sex, did you use a condom? | YES 1  NO 2  DON’T KNOW 7  REFUSE TO ANSWER 8 |  |  |  |  |  |  |  |  |  |  |  |
| 73 | CLSreg | Do you usually pay or give goods, gifts and services to the same person or to someone different each time? | YES 1  NO 2  DON’T KNOW 7  REFUSED 8 | Skip if RCMASWPA <2 |  |  |  |  |  |  |  |  |  |  |
| NA |  | PARTNER TYPE: MALE/TG CLIENT | | | |  |  |  |  |  |  |  |  |  |
| 74 | RCMACLPA | Some people get money or goods, services of gifts in exchange for sex. In the last 6 months, how many different men/TG paid you money or gave you gifts, goods or services in exchange for sex? Type ‘0’ if none. | [___\|___\|___\|___] DON’T KNOW 9997 REFUSE TO ANSWER 9998 | ‘0’,’9997’ OR ‘9998’→ SKIP TO END OF SUBSECTION |  |  |  |  |  |  |  |  |  |  |
| 75 | RCMACLNU | With how many of these people did you ever not use a condom? | [___\|___\|___\|___] DON’T KNOW 9997 REFUSE TO ANSWER 9998 | <= RCMACLPA |  |  |  |  |  |  |  |  |  |  |
| 76 | RCMAMNRB | In the last six months have you been the receptive or insertive partner, or both, with someone who gave you money or goods in exchange for sex?  Receptive anal sex is when he puts his penis in your anus (butt) and insertive anal sex is when you put your penis in his anus (butt). | RECEPTIVE 1  INSERTIVE 2 BOTH 3 DON’T KNOW 7 REFUSE TO ANSWER 8 |  |  |  |  |  |  |  |  |  |  |  |
| 77 | RCMACLAC | The last time you had anal sex with any male/tg who gave you money or goods for sex, did you have receptive or insertive anal sex?  Receptive anal sex is when he puts his penis in your anus (butt) and insertive anal sex is when you put your penis in his anus (butt). | RECEPTIVE 1  INSERTIVE 2 BOTH 3 DON’T KNOW 7 REFUSE TO ANSWER 8 |  |  |  |  |  |  |  |  |  |  |  |
| 78 | RCMACLCO | The last time you had sex with a man/TG who gave you money or goods, did you use a condom? | YES 1 NO 2 DON’T KNOW 7 REFUSE TO ANSWER 8 |  |  |  |  |  |  |  |  |  |  |  |
| 79 | RCMACLFQ | In the last 6 months, how often did you use condoms with men/TG who gave you money or good in exchange for sex? | ALWAYS 1 MOST OF THE TIME 2 SOMETIMES 3 RARELY 4 NEVER 5 DON’T KNOW 7 REFUSE TO ANSWER 8 |  |  |  |  |  |  |  |  |  |  |  |
| 80 | ccnum | Think about all the male/TG sex partners you had in the last 6 months. With how many of these do you still have active sexual relationships? With ‘active relationships’ we mean you currently have sex with or expect to have sex again in the near future.  With sex we mean anal sex. | PARTNERS [__\|__\|__]  don’t know 997  REFUSED 998 | ‘0’,’1’, ‘997’ OR ‘998’ 🡪SKIP TO END OF SECTION |  |  |  |  |  |  |  |  |  |  |
| NA | FE | FEMALE PARTNERS | |  |  |  |  |  |  |  |  |  |  |  |
| 81 | RCFEPA | Now there are some questions about any female sex partners you may have had or still do. In the last 6 months, did you have sex with any females? | YES 1 NO 2 DON’T KNOW 7 REFUSE TO ANSWER 8 | ‘2’,’7’ OR ‘8’→SKIP TO END OF SECTION |  |  |  |  |  |  |  |  |  |  |
| NA | MN | MAIN FEMALE SEX PARTNERS | | (SUBSECTION) |  |  |  |  |  |  |  |  |  |  |
| 82 | RCFEMNPA | As mentioned earlier, we will ask about different types of sex partners. First we will ask about any main female sex partners you may have had sex with in the last 6 months.   A main sex partner could be your spouse, live-in sex partner, or girlfriend. There is no payment for sex with these partners.   In the last 6 months, with how many different main female partners did you have sex? Type ‘0’ if none. | [___\|___\|___\|___] DON’T KNOW 9997 REFUSE TO ANSWER 9998 | ‘0’,’9997’ OR ‘9998’→ SKIP TO END OF SUBSECTION |  |  |  |  |  |  |  |  |  |  |
| 83 | RCFEMNNU | With how many of these people did you ever not use a condom? | [___\|___\|___\|___] DON’T KNOW 9997 REFUSE TO ANSWER 9998 | <= RCFEMNPA |  |  |  |  |  |  |  |  |  |  |
| NA | SW | PARTNER TYPE: FEMALE SEX WORKER | |  |  |  |  |  |  |  |  |  |  |  |
| 84 | RCFESWPA | Thank you. Now some questions about buying sex. In the last 6 months how many different women did you pay money for sex? Type ‘0’ if none. | [___\|___\|___\|___] DON’T KNOW 9997 REFUSE TO ANSWER 9998 | ‘0’,’9997’ OR ‘9998’→SKIP TO END OF SUBSECTIONEND OF SUBSECTION |  |  |  |  |  |  |  |  |  |  |
| 85 | RCFESWNU | With how many of these people did you ever not use a condom? | [___\|___\|___\|___] DON’T KNOW 9997 REFUSE TO ANSWER 9998 | <= RCFESWPA |  |  |  |  |  |  |  |  |  |  |
| NA | CL | PARTNER TYPE: FEMALE CLIENTS  (RESPONDENT=SEX WORKER) | |  |  |  |  |  |  |  |  |  |  |  |
| 86 | RCFECLPA | Some people get money or goods in exchange for sex. In the last 6 months how many different women gave you money for sex? Type ‘0’ if none. | [___\|___\|___\|___] DON’T KNOW 9997 REFUSE TO ANSWER 9998 | ‘0’,’9997’ OR ‘9998’ GO TO END OF SUBSECTION |  |  |  |  |  |  |  |  |  |  |
| 87 | RCFECLNU | With how many of these people did you ever not use a condom? | [___\|___\|___\|___] DON’T KNOW 9997 REFUSE TO ANSWER 9998 | <= RCFECLPA |  |  |  |  |  |  |  |  |  |  |
| NA | CS | CASUAL FEMALE SEX PARTNERS | |  |  |  |  |  |  |  |  |  |  |  |
| 88 | RCFECSPA | Now some questions about female casual partners. A female casual partner is man you have sex with but don’t feel committed to. There is no payment for sex with such partners. In the last 6 months how many different female casual partners did you have sex with? Type ‘0’ if none. | [___\|___\|___\|___] ###  DON’T KNOW 9997 REFUSE TO ANSWER 9998 | ‘0’,’9997’ OR ‘9998’→ SKIP TO END OF SUBSECTION |  |  |  |  |  |  |  |  |  |  |
| 89 | RCFECSNU | With how many of these people did you ever not use a condom? | [___\|___\|___\|___] DON’T KNOW 9997 REFUSE TO ANSWER 9998 | <= RCFECSPA |  |  |  |  |  |  |  |  |  |  |
| **CONDOM USE** | | | | | |  |  |  |  |  |  |  |  |  |
| NA | CO1MSG | Now we are going to ask you some questions about condoms and your experience using them | | | |  |  |  |  |  |  |  |  |  |
| 90 | COBRND | What brands of condoms do you prefer to use?  CHECK ALL THAT APPLY. | Y N DK NR BRAND A 1 2 7 8 BRAND B 1 2 7 8 OTHER 1 2 7 8 |  |  |  |  |  |  |  |  |  |  |  |
| 91 | COASKM | Could you ask your main male/TG sex partner to use a condom if you wanted? A main sex partner is someone you are committed to, for example your spouse, live-in sex partner, or boyfriend. There is no payment or exchange of goods or services for sex with these partners. | YES 1 NO 2 DON’T KNOW 7 REFUSE TO ANSWER 8 |  |  |  |  |  |  |  |  |  |  |  |
| 92 | COASKM | Could you ask your main female sex partner to use a condom if you wanted? A main female sex partner is someone you are committed to, for example your spouse, live-in sex partner, or girlfriend. There is no payment or exchange of goods or services for sex with these partners. | YES 1 NO 2 DON’T KNOW 7 REFUSE TO ANSWER 8 |  |  |  |  |  |  |  |  |  |  |  |
| 93 | COLIKELY | Are you more likely to use a condom when a man/TG inserts his penis into your anus (butt) or when you put your penis in his? Or about the same for both? | WHEN HIS PENIS IS IN ME 1 WHEN MY PENIS IS IN HIM/HER 2 EQUALLY LIKELY 3 DON’T KNOW 7 REFUSE TO ANSWER 8 | ONLY ASK IF HAD ANAL SEX FROM LIFETIME SEX MODULE. |  |  |  |  |  |  |  |  |  |  |
| 94 | COFREE | In the last 12 months, have you been given condoms for free? For example, through an outreach service, drop-in center or health clinic. | YES 1 NO 2 DON’T KNOW 7 REFUSE TO ANSWER 8 |  |  |  |  |  |  |  |  |  |  |  |
| 95 | COINFO | In the last 12 months, have you received information on condom use and safe sex? For example, through an outreach service, drop-in center or health clinic. | YES 1 NO 2 DON’T KNOW 7 REFUSE TO ANSWER 8 |  |  |  |  |  |  |  |  |  |  |  |
| **LAST SEX ACT** | | | | | |  |  |  |  |  |  |  |  |  |
| 96 |  | The last time you had sex, did you have anal sex, vaginal sex or both? | VAGINAL SEX 1  ANAL SEX 2  BOTH 3  DON’T KNOW 7  REFUSE TO ANSWER 8 |  |  |  |  |  |  |  |  |  |  |  |
| 97 | LSACON | Did you use a condom the last time you had vaginal sex? | YES 1 NO 2 DON’T KNOW 7  REFUSE TO ANSWER 8 |  |  |  |  |  |  |  |  |  |  |  |
| 98 | LSACON | Did you use a condom the last time you had anal sex? | YES 1 NO 2 DON’T KNOW 7  REFUSE TO ANSWER 8 |  |  |  |  |  |  |  |  |  |  |  |
| **LUBRICANT USE** | | | | | |  |  |  |  |  |  |  |  |  |
| NA | LU1MSG | Some people use lubricants during vaginal or anal sex. With vaginal sex we mean a penis enters a vagina. With anal sex we mean a penis enters a person’s anus. Lubricants make your penis or your partner’s penis more slippery and easier to insert into the vagina or anus. Lubricants also prevent the condom from breaking. Now we will ask you some questions about your use of lubricants in the last 6 months. | | | |  |  |  |  |  |  |  |  |  |
| 99 | LU6LUB | In the last 6 months, have you used a lubricant during anal sex? | YES 1 NO 2 DON’T KNOW 7 REFUSE TO ANSWER 8 | ‘2’,’7’ OR ‘8’→ SKIP TO END OF SECTION |  |  |  |  |  |  |  |  |  |  |
| 100 | LUTYPE | In the last 6 months, which lubricant did you use during anal sex?  CHECK ALL THAT APPLY | Y N DK NR WATER-BASED LUBE, KY JELLY, VENDOME 1 2 7 8 SALIVA 1 2 7 8 VASELINE, POMADE OR OTHER PETROLEUM JELLY PRODUCT 1 2 7 8 BODY LOTION, SHEA NUT BUTTER, OR BABY OIL 1 2 7 8  COOKING OIL, MAYONNAISE, BUTTER OR MARGARINE 1 2 7 8 OTHER 1 2 7 8 | SKIP TO LUFREE IF WATER-BASED LUBRICANT NOT SELECTED. |  |  |  |  |  |  |  |  |  |  |
| 101 | LUFREE | In the last 12 months, have you been given “packets” of lubricant for free? For example, through an outreach service, drop-in center or health clinic. | YES 1 NO 2 DON’T KNOW 7 REFUSE TO ANSWER 8 |  |  |  |  |  |  |  |  |  |  |  |
| **Penile Modification** | | | | | |  |  |  |  |  |  |  |  |  |
| NA |  | Some people do things to change how their penis looks. These next questions ask about that. | | | |  |  |  |  |  |  |  |  |  |
| 102 | PM1CUT | Have you ever cut the skin of your penis? | YES 1 NO 2 DON’T KNOW 7 REFUSE TO ANSWER 8 |  |  |  |  |  |  |  |  |  |  |  |
| 103 | PM2CUT | Why did you cut the skin of your penis?  CHECK ALL THAT APPLY | TO INCRESE SEXUAL PLEASURE FOR MYSELF A  TO INCREASE SEXUAL PLEASURE FOR MY PARTNER B  TO IMPROVE CLEANLINESS / GENITAL HYGINE C  TO PREVENT HIV AND SEXUALLY TRANSMITTED INFECTIONS D  OTHER E  DON’T KNOW F REFUSE TO ANSWER G |  |  |  |  |  |  |  |  |  |  |  |
| 104 | PM3CUT | When did you cut the skin of your penis? | WITHIN THE LAST WEEK 1  WITHIN THE LAST MONTH 2  WITHIN THE LAST 6 MONTHS 3  WITHIN THE LAST YEAR 4  WITHIN THE LAST 2 YEARS 5  DON’T KNOW 7 REFUSE TO ANSWER 8 |  |  |  |  |  |  |  |  |  |  |  |
| 105 | PM4CUT | How old were you when you cut the skin of your penis? | AGE IN COMPLETED YEARS: [__\|__] MIN: 1 MAX: CURRENT YEAR-DEAGEY  DON’T KNOW 97  REFUSE TO ANSWER 98 |  |  |  |  |  |  |  |  |  |  |  |
| 106 | PM5CUT | Who did the cut? | I DID IT MYSELF 1  A FRIEND DID IT 2  VILLAGE CUTTER DID IT 3  A HEALTH WORKER DID IT 4  OTHER 6  DON’T KNOW 7 REFUSE TO ANSWER 8 |  |  |  |  |  |  |  |  |  |  |  |
| 107 | PM6CUT | Where was the cut done? | IN THE VILLAGE / COMMUNITY 1  AT THE HEALTH CENTRE 2  AT THE HOSPITAL 3  AT SCHOOL 4  IN PRISION 5  OTHER 6  DON’T KNOW 7 REFUSE TO ANSWER 8 |  |  |  |  |  |  |  |  |  |  |  |
| 108 | PM7CUT | What was used to do the cut? | RAZOR BLADE 1  TRADITIONAL KNIFE E.G. BAMBOO 2  SURGEON’S KNIFE (SCALPEL) 3 OTHER 6  DON’T KNOW 7 REFUSE TO ANSWER 8 |  |  |  |  |  |  |  |  |  |  |  |
| 109 | PM1INS | Have you ever inserted anything into the skin of your penis? | YES 1 NO 2 DON’T KNOW 7 REFUSE TO ANSWER 8 |  |  |  |  |  |  |  |  |  |  |  |
| 110 | PM2INS | What was inserted? | BALL BEARING 1  COLGATE PLASTIC 2  OTHER 6  DON’T KNOW 7 REFUSE TO ANSWER 8 |  |  |  |  |  |  |  |  |  |  |  |
| 111 | PM3INS | Why did you insert something into the skin of your penis??  CHECK ALL THAT APPLY | TO INCRESE SEXUAL PLEASURE FOR MYSELF A  TO INCREASE SEXUAL PLEASURE FOR MY PARTNER B  TO PUNISH WOMEN C  OTHER D  DON’T KNOW E REFUSE TO ANSWER F |  |  |  |  |  |  |  |  |  |  |  |
| 112 | PM4INS | When did you insert something into the skin of your penis? | WITHIN THE LAST WEEK 1  WITHIN THE LAST MONTH 2  WITHIN THE LAST 6 MONTHS 3  WITHIN THE LAST YEAR 4  WITHIN THE LAST 2 YEARS 5  DON’T KNOW 7 REFUSE TO ANSWER 8 |  |  |  |  |  |  |  |  |  |  |  |
| 113 | PM5INS | How old where you when inserted something into the skin of your penis? | AGE IN COMPLETED YEARS: [__\|__] MIN: 1 MAX: CURRENT YEAR-DEAGEY  DON’T KNOW 97  REFUSE TO ANSWER 98 |  |  |  |  |  |  |  |  |  |  |  |
| 114 | PM6INS | Who did it? | DID IT MYSELF 1  A FRIEND DID IT 2  VILLAGE CUTTER DID IT 3  A HEALTH WORKER DID IT 4  OTHER 6  DON’T KNOW 7 REFUSE TO ANSWER 8 |  |  |  |  |  |  |  |  |  |  |  |
| 115 | PM7INS | Where was it done? | IN THE VILLAGE / COMMUNITY 1  AT THE HEALTH CENTRE 2  AT THE HOSPITAL 3  AT SCHOOL 4  IN PRISION 5  OTHER 6  DON’T KNOW 7 REFUSE TO ANSWER 8 |  |  |  |  |  |  |  |  |  |  |  |
| 116 | PM8INS | What was used to make a cute before the item was inserted into the penis? | RAZOR BLADE 1  TRADITIONAL KNIFE E.G. BAMBOO 2  SURGEON’S KNIFE (SCALPEL) 3 OTHER 6  DON’T KNOW 7 REFUSE TO ANSWER 8 |  |  |  |  |  |  |  |  |  |  |  |
| 117 | PM1INJ | Have you ever injected anything into the skin of your penis? | YES 1 NO 2 DON’T KNOW 7 REFUSE TO ANSWER 8 |  |  |  |  |  |  |  |  |  |  |  |
| 118 | PM2INJ | What was injected? |  |  |  |  |  |  |  |  |  |  |  |  |
| 119 | PM3INJ | Why did you inject something into the skin of your penis?  CHECK ALL THAT APPLY | TO INCRESE SEXUAL PLEASURE FOR MYSELF A  TO INCREASE SEXUAL PLEASURE FOR MY PARTNER B  TO PUNISH WOMEN C  OTHER D  DON’T KNOW E REFUSE TO ANSWER F |  |  |  |  |  |  |  |  |  |  |  |
| 120 | PM4INJ | When did you inject something into the skin of your penis?? | WITHIN THE LAST WEEK 1  WITHIN THE LAST MONTH 2  WITHIN THE LAST 6 MONTHS 3  WITHIN THE LAST YEAR 4  WITHIN THE LAST 2 YEARS 5  DON’T KNOW 7 REFUSE TO ANSWER 8 |  |  |  |  |  |  |  |  |  |  |  |
| 121 | PM5INJ | How old where you when you did this? | AGE IN COMPLETED YEARS: [__\|__] MIN: 1 MAX: CURRENT YEAR-DEAGEY  DON’T KNOW 97  REFUSE TO ANSWER 98 |  |  |  |  |  |  |  |  |  |  |  |
| 122 | PM6INJ | Who did it? | DID IT MYSELF 1  A FRIEND DID IT 2  VILLAGE CUTTER DID IT 3  A HEALTH WORKER DID IT 4  OTHER 6  DON’T KNOW 7 REFUSE TO ANSWER 8 |  |  |  |  |  |  |  |  |  |  |  |
| 123 | PM7INJ | Where was it done? | IN THE VILLAGE / COMMUNITY 1  AT THE HEALTH CENTRE 2  AT THE HOSPITAL 3  AT SCHOOL 4  IN PRISION 5  OTHER 6  DON’T KNOW 7 REFUSE TO ANSWER 8 |  |  |  |  |  |  |  |  |  |  |  |
| 134 | PM8INJ | What was used to inject the substance into the penis? | A SYRINGE OBTAINED FROM A HEALTH FACILITY 1  A SYRINGE OBTAINED FROM A PHARMACY 2  OTHER 6  DON’T KNOW 7 REFUSE TO ANSWER 8 |  |  |  |  |  |  |  |  |  |  |  |
| **SHAME, STIGMA, HARASSMENT & DISCRIMINATION** | | | | | |  |  |  |  |  |  |  |  |  |
| NA |  | Please tell us how strongly you agree or disagree with each statement. | | | |  |  |  |  |  |  |  |  |  |
| 125 | STBLACK | Have you ever been blackmailed by someone because you have sex with other men? | YES 1 NO 2 DON’T KNOW 7 REFUSE TO ANSWER 8 |  |  |  |  |  |  |  |  |  |  |  |
| 126 | STHOLD | Have you ever been arrested and held in a police holding cell because you have sex with men/TG?  Have you ever been arrested and held in a police holding cell because you are a TG? | YES 1 NO 2 DON’T KNOW 7 REFUSE TO ANSWER 8 |  |  |  |  |  | | YES 1 NO 2 DON’T KNOW 7 REFUSE TO ANSWER 8 |  | |  |  |
| 127 | STPRIS | Have you ever been sent to prison because you have sex with men/TG or because you are TG? | YES 1 NO 2 DON’T KNOW 7 REFUSE TO ANSWER 8 |  |  |  |  |  |  |  |  |  |  |  |
| 128 | STGHEAL | Have you ever been treated unfairly or denied health care because you have sex with men? | YES 1 NO 2 DON’T KNOW 7 REFUSE TO ANSWER 8 |  |  |  |  |  |  |  |  |  |  |  |
| 129 | STHIDE | When you seek sexual health care, do you feel you need to hide that you have sex with men? | YES 1 NO 2 DON’T KNOW 7 REFUSE TO ANSWER 8 |  |  |  |  |  |  |  |  |  |  |  |
| 130 | STGARST | Have you ever been arrested because you have sex with men? | YES 1 NO 2 DON’T KNOW 7 REFUSE TO ANSWER 8 |  |  |  |  |  |  |  |  |  |  |  |
| 131 | STGJOB | Have you been terminated from a job because you have sex with men? | YES 1  NO 2  DON’T KNOW 7  REFUSE TO ANSWER 8 |  |  |  |  |  |  |  |  |  |  |  |
| **HIV KNOWLEDGE AND PERCEPTIONS** | | | | | |  |  |  |  |  |  |  |  |  |
| NA | HK1MSG | Thank you. Next we will ask you some questions about what you know about HIV. | | | |  |  |  |  |  |  |  |  |  |
| 132 | HKONEPAR | Can you reduce the risk of HIV by having just one sex partner who is HIV negative and who has no other partners? | YES 1 NO 2 DON’T KNOW 3  REFUSE TO ANSWER 8 |  |  |  |  |  |  |  |  |  |  |  |
| 133 | HKCONUSE | Can a person reduce the risk of getting HIV by using a condom every time they have sex? | YES 1 NO 2 DON’T KNOW 3  REFUSE TO ANSWER 8 |  |  |  |  |  |  |  |  |  |  |  |
| 134 | HKHEALTH | Can a healthy-looking person have HIV? | YES 1 NO 2 DON’T KNOW 3  REFUSE TO ANSWER 8 |  |  |  |  |  |  |  |  |  |  |  |
| 135 | HKMOSBIT | Can a person get HIV from mosquito bites? | YES 1 NO 2 DON’T KNOW 3  REFUSE TO ANSWER 8 |  | Can be replaced by most common misconceptions in local context. |  |  |  |  |  |  |  |  |  |
| 136 | HKSRFOOD | Can a person get HIV by sharing food with someone who is infected? | YES 1 NO 2 DON’T KNOW 3  REFUSE TO ANSWER 8 |  | Can be replaced by most common misconceptions in local context. |  |  |  |  |  |  |  |  |  |
| 137 | HKSEXMW | Do you think it is more likely for someone to get HIV through sex with men, TG or women? | MEN 1 WOMEN 2 TG 3  ABOUT THE SAME 4  DON’T KNOW 5 REFUSE TO ANSWER 8 |  |  |  |  |  |  |  |  |  |  |  |
| 138 | HKSEXK | If a condom is not used, what kind of sex puts you at the most risk for HIV? | ORAL SEX 1  VAGINAL SEX 2  ANAL SEX 3  MUTUAL MASTURBATION 4  DON’T KNOW 5  REFUSE TO ANSWER 8 |  |  |  |  |  |  |  |  |  |  |  |
| 139 | HKSEXAN | If a condom is not used, what kind of anal sex puts you at most risk for HIV | ACTIVE (TOP) ANAL SEX 1  PASSIVE (BOTTOM) ANAL SEX 2  BOTH HAVE SAME RISK 3  BOTH HAVE NO RISK 4  DON’T KNOW 5  REFUSE TO ANSWER 8 |  |  |  |  |  |  |  |  |  |  |  |
| 140 | HKSEXVA | Compared to vaginal sex, how important is it to use condoms for *anal* sex?  CHECK ONLY ONE | Less important 1  Equally important 2  More important 3  Don’t know 4  Refuse to answer 8 |  |  |  |  |  |  |  |  |  |  |  |
| 141 | HKTRT | Is there an effective treatment for HIV/AIDS? | YES 1 NO 2 DON’T KNOW 3  REFUSE TO ANSWER 8 |  |  |  |  |  |  |  |  |  |  |  |
| **SERVICES UPTAKE** | | | | | |  |  |  |  |  |  |  |  |  |
| NA | CS1MSG | We will now ask you about services that peer educators or outreach workers may have given you. A peer educator is someone like you who has been trained in HIV prevention. An outreach worker is someone employed by an organization, government or private agency, who might provide these same services to people like you. | | | |  |  |  |  |  |  |  |  |  |
| 142 | CSOREV | When did a peer educator or outreach worker last talk to you about HIV? | IN THE LAST 30 DAYS 1  IN THE LAST 3 MONTHS 2  IN THE LAST YEAR 3  LONGER THAN A YEAR AGO 4  NEVER 5  DON’T KNOW 7  REFUSE TO ANSWER 8 | Need a skip here for never. |  |  |  |  |  |  |  |  |  |  |
| 143 | CSORPR | What things did the outreach worker give you the last time you met one?  CHECK ALL THAT APPLY. | Y N DK NR  NOTHING 1 2 7 8  CONDOMS 1 2 7 8  LUBRICANTS 1 2 7 8  PAMPHLET OR BROCHURE 1 2 7 8  MEDICINES 1 2 7 8  OTHER 1 2 7 8 |  |  |  |  |  |  |  |  |  |  |  |
| 144 | CSINFLU | Who or what has the most influence over your sexual risk behaviors?  DO NOT READ ANSWERS.  CHECK ONLY ONE | Friends 1  Family 2  Sexual partner 3  Social norms 4  Religion 5  HIV awareness materials 6  Health care agents/providers 7  Other 8  Don’t know 97  Refuse to answer 98 |  |  |  |  |  |  |  |  |  |  |  |
| 145 | CSMSM | Do you think the HIV messages you have seen/heard apply to men who have sex with men/TG? | YES 1 NO 2 DON’T KNOW 7 REFUSE TO ANSWER 8 |  |  |  |  |  |  |  |  |  |  |  |
| 146 | CSYOU | Do you think the HIV messages you have seen/heard apply to you? | YES 1 NO 2 DON’T KNOW 7 REFUSE TO ANSWER 8 |  |  |  |  |  |  |  |  |  |  |  |
| 147 | CSAPPLY | Why do you think the messages do not apply to you? | They are not about MSM or TG 1  They are not about anal sex 2  There is a woman in the message/picture 3  They are about pregnant women 4  Other 5  Don’t know 97  Refuse to answer 98 |  |  |  |  |  |  |  |  |  |  |  |
| A | CT | **Counseling and Testing** |  |  |  |  |  |  |  |  |  |  |  |  |
| NA | CS2MSG | We will now ask you a few questions about HIV testing | | | |  |  |  |  |  |  |  |  |  |
| 148 | CSCTEV | Have you ever been tested for HIV? | YES 1 NO 2 DON’T KNOW 7 REFUSE TO ANSWER 8 | ’1’→SKIP TO CSCTPL1 |  |  |  |  |  |  |  |  |  |  |
| 149 | CSCTNV | What is the main reason you have never tested for HIV? | I FEEL I AM NOT AT RISK FOR HIV 1 FEAR OF POSITIVE RESULT 2  NO TIME TO GET TESTED 3 STIGMA BY HEALTH CARE WORKERS 4  OTHER 6 REFUSE TO ANSWER 8 | SKIP TO END SECTION |  |  |  |  |  |  |  |  |  |  |
| 150 | csctpl2 | Where did you have your last test? | AT MY WORK 1  WHERE I HANG OUT 2  OUTREACH/MOBILE TESTING 3  SCHOOL 4  CHURCH 5  OTHER 7  DON’T KNOW 7  REFUSE TO ANSWER 8 |  |  |  |  |  |  |  |  |  |  |  |
| 151 | CSCTTI | When did you last test for HIV? | IN THE LAST 6 MONTHS 1 BETWEEN 6-12 MONTHS AGO 2 MORE THAN 12 MONTHS AGO 3 DON’T KNOW 7 REFUSE TO ANSWER 8 | If CSCTTI^=3 skip to CSCTPAR |  |  |  |  |  |  |  |  |  |  |
| 152 | CSCNOT | Why have you not tested in the last 12 months? | I FEEL I AM NOT AT RISK FOR HIV 1  FEAR OF POSITIVE RESULT 2  NO MONEY TO GET TESTED 3  NO TIME TO GET TESTED 4  STIGMA BY HEALTH CARE WORKERS 5  OTHER 6  REFUSE TO ANSWER 8 |  |  |  |  |  |  |  |  |  |  |  |
| 153 | CSCTPAR | If you have a main sex partner, did you ever test together with that partner? | I DON’T HAVE A MAIN SEX PARTNER 1  YES – WE TESTED TOGETHER 2  NO – WE NEVER TESTED TOGETHER 3  NO—PARTNER WAS TESTED BUT WITHOUT ME 4  DON’T KNOW 7  REFUSE TO ANSWER 8 | Skip if no main partner |  |  |  |  |  |  |  |  |  |  |
| 154 | CSCTRS | What was the result of your last HIV test? | POSITIVE 1 NEGATIVE 2 UNCLEAR / NEITHER POSITIVE OR NEGATIVE 3 DID NOT RECEIVE RESULT 4 DON’T KNOW 7 REFUSE TO ANSWER 8 |  |  |  |  |  |  |  |  |  |  |  |
| 155 | csctTL | Of the following people, who have you told that you have HIV?  CHECK ALL THAT APPLY. | Y N DK NR  NO ONE 1 2 7 8  SPOUSE/SEX PARTNER 1 2 7 8  DOCTOR 1 2 7 8  FRIEND 1 2 7 8  FAMILY MEMBER 1 2 7 8  OTHER 1 2 7 8 |  |  |  |  |  |  |  |  |  |  |  |
| NA |  | Many people with HIV register with a health care provider to get care. By “care” we mean someone goes for check-ups to a health care provider or gets ARVs for their HIV infection. The next few questions are about the first time you saw a provider for your HIV. | | | |  |  |  |  |  |  |  |  |  |
| 156 | CSLKEV | After learning you had HIV, have you ever received HIV medical care from a doctor, clinical officer or nurse? | YES 1 NO 2 DON’T KNOW 7 REFUSE TO ANSWER 8 | ‘1’→SKIP TO CSLKTY1 |  |  |  |  |  |  |  |  |  |  |
| 157 | CSLKNV | What is the main reason why you have never received HIV medical care from a doctor, clinical officer, or nurse? | FACILITY IS TOO FAR AWAY 1  I DON’T KNOW WHERE TO GET HIV MEDICAL CARE 2  COST OF CARE 3  COST OF TRANSPORT 4  I DO NOT NEED IT / I FEEL HEALTHY / NOT SICK 5  I FEAR PEOPLE WILL KNOW THAT I HAVE HIV IF I GO TO A CLINIC 6  RELIGIONS REASONS 7  I’M TAKING TRADITIONAL MEDICINE 8  OTHER 96  DON’T KNOW 97  REFUSE TO ANSWER 98 |  |  |  |  |  |  |  |  |  |  |  |
| 158 | CSLKTI | What month and year did you first see a doctor, clinical officer or nurse for HIV medical care?  PROVE TO VERIFY DATA | MONTH __ __  DON’T KNOW MONTH 7  REFUSED MONTH 8  YEAR __ __ __ __  DON’T KNOW YEAR 7  REFUSED YEAR 8 |  |  |  |  |  |  |  |  |  |  |  |
| NA | CA | Care |  |  |  |  |  |  |  |  |  |  |  |  |
| NA | CS4MSG | Thank you. The next few questions are about the care you may receive now. | | | |  |  |  |  |  |  |  |  |  |
| 159 | CSCALST | What month and year did you last see a doctor, clinical officer or nurse for HIV medical care? | MONTH __ __  DON’T KNOW MONTH 7  REFUSED MONTH 8  YEAR __ __ __ __  DON’T KNOW YEAR 7  REFUSED YEAR 8 | ‘4’,’7’ OR ’8’→CSTREV |  |  |  |  |  |  |  |  |  |  |
| 160 | CSNOGO | What is the main reason for not seeing a doctor, clinical office or nurse for HIV medical care for more than 6 months? | FACILITY IS TOO FAR AWAY 1  I DON’T KNOW WHERE TO GET HIV MEDICAL CARE 2  COST OF CARE 3  COST OF TRANSPORT 4  I DO NOT NEED IT / I FEEL HEALTHY / NOT SICK 5  I FEAR PEOPLE WILL KNOW THAT I HAVE HIV IF I GO TO A CLINIC 6  RELIGIONS REASONS 7  I’M TAKING TRADITIONAL MEDICINE 8  NO APPOINTMENT SCHEDULED/DID NOT MISS MOST RECENT APPOINTMENT 9  OTHER 96  DON’T KNOW 97  REFUSE TO ANSWER 98 |  |  |  |  |  |  |  |  |  |  |  |
|  |  |  |  |  |  |  |  |  |  |  |  |  |  |  |
| NA |  | We will now ask you a few questions about your “CD4” or “T-cell” count. The CD4 count tells how sick you are with HIV or how weak your immune system is and if you need to take ARVs. | | | |  |  |  |  |  |  |  |  |  |
| 161 | CSCDEV | Have you eer had a CD4 count test?  The CD4 count tells you how sick you are with HIV and if you need to take ARVs or other HIV medications | YES 1 NO 2 DON’T KNOW 7 REFUSE TO ANSWER 8 | ‘2’,’7’,’8’ → SKIP TO csTBsc1 |  |  |  |  |  |  |  |  |  |  |
| 162 | CSCDEVL | What month and year were you last tested for your CD4 count? | MONTH __ __  DON’T KNOW MONTH 7  REFUSED MONTH 8  YEAR __ __ __ __  DON’T KNOW YEAR 7  REFUSED YEAR 8 |  | Tailor time periods to national or program guidelines |  |  |  |  |  |  |  |  |  |
|  |  | The next few questions are about tuberculosis or TB. All people with HIV should be “screened” for TB. With “screening”, we mean health care staff ask you about if you have a cough, fever, or sudden weight loss. | | | |  |  |  |  |  |  |  |  |  |
| 163 |  | At your last HIV medical care visit, were you asked if you had any of the following TB symptoms: cough, fever, night sweats and weight loss? | YES 1 NO 2 DON’T KNOW 7 REFUSE TO ANSWER 8 |  |  |  |  |  |  |  |  |  |  |  |
| 164 |  | In the last 12 months, have you experienced any of the following TB symptoms: cough, fever, night sweats and weight loss? | YES 1 NO 2 DON’T KNOW 7 REFUSE TO ANSWER 8 |  |  |  |  |  |  |  |  |  |  |  |
| 165 |  | In the last 12 months, did you receive a chest x-ray or sputum test to look for TB?  A sputrum test is when the patient has to cough and collect the sample in a cup  SELECT ALL THAT APPLY | CHEST X-RAY A  SPUTRUM TEST B  NONE OF THEST C  DON’T KNOW Y  REFUSE TO ANSWER Z |  |  |  |  |  |  |  |  |  |  |  |
| 166 |  | Have you ever visited a TB clinic for TB diagnosis or treatment? | YES 1 NO 2 DON’T KNOW 7 REFUSE TO ANSWER 8 |  |  |  |  |  |  |  |  |  |  |  |
| 167 |  | Were you tested for HIV at the TB clinic? | YES = 1  NO, WAS NOT TESTED FOR HIV =2  NO, ALREADY HIV POSITIVE = 3  don’t know = 7  REFUSED = 8 |  |  |  |  |  |  |  |  |  |  |  |
| 168 |  | Have you ever been told by a doctor, clinical officer or nurse that you had TB? | YES 1 NO 2 DON’T KNOW 7 REFUSE TO ANSWER 8 |  |  |  |  |  |  |  |  |  |  |  |
| 169 |  | What month and year did a doctor, clinical officer or nurse tell you that you have (had) TB?  RECORD THE MOST RECENT TIME IF DIAGNOSED WITH TB MORE THAN ONCE. | MONTH ___ ___  DON’T KNOW MONTH = 7  REFUSED MONTH = 8  yEAR __ __ __ __  DON’T KNOW YEAR = 7  REFUSED YEAR = 8 |  |  |  |  |  |  |  |  |  |  |  |
| 170 |  | Were you ever treated for TB? | YES 1 NO 2 DON’T KNOW 7 REFUSE TO ANSWER 8 |  |  |  |  |  |  |  |  |  |  |  |
| 171 |  | The last time you were treated for TB, did you complete at least 6 months of treatment? | YES 1 NO 2 DON’T KNOW 7 REFUSE TO ANSWER 8 |  |  |  |  |  |  |  |  |  |  |  |
| 172 | CSTBTR2 | When did you take treatment for TB? | BEFORE I TESTED HIV-POSITIVE 1  AFTER I TESTED HIV-POSITIVE 2  BOTH BEFORE AND AFTER I TESTED HIV-POSITIVE 3  DON’T KNOW 7  REFUSE TO ANSWER 8 |  |  |  |  |  |  |  |  |  |  |  |
| D | TR | Anti-retroviral treatment | | IF CSCTRS<>’1’ SKIP TO NEXT SECTION (SUBSECTION) |  |  |  |  |  |  |  |  |  |  |
| NA |  | ART initiation | |  |  |  |  |  |  |  |  |  |  |  |
| NA |  | Thank you. We will now ask you some questions on anti-retroviral treatment, also called ARVs, to treat HIV. | |  | Term ‘ARV’ should be changed based on local context. |  |  |  |  |  |  |  |  |  |
| 173 | CSTREV | Have you ever taken ARVs, that is, antiretroviral medication, to treat HIV infection? | YES 1  NO 2  DON’T KNOW 7  REFUSE TO ANSWER 8 | ‘1’→ SKIP TO CSTRYY |  |  |  |  |  |  |  |  |  |  |
| 174 |  | What is the main reason you have never taken ARVs? | NOT ELIGIBILE FOR TREATMENT 1  HEALTH CARE PROVIDER DID NOT PRESCRIBE 2  HIV MEDICINES NOT AVAILABLE 3  I FEEL HEALTHY/NOT SICK 4  COST OF MEDICINES 5  COST OF TRANSPORT 6  TAKING TRADITIONAL MEDICATIONS 7  NOT ATTENDING HIV CLINIC 8  OTHER 96  DON’T KNOW 97  REFUSED 98 |  |  |  |  |  |  |  |  |  |  |  |
| 175 | CSTRYy | What month and year did you first start taking ARVs?  PROVE TO VERIFY DATE | MONTH __ __  DON’T KNOW MONTH 7  REFUSED MONTH 8  YEAR __ __ __ __  DON’T KNOW YEAR 7  REFUSED YEAR 8 |  |  |  |  |  |  |  |  |  |  |  |
| 176 |  | What month and year did you last receive ARVs? | MONTH __ __  DON’T KNOW MONTH 7  REFUSED MONTH 8  YEAR __ __ __ __  DON’T KNOW YEAR 7  REFUSED YEAR 8 |  |  |  |  |  |  |  |  |  |  |  |
| 177 | CSMSG | Are you currently taking ARVs, that is, antiretroviral medications?  By currently, I mean that you may have missed some doses but you are still taking ARVs. | YES 1 NO 2 DON’T KNOW 7 REFUSE TO ANSWER 8 |  |  |  |  |  |  |  |  |  |  |  |
| 178 |  | Can you tell me the main reason why you are not currently taking ARVs? | I HAVE TROUBLE TAKING A TABLET EVERY DAY 1  I HAD SIDE EFFECTS 2  FACILITY TOO FAR AWAY FOR ME TO GET MEDICINE REGULARLY 3  COST OF MEDICATIONS 4  COST OF TRANSPORT 5  I FEEL HEALTHY / NOT SICK 6  FACUILITY WAS OUT OF STOCK 7  RELIGIONS REASONS 8  TAKING TRANDITIONAL MEDICATIONS 9  OTHER 96  DON’T KNOW 97  REFUSED TO ANSWER 98 |  |  |  |  |  |  |  |  |  |  |  |
| 179 |  | People sometimes forget to take all of their ARVs every day. In the last 30 days, how many days have you missed taking any of your ARV pills?  CODE ‘00’ IF NONE | NUMBER OF DAYS ___  DON’T KNOW 7 REFUSE TO ANSWER 8 |  |  |  |  |  |  |  |  |  |  |  |
| 180 |  | Are you currently taking Septrin or Cotrimoxazole?  Septrin or cotrimoxazole is a medicine recommended for people with HIV, even if they have not started treatment for HIV. It helps prevent certain infections but it is not treatment for HIV.  By currently, I mean that you may have missed some doses but you are still taking Septrin or Cotrimoxazole. | YES 1 NO 2 DON’T KNOW 7 REFUSE TO ANSWER 8 |  |  |  |  |  |  |  |  |  |  |  |
|  | HBV | Next, we'd like to ask you some questions about your health including hepatitis, meaning inflammation of the liver. | | | |  |  |  |  |  |  |  |  |  |
| 181 | hPvac | Have you ever received the Hepatitis B vaccine? | YES 1  NO 2  DON’T KNOW 7  REFUSE TO ANSWER 8 | ‘2’,’7’ or ‘8’🡪SKIP TO HpBTEST |  |  |  |  |  |  |  |  |  |  |
| 182 | HPBTEST | Have you ever been tested for Hepatitis B? | YES 1  NO 2  DON’T KNOW 7  REFUSE TO ANSWER 8 | ‘2’,’7’ or ‘8’🡪SKIP TO HPCTEST |  |  |  |  |  |  |  |  |  |  |
| 183 | HPTTME | When did you have your most recent Hepatitis B test? | LESS THAN 12 MONTHS AGO 1  MORE THAN 12 MONTHS AND LESS THAN FIVE YEARS AGO 2  MORE THAN FIVE YEARS AGO 3  DON’T KNOW 7  REFUSE TO ANSWER 8 |  |  |  |  |  |  |  |  |  |  |  |
| 184 | hPRSL | What was the result of your last Hepatitis B test? | POSITIVE 1 NEGATIVE 2  DON’T KNOW 7  REFUSE TO ANSWER 8 | ‘2’,’7’ OR ‘8’ 🡪 SKIP TO HPCTEST |  |  |  |  |  |  |  |  |  |  |
| **SEXUALLY TRANSMITTED INFECTIONS** | | | | | |  |  |  |  |  |  |  |  |  |
| 185 | STABNMX | During the last 12 months, have you had an abnormal discharge from your penis? | YES 1 NO 2 DON’T KNOW 7 REFUSE TO ANSWER 8 |  |  |  |  |  |  |  |  |  |  |  |
| 186 | STULCM | During the last 12 months, have you had an ulcer or sore on or near your penis? | YES 1 NO 2 DON’T KNOW 7 REFUSE TO ANSWER 8 |  |  |  |  |  |  |  |  |  |  |  |
| 187 | STPAINUR | In the last 12 months, have you had pain on urination? | YES 1  NO 2  DON’T KNOW 7  REFUSE TO ANSWER 8 |  |  |  |  |  |  |  |  |  |  |  |
| 188 | STPROV | Did you see a healthcare provider because of these symptoms? | YES 1  NO 2  DON’T KNOW 7  REFUSE TO ANSWER 8 | NO, DK, REF TO ALL SYMPTOMS 🡪STHPV  ‘1’🡪SKIP TO STLONG |  |  |  |  |  |  |  |  |  |  |
| 189 | STPHARM | Did you see go to a pharmacy to get treatment? | YES 1  NO 2  DON’T KNOW 7  REFUSE TO ANSWER 8 | SKIP TO STHPV |  |  |  |  |  |  |  |  |  |  |
| **PHYSICAL VIOLENCE** | | | | | |  |  |  |  |  |  |  |  |  |
| NA | PVMSG1 | We would now like you to answer some questions about whether or not certain things have happened to you. Please remember that your answers are confidential. If you would like to speak to a health care worker or social worker or anyone else about your experiences, please let the survey know. | | | |  |  |  |  |  |  |  |  |  |
| 190 |  | Has anyone ever done any of these things to you:   - Punched, kicked, whipped, or beat you with an object - Slapped you, threw something at you that could hurt you, pushed you or shoved you - Choked smothered, tried to drown you, or burned you intentionally - Used or or threatened you with a knife, gun or other weapon? | YES 1  NO 2  DON’T KNOW 7  REFUSE TO ANSWER 8 |  |  |  |  |  |  |  |  |  |  |  |
| 191 |  | Thinking about all these experiences that we just discussed, whether someone has done the following:   - Punched, kicked whipped or beat you with an object - Slapped you, threw something at you that could hurt you, pushed you or shoved you - Choked, smothered, tried to drown you or burned you intentionally - Used or threatened you with a knife, gun or other weapon   Did you try to seek professional help or services for any of these incidents from any of the following?  SELECT ALL THAT APPLY. | I DID NOT TRY TO SEEK HELP = A  HEALTHCARE PROFESSIONAL = B  POLICE OR OTHER SECURITY PERSONNEL = C  SOCIAL WORKER, COUNSELOR OR NON-GOVERNMENTAL ORGANIZATION = D  RELIGIOUS LEADER = E  OTHER = X  DON’T KNOW = Y  REFUSED = Z |  |  |  |  |  |  |  |  |  |  |  |
| 192 |  | What was the main reason that you did not try to seek professional help or services? | DID NOT KNOW SERVICES WERE AVAILABLE = 1  SERVICES NOT AVAILABLE = 2  AFRAID OF GETTING IN TROUBLE = 3  ASHAMED FOR SELF/FAMILY = 4  COULD NOT AFFORD SERVICES = 5  DID NOT THINK IT WAS A PROBLEM = 6  FELT IT WAS MY FAULT = 7  AFRAID OF BEING ABANDONED = 8  DID NOT NEED/WANT SERVICES = 9  AFRAID OF MAKING SITUATION WORSE = 10  OTHER = 96  DON’T KNOW = 97  REFUSED = 98 |  |  |  |  |  |  |  |  |  |  |  |
| **SEXUAL VIOLENCE** | | | | | |  |  |  |  |  |  |  |  |  |
| NA | SVMSG1 | We would like to ask you some questions about sex that may have happened to you. When we say ‘sex’ here we are only talking about vaginal sex and anal sex. With vaginal sex we mean a penis enters a vagina. With anal sex we mean a penis enters a person’s anus (butt).   Sometimes people use lies, tricks, threats or physical force to make someone else have sex with them when they do not want to. For the next few questions, we will ask you about when someone tricked you, lied to you or threatened you to make you have sex with them, and NOT about when someone may have physically forced you to have sex.   Please remember that your answers are confidential. If you would like to speak to a health care worker or social worker or anyone else about your experiences, please let the Interview Facilitator know. | | | |  |  |  |  |  |  |  |  |  |
| 193 | SVCEVFR | In your lifetime, how many times has anyone ever physically forced you to have sex?  CODE ‘00” IF NONE | NUMBER OF TIMES ____  DON’T KNOW 7  REFUSE TO ANSWER 8 | ‘1’,’7’,’8’→ SKIP TO SVRFBEH |  |  |  |  |  |  |  |  |  |  |
| 194 |  | How old were you the first time someone physically forced you to have sex? | AGE IN YEARS ____  DON’T KNOW 7  REFUSE TO ANSWER 8 |  |  |  |  |  |  |  |  |  |  |  |
| 195 |  | The first time someone physically forced you to have sex, was the person male or female? If it was more than one person, what was the sex of the person you knew the best? | MALE 1  FEMALE 2  DON’T KNOW 7  REFUSE TO ANSWER 8 |  |  |  |  |  |  |  |  |  |  |  |
| 196 |  | What was this person’s relationship to you? If it was more than one person, what was the relationship with the person you knew the best? | boyfriend/GIRLFRIEND/LIVE-IN PARTNER/SPOUSE = 1  EX-BOYFRIEND/GIRLFRIEND/PARTNER/SPOUSE = 2  RELATIVE/FAMILY MEMBER = 3  classmate/schoolmate = 4  teacher = 5  police/security officer/military= 6  employer = 7  neighbor = 8  community/  religious leader = 9  friend = 10  stranger = 11  other = 96  DOn’t know = 97  refused = 98 |  |  |  |  |  |  |  |  |  |  |  |
| 197 |  | In the last 12 months, did someone physically force you to have sex? | YES 1 NO 2 DON’T KNOW 7 REFUSE TO ANSWER 8 |  |  |  |  |  |  |  |  |  |  |  |
| 198 |  | In the last 12 months, did a partner physically force you to have sex?  By partner, I mean a live-in partner whether or not you were married at the time. | YES 1  NO, DID NOT FORCE 2  NO, DID NOT HAVE A LIVE-IN PARTNER IN THE LAST 12 MONTHS 3  DON’T KNOW 7  REFUSE TO ANSWER 8 |  |  |  |  |  |  |  |  |  |  |  |
| 199 |  | The last time someone tried to make you have sex against your will but did not succeed, was the person male or female? If it was more than one person, what was the sex of the person you knew the best? | MALE 1  FEMALE 2  DON’T KNOW 7  REFUSE TO ANSWER 8 |  |  |  |  |  |  |  |  |  |  |  |
| 200 |  | The last time this happened, what was your relationship to the person who did this? If it was more than one person, what was your relationship with the person you knew the best? | boyfriend/GIRLFRIEND/LIVE-IN PARTNER/SPOUSE = 1  EX-BOYFRIEND/GIRLFRIEND/PARTNER/SPOUSE = 2  RELATIVE/FAMILY MEMBER = 3  classmate/schoolmate = 4  teacher = 5  police/security officer/military= 6  employer = 7  neighbor = 8  community/  religious leader = 9  friend = 10  stranger = 11  other = 96  DOn’t know = 97  refused = 98 |  |  |  |  |  |  |  |  |  |  |  |
| 201 |  | After any of these unwanted sexual experiences, did you try to seek professional help or services from any of the following?  SELECT ALL THAT APPLY. | I DID NOT TRY TO SEEK HELP = A  HEALTHCARE PROFESSIONAL = B  POLICE OR OTHER SECURITY PERSONNEL = C  SOCIAL WORKER, COUNSELOR OR NON-GOVERNMENTAL ORGANIZATION = D  RELIGIOUS LEADER = E  OTHER = X  DON’T KNOW = Y  REFUSED = Z |  |  |  |  |  |  |  |  |  |  |  |
| 202 |  | What was the main reason that you did not try to seek professional help or services? | DID NOT KNOW SERVICES WERE AVAILABLE = 1  SERVICES NOT AVAILABLE = 2  AFRAID OF GETTING IN TROUBLE = 3  ASHAMED FOR SELF/FAMILY = 4  COULD NOT AFFORD SERVICES = 5  DID NOT THINK IT WAS A PROBLEM = 6  FELT IT WAS MY FAULT = 7  AFRAID OF BEING ABANDONED = 8  DID NOT NEED/WANT SERVICES = 9  AFRAID OF MAKING SITUATION WORSE = 10  OTHER = 96  DON’T KNOW = -8  REFUSED = -9 |  |  |  |  |  |  |  |  |  |  |  |
|  |  | The last set of questions are about where you accessed services in 2014 and any NGO or community organization you might have been a member of. | | | |  |  |  |  |  |  |  |  |  |
| 203 | MQTEST | In 2014, at which of these health facilities did you test for HIV? | Y N DK NR  Poro Sapot Clinic 1 2 7 8  Anglicare Stop AIDS 1 2 7 8 Koki Clinic 1 2 7 8 Hederu Clinic, POM General Hospital 1 2 7 8  Laws Road 1 2 7 8  9 Mile Clinic 1 2 7 8  Kaugere Clinic 1 2 7 8  St Joseph’s Medical Centre 1 2 7 8  St Ferdanaz  1 2 7 8  St John’s, Gerehu 1 2 7 8  6 Mile Clinic 1 2 7 8 |  |  |  |  |  |  |  |  |  |  |  |
| 204 | MQSTD | In 2014, at which of these health facilities did you get treated for an STD? | Y N DK NR  Poro Sapot Clinic 1 2 7 8  Anglicare Stop AIDS 1 2 7 8 Koki Clinic 1 2 7 8 Hederu Clinic, POM General Hospital 1 2 7 8  Laws Road 1 2 7 8  9 Mile Clinic 1 2 7 8  Kaugere Clinic 1 2 7 8  St Joseph’s Medical Centre 1 2 7 8  St Ferdanaz  1 2 7 8  St John’s, Gerehu 1 2 7 8  6 Mile Clinic 1 2 7 8 |  |  |  |  |  |  |  |  |  |  |  |
| 205 | MQMEM | In 2014, did you belong to any of these NGOs or community organizations? | Y N DK NR  Kapul Champions 1 2 7 8  Friends Frangipani 1 2 7 8  Igat Hope 1 2 7 8 |  |  |  |  |  |  |  |  |  |  |  |
| 206 | MQACT | In 2014, did you participate in an activity of any of these NGOs or community organizations? | Y N DK NR  Tingim Laip 1 2 7 8  Poro Sapot 1 2 7 8  Kapul Champions 1 2 7 8  Friends Frangipani 1 2 7 8  Igat Hope 1 2 7 8  Salvation Army 1 2 7 8  Hope Worldwide 1 2 7 8  Four Square / Living light 1 2 7 8  Women in Hope 1 2 7 8  Anglicare Stop AIDS 1 2 7 8  PNG DLA (Development Law Association) 1 2 7 8  Friends Foundation 1 2 7 8 |  |  |  |  |  |  |  |  |  |  |  |
| **QUESTIONNAIRE FEEDBACK** | | | | | |  |  |  |  |  |  |  |  |  |
| NA | IN1MSG |  |  |  |  |  |  |  |  |  |  |  |  |  |
| NA | INEDATE | End Date | <DATE> |  | Date of data collection ends should be on every record. |  |  |  |  |  |  |  |  |  |
| NA | INETIME | End Time | <TIME> |  | Interview end time should be on every questionnaire. |  |  |  |  |  |  |  |  |  |
| NA | INTIME | Number of minutes in survey (QPETIME-QPSTIME |  |  |  |  |  |  |  |  |  |  |  |  |
